# Changes in allele frequencies and genetic architecture due to selection in two pig populations

**DOI:** 10.1186/s12711-024-00941-3

**Published:** 2024-12-17

**Authors:** Yvonne C. J. Wientjes, Katrijn Peeters, Piter Bijma, Abe E. Huisman, Mario P. L. Calus

**Affiliations:** 1 Animal Breeding and Genomics, Wageningen University & Research, 6700 AH Wageningen, The Netherlands; 2 Hendrix Genetics B.V., 5830AC Boxmeer, The Netherlands

## Abstract

**Background:**

Genetic selection improves a population by increasing the frequency of favorable alleles. Understanding and monitoring allele frequency changes is, therefore, important to obtain more insight into the long-term effects of selection. This study aimed to investigate changes in allele frequencies and in results of genome-wide association studies (GWAS), and how those two are related to each other. This was studied in two maternal pig lines where selection was based on a broad selection index. Genotypes and phenotypes were available from 2015 to 2021.

**Results:**

Several large changes in allele frequencies over the years were observed in both lines. The largest allele frequency changes were not larger than expected under drift based on gene dropping simulations, but the average allele frequency change was larger with selection. Moreover, several significant regions were found in the GWAS for the traits under selection, but those regions did not overlap with regions with larger allele frequency changes. No significant GWAS regions were found for the selection index in both lines, which included multiple traits, indicating that the index is affected by many loci of small effect. Additionally, many significant regions showed pleiotropic, and often antagonistic, associations with other traits under selection. This reduces the selection pressure on those regions, which can explain why those regions are still segregating, although the traits have been under selection for several generations. Across the years, only small changes in Manhattan plots were found, indicating that the genetic architecture was reasonably constant.

**Conclusions:**

No significant GWAS regions were found for any of the traits under selection among the regions with the largest changes in allele frequency, and the correlation between significance level of marker associations and changes in allele frequency over one generation was close to zero for all traits. Moreover, the largest changes in allele frequency could be explained by drift and were not necessarily a result of selection. This is probably because selection acted on a broad index for which no significant GWAS regions were found. Our results show that selecting on a broad index spreads the selection pressure across the genome, thereby limiting allele frequency changes.

**Supplementary Information:**

The online version contains supplementary material available at 10.1186/s12711-024-00941-3.

## Background

Most livestock populations have been under selection for a very long time. By selecting in every generation the genetically best individuals to produce the next generation, the population is genetically improving over time. As a result of this selection, considerable improvements in the performances of populations have been obtained [1, 2]. Even though the selection pressure in some populations has been strong, this has not had an observable negative effect on the obtained rates of genetic gain for most traits, as those have been stable for many generations [3–6]. These findings suggest that the applied selection has so far been sustainable, but this might change when selection becomes more and more accurate.

Selection improves the population genetically by increasing the frequency of favorable alleles in the population [7–9]. Allele frequencies constantly change as a result of both drift (i.e., random sampling of alleles transmitted to the next generation) and selection. The stronger the selection pressure on a locus, the stronger the change in allele frequency at that locus [7, 8]. Understanding and monitoring changes in allele frequencies as a result of selection is important to get more insights into the long-term effects of selection. So far, most studies investigating this process have used simulation, in which different selection methods can be compared, and therefore benefit from knowing the exact location and effect of causal loci. Those studies have shown that allele frequency changes of causal loci are larger with more accurate selection [10, 11] and when the number of causal loci is smaller [11], that the selection pressure on a locus depends on its statistical additive effect and its linkage with other loci [10], and that selection increases the loss of favorable alleles when they are in linkage with negative alleles at other loci due to hitchhiking [10–14].

A disadvantage of simulation studies is that they rely on several assumptions regarding the genetic architecture of traits, which is still largely unknown. Therefore, there is a need to study changes in allele frequencies in actual populations under selection. The accumulation of genomic data in the past decade(s) enables the use of single nucleotide polymorphism (SNP) data in actual livestock or plant populations to study the impact of selection on changes at the genomic level. At the moment, only a limited number of studies have investigated changes in the genome in actual populations [15–17]. In general, they showed considerable changes in allele frequencies as a result of selection, which were larger than expected under drift [15, 16]. However, none of the studies have correlated the observed changes in allele frequency over a couple of generations in a breeding population with significant regions in genome-wide association studies (GWAS) of the traits under selection in the same population.

Changes in allele frequencies can change the statistical additive effects of loci when non-additive effects such as dominance and epistasis are present [7, 18–21]. Together with new mutations, this can change the genetic architecture of traits over time [22–24]. Using simulations, we have shown that the change in genetic architecture under selection can be substantial, even over a limited number of generations [25]. This was in agreement with a study on broiler data that showed that the genetic variance explained by a window of the genome can be highly variable across generations [26]. However, not much is known at the moment about the change in genetic architecture over time in actual populations under selection.

Therefore, this study investigated changes in allele frequencies and in Manhattan plots for eight traits in two maternal pig lines from 2015 to 2021. It investigated whether the changes in allele frequencies were related to the GWAS results.

## Methods

### Animals, genotypes, and phenotypes

Data from two closed purebred maternal pig lines were used, which were part of the commercial breeding program of Hypor, the swine brand of Hendrix Genetics. In both lines, animals have been selected for many generations based on a selection index that combines multiple production and reproduction traits. The selection indices were slightly different between the lines, due to small differences in desired gains between the lines. Since 2012, a two-step approach that combines pedigree and genomic data was used to estimate breeding values and select parents. This was replaced by single-step genomic prediction in 2016.

Genotypes were available for 40,075 animals from line A and for 23,487 animals from line B (Tables 1 and 2). All animals were born between 2015 and 2021 and genotyped with either a commercial 50k or 80k SNP chip from Illumina (Illumina, San Diego, USA). During an initial quality control, animals were deleted that showed a pedigree-genotype conflict, that had exactly the same genotype as another animal, or that had > 5% missing SNP genotypes.

**Table 1:** Number of available genotypes and phenotypes per trait and per year for line A

Year	Genotypes	Index	Daily gain	Fat depth	Muscle depth	Number of teats	Total number born first parity	Average birth weight first litter	CV of birth weight first litter	Number of small piglets
2015	3505	3505	3144	3078	3046	3014	1403	838	738	748
2016	5826	5826	5229	5149	5149	4527	2097	1223	1103	1103
2017	6586	6586	5821	5603	5603	4837	2004	1201	1071	1071
2018	7361	7361	6066	5499	5499	5187	2695	1504	1309	1313
2019	6492	6492	5399	5227	5227	5141	2190	970	807	808
2020	7689	7689	6423	5951	5952	5915	2303	1172	1035	1036
2021	2616	2616	1507	1407	1409	2094	0	0	0	0
Total	40,075	40,075	33,589	31,914	31,885	30,715	12,692	6908	6063	6079

**Table 2:** Number of available genotypes and phenotypes per trait and per year for line B

Year	Genotypes	Index	Daily gain	Fat depth	Muscle depth	Number of teats	Total number born first parity	Average birth weight first litter	CV of birth weight first litter	Number of small piglets
2015	921	921	787	762	753	914	406	402	373	375
2016	3670	3670	3239	3223	3206	2496	683	598	523	523
2017	3886	3886	3451	3431	3432	2588	780	747	649	649
2018	4995	4995	3965	3851	3851	2922	1459	1178	1008	1010
2019	4165	4165	3561	3497	3500	2203	800	677	564	564
2020	4140	4140	3574	3471	3472	2535	809	797	545	546
2021	1710	1710	1040	1001	1002	1394	0	0	0	0
Total	23,487	23,487	19,617	19,236	19,216	15,052	4937	4399	3662	3667

To prevent large-scale imputation, only SNPs that were located on both the 50k and 80k chips were used. SNPs that showed too many parent-offspring conflicts in one of the lines, that were not segregating in the dataset that combined both lines, or that had > 5% missing genotypes were deleted. This resulted in a dataset with genotypes on 44,056 autosomal SNPs, of which 44,054 were segregating in line A and 44,000 in line B. After quality control, missing genotypes were imputed using Beagle 5.4 [27].

A pedigree file that included all genotyped animals and that combined both lines was available, which included in total 96,199 animals. The pedigree was very complete, with all parents known for animals born from 2012 onwards. For animals born between 2007 and 2011, > 99% had both parents known.

Phenotypes were available for a subset of 8 traits that were included in the selection index (Tables 1 and 2): daily gain (DG), fat depth (FD), muscle depth (MD), number of teats (nTeats), total number born for the first parity (TNB), average birth weight of the first litter (Avg_BW), coefficient of variation of birth weight of the first litter (CV_BW), and the number of small piglets in the first litter (nSmall). The production traits were available for individuals born between 2015 and 2021, while the reproduction traits were available for individuals born between 2015 and 2020. Moreover, for all genotyped animals, their breeding value for the selection index (i.e., the index on which animals were selected that included several traits of which the mentioned production and reproduction traits are a subset), calculated in February 2023, was available. This index was based on all information available in February 2023 and was, therefore, an updated version in terms of available information of the index used for selection in previous years. It is, however, closely related to the index upon which the animals in the dataset were selected.

### Effective population size

The effective population size in the population (*N*_*e*_) was estimated based on the rate of pedigree inbreeding $(\Delta{f})$ and the generation interval (*L*), being the average age of the parents when offspring are born. To estimate the rate of inbreeding, the average pedigree kinship coefficient (*f*_*t*_) was estimated in each year as half the average off-diagonal elements of the pedigree relationship matrix that included all genotyped animals. Across the years 2015 to 2021, $\text{ln}(1-{f}_{t})$ per year was regressed on year and the estimated regression coefficient $(\hat{b})$ was used to estimate the rate of inbreeding per year as ${\Delta \widehat{{f}_{year}}}=1-{e}^{\hat{b}}$ [28]. The rate of inbreeding per generation was then estimated as ${\Delta \widehat{{f}_{L}}}=L\times{\Delta \widehat{{f}_{year}}}$, where *L* was the average generation interval that was estimated based on the birthdates of all genotyped individuals and their parents in the pedigree. This value was used to estimate *N*_*e*_ as $\widehat{{N}_{e}}=\frac{1}{2{\Delta \widehat{{f}_{L}}}}$ [7].

### Genome-wide association studies (GWAS)

GWAS were performed for each combination of line, birth year, and trait, as well as for each combination of line and trait across all birth years. The GWAS was performed for two reasons: (1) to investigate whether the largest observed allele frequency changes were in regions with a significant GWAS peak for one of the traits under selection, and (2) to investigate how the Manhattan plots changed across years. Given that the number of phenotypes available per year for the reproduction traits (TNB, Avg_BW, CV_BW, nSmall) was too low for line B, these traits were not analyzed per year. For the GWAS, the ‘SNP Snappy’ method of Wombat [29] was used by fitting the following model for all traits and SNPs *i*:

$$\mathbf{y}=\mathbf{Xb}_{\varvec{i}}+{\mathbf{Z}}_{\mathbf{1}}{\mathbf{u}}_{\varvec{i}}+{\mathbf{Z}}_{\mathbf{2}}{\mathbf{a}}_{\varvec{i}}+{\mathbf{w}}_{\varvec{i}}{v}_{i}+{\mathbf{e}}_{\varvec{i}},$$

where **y** is a vector of phenotypes, **b**_***i***_ is a vector with fixed effects with incidence matrix **X**, **u**_***i***_ is a vector with random effects with incidence matrix **Z**_**1**_ (see Table 3 for the fixed and random effects included in the models), **a**_***i***_ is a vector of genomic breeding values with incidence matrix **Z**_**2**_ (**a** ~ *N*(0,$\:{\mathbf{G}\sigma\:}_{A}^{2}$)), where **G** is a genomic relationship matrix and $\:{\sigma\:}_{A}^{2}$ is the additive genetic variance, *v*_*i*_ is the fixed allele substitution effect for SNP *i*, **w**_i_ is the vector of genotypes for SNP *i* (coded as 0, 1 and 2), and **e**_***i***_ is a vector of residuals. Note that the subscript “*i*” for **b**_***i***_, **u**_***i***_, **a**_***i***_, and **e**_***i***_ denote that those effects refer to the model in which SNP *i* was fitted as an additional fixed effect. The Wombat software makes use of the property that incidence matrices **X**, **Z**_**1**,_ and **Z**_**2**_ remain the same for all SNPs, which makes it possible to efficiently estimate effects for all SNPs using the full model with all other fixed and random effects included. Variance components used in the model for the GWAS were obtained from an equivalent single-trait Genomic-relatedness-matrix REsidual Maximum Likelihood (GREML) model in Wombat that used the same fixed and random effects as in the above model but excluding SNP *i*. A Bonferroni correction was applied to set the significance threshold for the GWAS, by using a type-1 error rate of 0.05 and assuming that the number of independent tests was equal to the number of SNPs (~ 44,056). This resulted in declaring −^10^log(p-value) higher than 5.94 as significant. For the most significant SNPs, the genetic variance explained was estimated in each year as $\:2{p}_{i}\left(1-{p}_{i}\right){v}_{i}^{2}$, where *p*_*i*_ is the allele frequency and *v*_*i*_ the estimated allele substitution effect of SNP *i* in the year of interest.

**Table 3:** Fixed and random effects in the model for each trait

Trait	Fixed effects	Random effects
Index	Mean	Animal
Daily gain	Mean + way of test^a^ + sex	Batch + litter + animal
Fat depth	Mean + way of test^a^ + sex + scanning device	Batch + litter + animal
Muscle depth	Mean + way of test^a^ + sex + scanning device	Batch + litter + animal
Number of teats	Mean + sex	Birth-herd-year-season + animal
Total number born first parity	Mean	Herd-year-season + semen-year-season^b^ + animal
Average birth weight first litter	Mean	Herd-year-season + semen-year-season^b^ + animal
CV of birth weight first litter	Mean	Herd-year-season + animal
Number of small piglets	Mean	Herd-year-season + semen-year-season^b^ + animal

^a^Way of test reflects the different housing conditions for testing the animals, e.g., in a group with or without a feeding station

^b^Semen-year-season is defined as the origin of the semen in combination with the location of use, and time (year and season) of semen collection. It is a measure of how fresh the semen is at time of use

A genomic relationship matrix (**G**) was used to account for polygenic relationships between the animals in the above models. This relationship matrix was estimated using information on all SNPs using Calc_grm [30], based on method 1 of VanRaden [31]. We decided to use the genomic relationship matrix instead of the pedigree relationship matrix, because initial results showed that the pedigree relationship matrix resulted in too much genomic inflation, as has been observed in other pig studies [32, 33].

### Gene dropping: allele frequency change under drift

To investigate the contribution of selection and drift to the observed allele frequency changes, the expected distribution of allele frequency changes with pure drift were obtained using gene dropping [34], following [16]. In each simulated gene drop, one single bi-allelic locus with two possible allelic variants was simulated. The two alleles were randomly assigned to the founders in the pedigree (which had unknown parents) based on a set minor allele frequency (MAF). MAF values ranging from 0.01 to 0.5, with steps of 0.01, were used and 1000 replicates were used for each MAF value. The assigned founder alleles were then dropped through the pedigree by randomly transmitting one of the two alleles each parent carries to the offspring following Mendelian principles. Allele frequencies were computed for the genotyped individuals in the pedigree for each birth year and for each line, and these were used to obtain the distribution of allele frequency changes under pure drift. The allele frequency change in the real pig data for each SNP relative to its MAF in 2015 was then compared with its distribution obtained under pure drift, as obtained from the gene dropping simulations to determine the effect of selection beyond drift.

## Results

### Effective population size and variance components

The average generation interval was 1.43 years for line A and 1.42 years for line B. The rate of inbreeding was 0.36% per year in line A and 0.42% per year in line B, which was in agreement with a previous study [35]. The *N*_*e*_ was estimated to be 97 in line A and 83 in line B.

Table 4 shows the estimated genetic and phenotypic variance components with the corresponding heritabilities. Both lines showed very similar heritability estimates for corresponding traits. The production traits DG, FD, and MD showed moderate heritability estimates, which was also the case for nTeats and Avg_BW. The other reproduction traits TNB, CV_BW, and nSmall, showed low heritability estimates.

**Table 4:** Estimates of genetic and phenotypic variances and of heritability by trait and line

Trait	Line A			Line B		
	Genetic variance	Phenotypic variance	Heritability	Genetic variance	Phenotypic variance	Heritability
Index	1032	1349	0.76^a^	1103	1278	0.86^a^
Daily gain (g)	2210	13,923	0.16	3167	16,101	0.20
Fat depth	1.72	4.66	0.37	2.33	6.28	0.37
Muscle depth	8.37	38.53	0.22	6.99	33.52	0.21
Number of teats	0.27	0.78	0.35	0.26	0.80	0.33
Total number born first parity	0.93	10.08	0.09	1.06	10.25	0.10
Average birth weight first litter	0.02	0.06	0.29	0.01	0.05	0.27
CV of birth weight first litter	3.79	43.44	0.09	4.87	39.38	0.12
Number of small piglets	0.31	2.88	0.11	0.33	2.20	0.15

^a^Note that the index is a linear combination of estimated breeding values. Therefore, this estimate should not be interpreted as an ordinary heritability

### Allele frequency changes

**Fig. 1:**
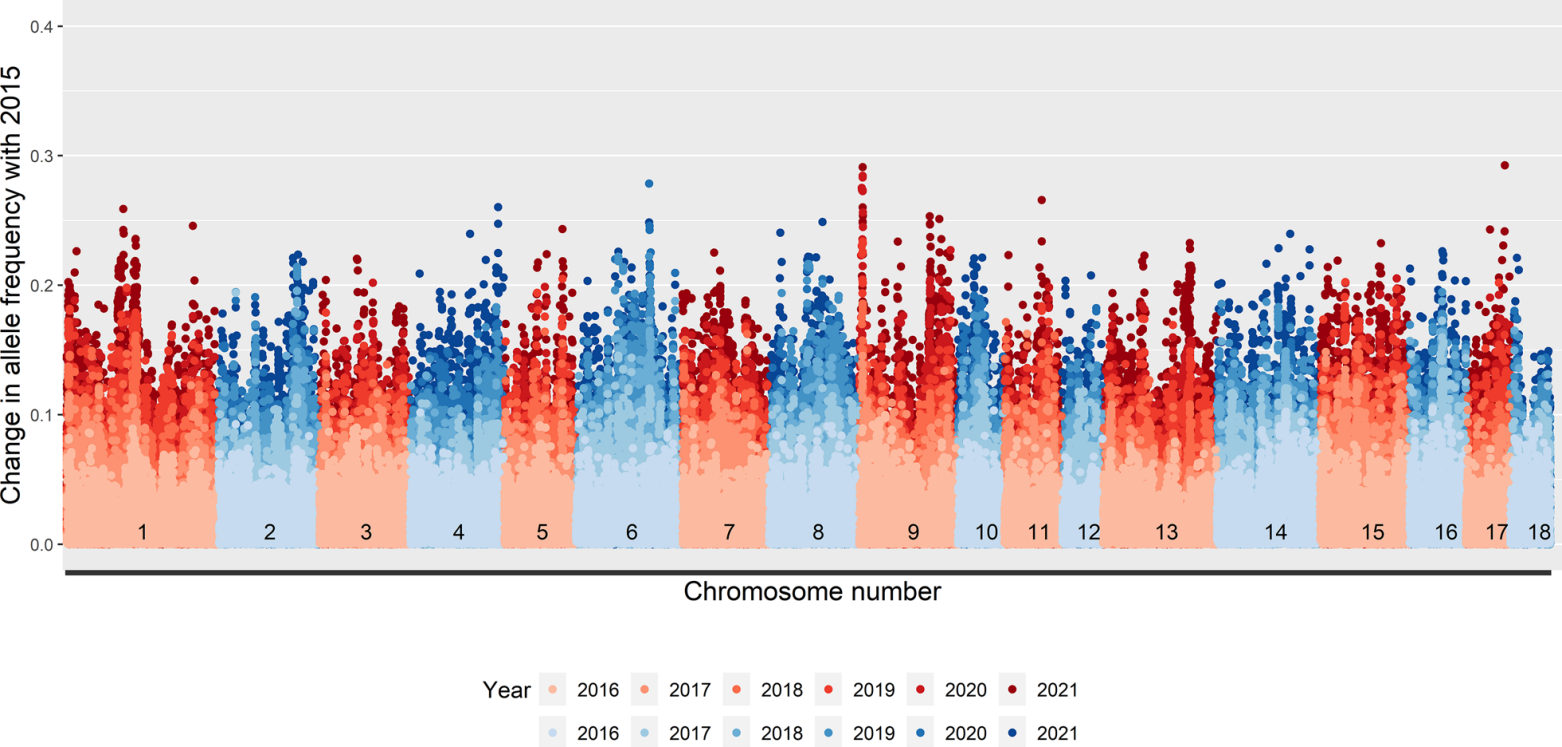
Absolute change in allele frequencies compared to 2015 by genome location in line A

**Fig. 2:**
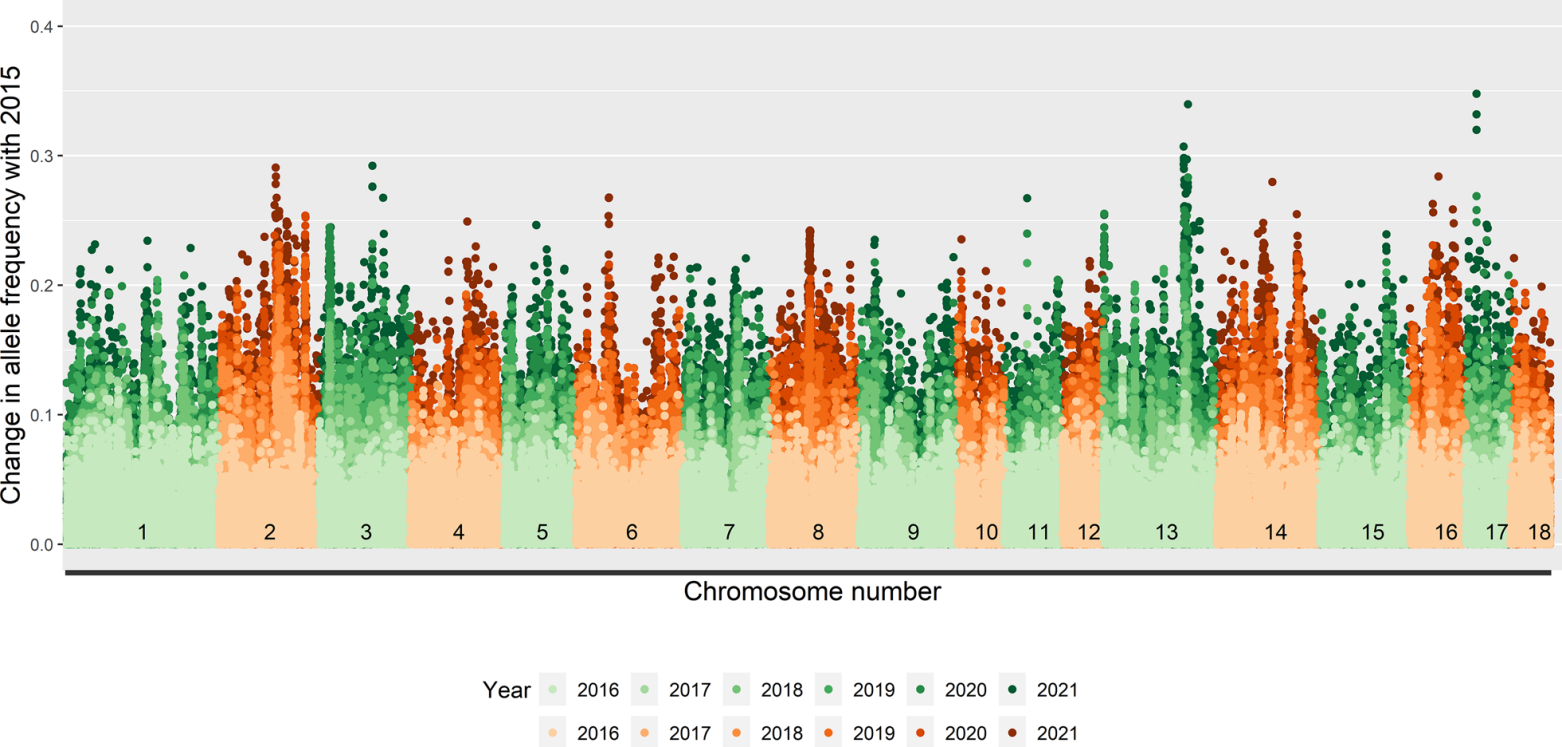
Absolute change in allele frequencies compared to 2015 by genome location in line B

Over the seven years, allele frequencies at the SNPs changed (Figs. 1 and 2). As expected, the absolute changes in allele frequencies increased with length of the time period considered. Several genomic regions that had large changes in allele frequencies were observed, with a maximum change of 0.29 in line A and of 0.35 in line B. For line A, the largest change in allele frequencies was at the start of SSC9. Some other large changes were observed on SSC1, 4, 6, 9, 11, and 17. For line B, the largest changes were observed on SSC13 and 17. Other large changes were observed on SSC2, 3, 6, 11, 14, and 16. There was no overlap in region with the largest allele frequency changes between the two lines, and the correlation between allele frequency changes in the two lines was virtually zero (R^2^ = 0.0006), although both lines were selected based on an index that included the same traits, with only minor differences in desired gains.

The absolute changes in allele frequency increased with MAF of the SNP in 2015 (see Additional file 1: Figures S1.1 and S1.2). For example, the maximum change in allele frequency was only 0.12 in line A and 0.17 in line B for loci with MAF below 0.05 in 2015. Nevertheless, for all MAF levels (i.e., MAF < 0.05, 0.05 < MAF < 0.1, 0.1 < MAF < 0.2, and MAF > 0.2 in 2015), large changes in allele frequencies were observed for several similar regions.

### Genome-wide association study and allele frequency changes

**Fig. 3:**
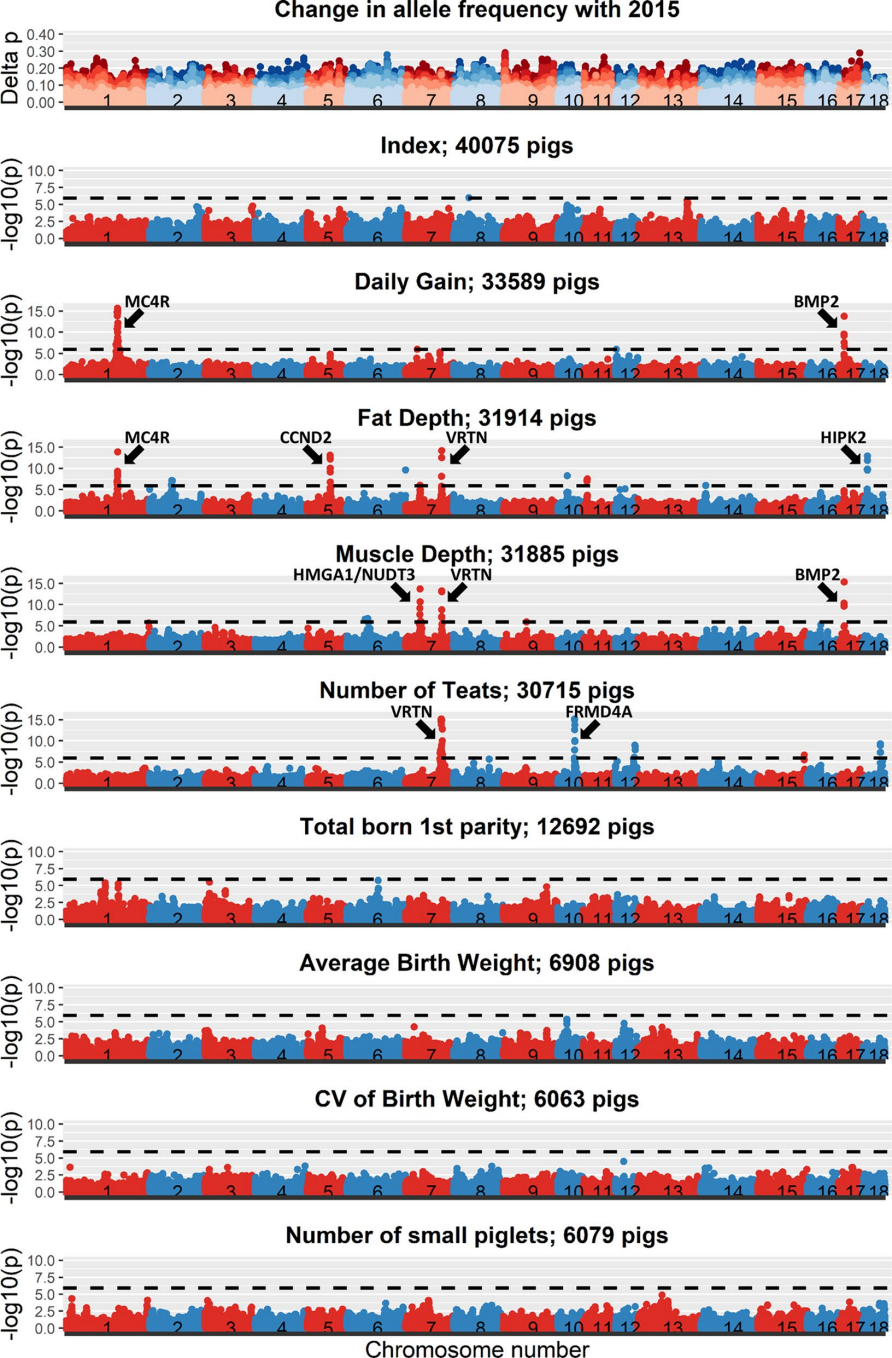
Absolute change in allele frequencies and Manhattan plots for the index and individual traits in line A. The horizontal dotted line represents the significance threshold

**Fig. 4:**
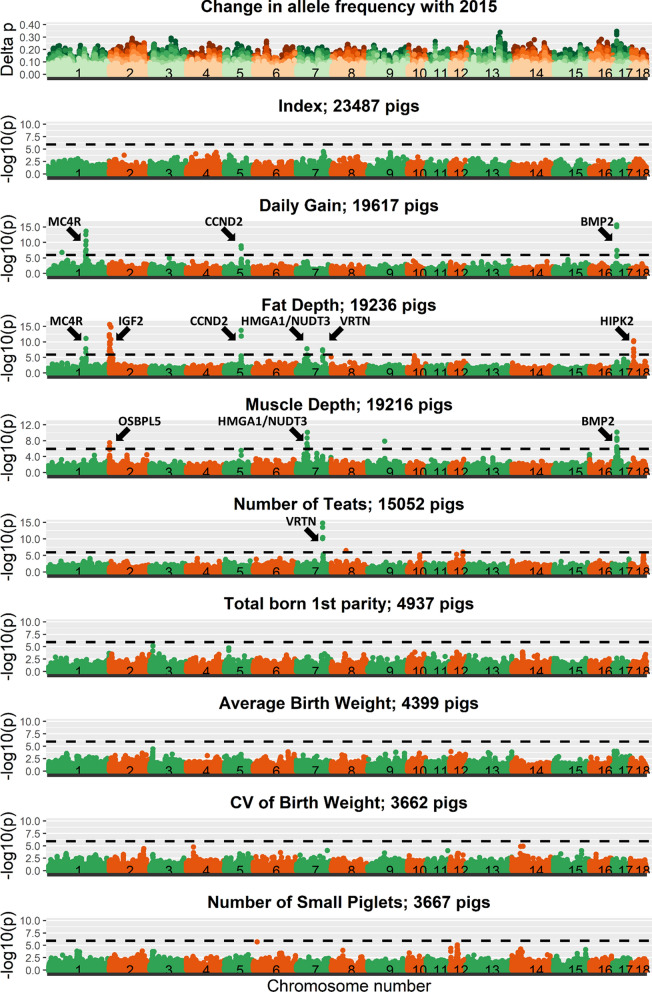
Absolute change in allele frequencies and Manhattan plots for the index and individual traits in line B. The horizontal dotted line represents the significance threshold

The results of the GWAS across birth years for line A are plotted in Fig. 3 and for line B in Fig. 4. Additional file 2 shows the corresponding quantile-quantile (QQ) plots for all GWAS analyses. For DG, FD, MD, and nTeats, some clear peaks of previously described significant regions were found, as indicated in Figs. 3 and 4 [36–46]. Many significant peaks overlapped between the two lines and some regions were significant for multiple traits, such as the *MC4R* region for DG and FD in both lines, the *CCND2* region for DG and FD in line B, the *HMGA1/NUDT3* region for FD and MD in line B, the *VRTN* region for FD, MD, and nTeats in both lines, and the *BMP2* region for DG and MD in both lines. For the reproduction traits (TNB, Avg_BW, CV_BW, nSmall), no significant regions were found. Across all traits, 20 and 11 significant regions were found for line A and line B, respectively, of which 7 regions were significant in both lines.

All the analyzed traits are part of the index used for selection. Although several significant regions were found for the individual production traits, only one SNP, on SSC8, passed the significance threshold for the index for line A and none for line B.

In this study, we were not interested in identifying significant regions, but aimed to understand changes in allele frequency. For the regions with a significant GWAS peak for one of the production traits, no corresponding peak in allele frequency changes was observed (Figs. 3 and 4). To study the link between allele frequency changes and GWAS results in more detail, we also investigated whether the estimated SNP effects or significance levels from GWAS for each trait in a given year were related to the changes in allele frequencies from the current to the next year (see Additional file 3). However, for each year, allele frequency changes at SNPs were completely unrelated to the estimated SNP effects or their significance level, with R^2^ values between 0.000 and 0.004 and regression coefficients between − 0.01 and 0.01. This was also the case for the index. In order to investigate whether this could be the result of SNPs with low MAF, which can only obtain a limited change in allele frequencies in one generation, we also investigated those relationships for SNPs with MAF larger than 0.10. However, even for those SNPs, allele frequency changes were unrelated to their estimated effects or significance levels (see Additional file 4).

### Genome-wide association study across years

**Fig. 5:**
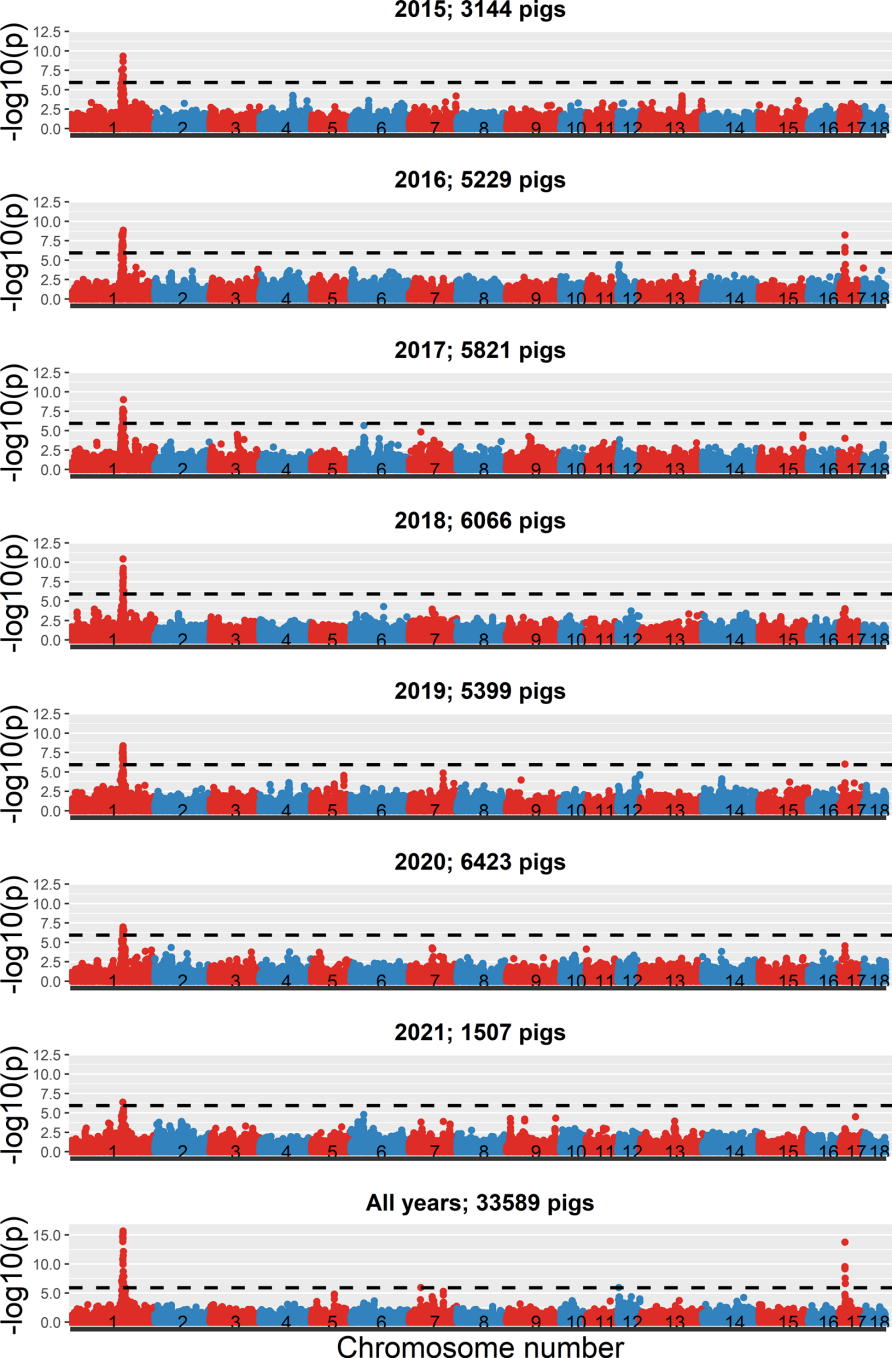
Manhattan plots for daily gain in line A for the different years. The horizontal dotted line represents the significance threshold

**Fig. 6:**
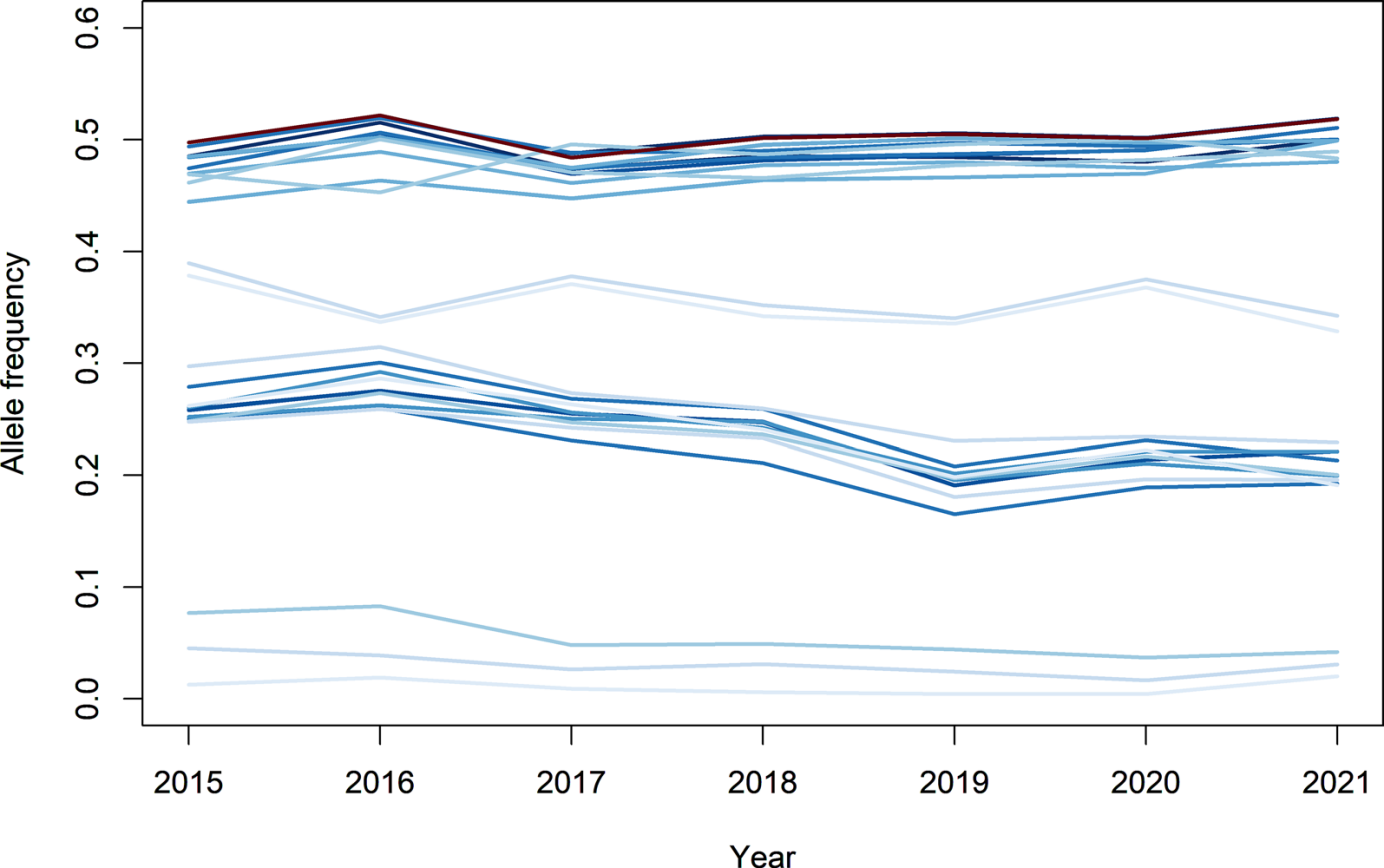
Allele frequency patterns for significant SNPs for daily gain on SSC1 across years in line A. Each line corresponds to a significant SNP for daily gain. The darker the color of the line, the higher the significance value for the SNP, while the red line indicates the most significant SNP in this region. The frequencies for each SNP pertain to the allele that had a frequency below 0.5 in 2015

Another aim of the GWAS was to investigate how the Manhattan plots changed across years, for example due to changes in allele frequencies and effect sizes at causal loci. For DG in line A, the peak on SSC1, related to the *MC4R* region, was present for all years (Fig. 5). However, the height of the peak differed between years and was highest in 2018 and lowest in 2021. The lead SNP in this region was estimated to explain 1.4 to 2.4% of the phenotypic variance for DG. This lead SNP had a significant antagonistic effect on FD, and was not significant for the index. The allele frequencies across years of the significant SNPs in this *MC4R* region (Fig. 6) showed that allele frequencies were relatively constant across years, even for the most significant SNP. This indicates that changes in allele frequencies were not the reason for the differences in significance level. Moreover, it showed that although a significant SNP for DG was found in this region and DG is part of the selection index, the allele frequency patterns in this region showed no evidence of selection.

**Fig. 7:**
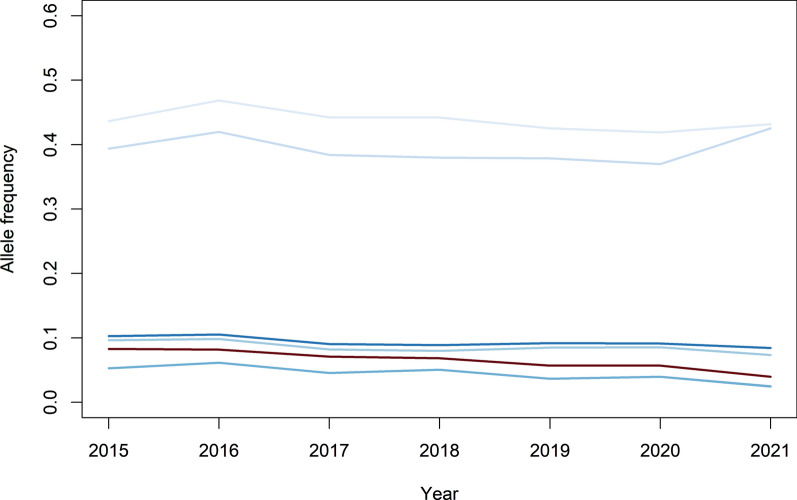
Allele frequency patterns for significant SNPs for daily gain on SSC17 across years in line A. Each line corresponds to a significant SNP for daily gain. The darker the color of the line, the higher the significance value for the SNP, while the red line indicates the most significant SNP in this region. The frequencies for each SNP pertain to the allele that had a frequency below 0.5 in 2015

Besides the peak on SSC1, a significant peak related to the *BMP2* region on SSC17 was found for DG in 2016 and 2019. The lead SNP in this region explained 0.3 to 0.8% of the phenotypic variance in line A. This lead SNP had a significant antagonistic effect on MD, and was not significant for the index. The allele frequencies in this region were relatively stable (Fig. 7), indicating that there was again no evidence of selection in this region.

Besides changes in height of the most significant peaks, Manhattan plots were relatively stable across years. The peaks that were present in the different years were also found when data from all years were combined, where the peaks were in general larger due to more data. So, all in all, there are no indications of very large changes in genetic architecture across years. This same pattern was also observed for the other traits and the other line (see Additional file 5).

### Allele frequency changes due to drift versus selection

**Fig. 8:**
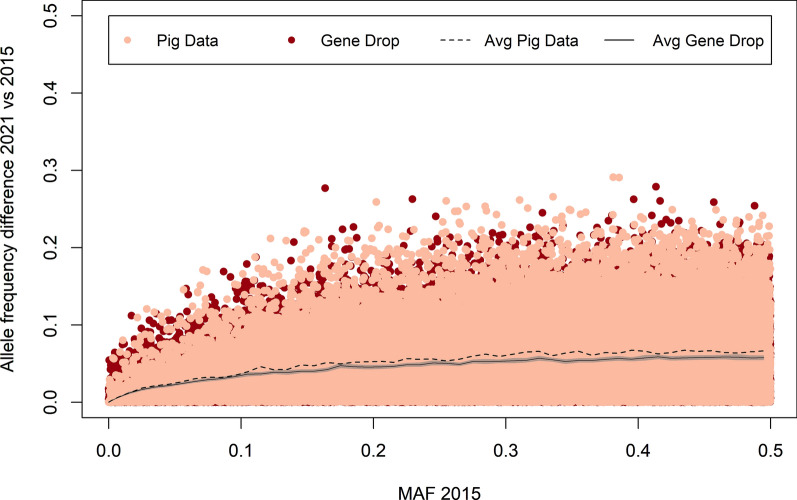
Allele frequency changes obtained with gene dropping and observed in line A. The light grey area represents the 95% confidence interval for the average allele frequency change obtained with gene dropping

**Fig. 9:**
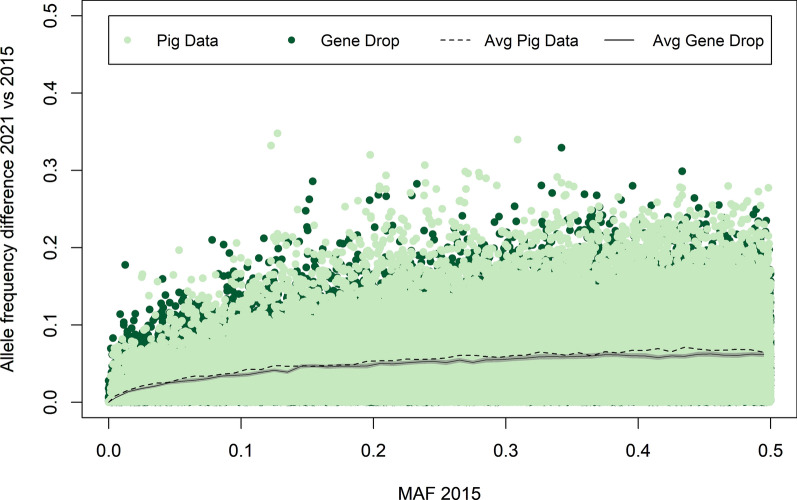
Allele frequency changes obtained with gene dropping and observed in line B. The light grey area represents the 95% confidence interval for the average allele frequency change obtained with gene dropping

Allele frequency changes obtained with gene dropping were compared with the observed allele frequency changes in lines A (Fig. 8) and B (Fig. 9). Both figures show that allele frequency changes of both drift and selection increased with the MAF that the SNP had in 2015. Moreover, the largest allele frequency changes observed from the gene dropping simulation were similar to the largest changes observed in the actual data. This shows that the large changes in allele frequency were not necessarily related to selection but could equally well be a result of drift. Nevertheless, in both lines, the average observed change in allele frequencies was marginally larger than the values obtained with gene dropping. Although these differences were small, they were consistent and significant for most MAF levels in 2015. This was observed for all MAF levels in 2015, except for SNPs with a very low MAF, for which similar changes in allele frequencies were observed with gene dropping and in the actual data.

## Discussion

We investigated changes in SNP allele frequencies and Manhattan plots and how those two are related in two pig populations that have been under selection. We identified several regions with large changes in allele frequencies over seven years of selection in each line, but no significant GWAS peak was found in these regions. Moreover, the largest changes in allele frequencies were not larger than could be expected with drift. For the selection index, no significant GWAS region was found. Altogether, our results indicate that selection acted on a broad (i.e., including production and reproduction traits) and highly polygenic selection index and that genetic gain was achieved by small changes in allele frequencies across very many loci.

### Allele frequency changes

Both populations showed several peaks for allele frequency changes across the genome. Although the selection index included a similar set of traits for the two lines and only differed due to small differences in desired gains, no overlap in allele frequency change peaks was observed between the lines, and the correlation between their allele frequency changes was almost zero (R^2^ = 0.0006). This observation is in agreement with previous results [15], and is probably a result of the high level of polygenicity of the index under selection. Therefore, selection pressure on each locus is low and most allele frequency changes are undirectional and a result of drift [7, 8].

Our results showed that the largest allele frequency changes in the two lines were not larger than expected changes under pure drift. This is in contradiction to previous results in chicken [15] and dairy cattle [16], where selection resulted in slightly larger allele frequency changes than just drift. In the study by Heidaritabar et al. [15], the *N*_*e*_ of the chicken populations under genomic selection (*N*_*e*_: 34–48) were smaller than in our pig populations, while the *N*_*e*_ of the chicken populations under pedigree selection (*N*_*e*_: 83–121) were similar to the *N*_*e*_ in our pig populations. Moreover, the alleles in the gene dropping scenarios all started with an allele frequency of 0.5 and the investigated time frame was only 2 generations. This makes it difficult to compare their results to our study. In the study by Doekes et al. [16], who investigated a cattle population under selection with a similar *N*_*e*_ as observed in our pig populations (*N*_*e*_ estimates ranged between 69 and 102), the gene dropping was done in a similar way as in this study and they also investigated allele frequency changes across ~ 5 generations. This indicates that we need to be careful with extrapolating our results to other populations, as they depend for example on the selection intensity and on polygenicity of the selection index.

### GWAS results for individual traits

Several significant regions were found for the production traits under selection. However, no significant regions were found for the reproduction traits. This is partly related to the lower number of observations for those traits, as they are only recorded on females and later in life. The heritability of those traits is lower as well (Table 4), which makes it more difficult to identify significant regions. Moreover, reproduction traits are in general expected to be highly polygenic and influenced by many loci, each with a small effect [47–51]. So, all in all, it is not surprising that we found no significant regions for reproduction traits.

### Changes in genetic architecture across years

We also investigated how variable the Manhattan plots were across years. Most significant regions were significant in many years, although the height of the significance peak slightly differed between years. Small changes in the estimated effect size of the SNPs and their corresponding significance level could be due to for example non-additivity [18, 19, 25], changes in linkage disequilibrium between the SNP and the causal locus, environmental differences, or due to statistical randomness. However, in general, the observed changes in Manhattan plots were only small. Therefore, we can conclude that the genetic architecture was relatively constant across the investigated time frame of seven years.

### GWAS results for the index

There was only one SNP that passed the significance threshold for the index in line A, with a (− log_10_(p-value) of 5.98, compared to the threshold of 5.94. This SNP explained 0.044% of the genetic variance of the index. Therefore, at least 1/0.00044 = 2274 loci should be underlying the index. Given that all the other SNPs were not significant, they all explained a smaller proportion of the genetic variance and the number of loci underlying the selection index can be expected to be much larger. This is in agreement with a previous suggestion that probably > 1000 loci are underlying the index in livestock breeding populations [9].

The lack of significant SNPs for the index was despite the identification of multiple significant regions for some traits that were part of the index. This can be due to two reasons. The first reason is that the effect of a significant region for a single trait can be diluted in the index. The second reason is that the region can have an antagonistic effect on other traits in the index, thereby removing the significance for the index. This latter reason is supported by the observation that some significant regions were found for multiple traits, such as the *MC4R* region for DG and FD (see Additional file 1: Figure S1.3), the *CCND2* region for DG and FD, the *HMGA1*/*NUDT3* region for FD and MD, the *VRTN* region for FD, MD, and nTeats (see Additional file 1: Figures S1.4, S1.5 and S1.6), and the *BMP2* region for DG and MD (Figs. 3 and 4). The presence of a significant peak in the same region for multiple traits can, however, not differentiate between the presence of a single QTL with antagonistic effects on the two traits or the presence of two strongly linked QTL, one of each trait and with opposite effects. However, some QTL regions were only significant for one trait and were still not significant for the index. For those regions, it can be that a large positive effect for one trait is counteracted by many small negative effects on other traits or that the effect was diluted in the index. Altogether, our results indicate that pleiotropy is abundant in the genome, which is in agreement with previous observations [39, 52, 53], and that the index itself is very polygenic and influenced by many loci with a small effect.

The presence of antagonistic pleiotropy is also expected to be the reason why significant GWAS regions are still segregating in a population, although the traits have been under selection for many generations. This is confirmed by the rather stable allele frequencies across the years for the significant SNPs for DG on SSC1 and SSC17 in line A (Figs. 6 and 7). This means that the identified GWAS peaks can inform us about the biological background of the traits, but may not be helpful to improve our selection approach.

### GWAS results versus allele frequency changes

We compared changes in allele frequencies across the genome with the significant regions identified in the GWAS. In contrast to our expectations, we observed no overlap between the peaks across the genome for allele frequency changes and Manhattan plots (Figs. 1, 2, 3 and 4). Moreover, the correlation between allele frequency changes from one to the next generation and the estimated effect size or significance level of the SNP in that generation was close to zero. A correlation close to zero was also found in a previous simulation study between the statistical additive effect and allele frequency changes over one generation [10]. In that study, allele frequency change was more correlated (correlation around 0.5) with the apparent effect of an allele, estimated as the simple regression of the estimated breeding values on the allele counts of a causal locus. It is good to note that this apparent effect of a locus also included the effects of loci in linkage disequilibrium with that locus and is highly influenced by sampling, especially for loci with a low MAF [10]. In this study, we estimated SNP effects in a GWAS one SNP at a time, while simultaneously fitting a genomic breeding value. In such an analysis, the estimated SNP effects are also influenced by the effects of SNPs in linkage disequilibrium with the SNP of interest but to a lower extent than the apparent effects used in [10]. Moreover, in contrast to [10], we used SNP genotypes instead of genotypes at causal loci, we had to rely on estimated effects instead of actual effects, and the population was selected on an index instead of on a single trait and, therefore, likely influenced by many more causal loci. Those factors together may explain the low correlation between changes in allele frequencies and estimated SNP effects in our study.

The close to zero correlation between estimated effect of a SNP and allele frequency change from one generation to the next does not mean that selection has no effect on allele frequency change across multiple generations. This is because selection is expected to change the allele frequency in the same direction across generations, while drift is undirectional across generations. Methods such as Generation Proxy Selection Mapping [54, 55] that investigates general allele frequency change, and $\hat{G}$ [56] that focusses on genetic gain in a particular trait due to allele frequency change, can be used to investigate the impact of selection on allele frequency change across many generations.

The low correlation between allele frequency changes and estimated effects, in combination with the gene dropping results, suggest that the largest changes in allele frequencies were more related to drift than to selection. This means that genetic gain was not obtained by a large change in allele frequencies at some loci, but by small changes in allele frequencies at many loci. This is supported by the on average larger changes in allele frequencies in the real populations compared to the gene dropping results. The fact that genetic gain in our populations was apparently obtained by small allele frequency changes at many loci is good news, because it means that the selection pressure is spread across the genome, which limits the negative impact of genetic hitchhiking [11, 57].

## Conclusions

We observed several peaks of allele frequency changes across the genome over 7 years of selection in two maternal pig lines. Those peaks were, however, not larger than expected from drift, although the average change in allele frequencies was slightly higher with selection than with pure drift. Using GWAS, we found several previously identified significant regions for the production traits that have been under selection, but in general the GWAS results were not related to the allele frequency change results. Many of the significant GWAS regions for individual traits showed pleiotropic, and probably antagonistic, effects on other traits. The GWAS results showed only some small changes in significant regions across the years, indicating that the genetic architecture was relatively constant across the seven years that we investigated. For the selection index, no significant GWAS regions were found, which shows that the index was very polygenic, which resulted in spreading the selection pressure across the genome. Altogether, we can conclude that genetic gain was obtained by small changes in allele frequencies at many loci.

## Supplementary Information

Additional file 1. Six additional figures related to the manuscript.

Additional file 2. Quantile–Quantile (QQ) plots of the GWAS analyses. Eighteen figures with the QQ plots for the different analyses.

Additional file 3. Correlation allele frequency change and GWAS results. Fourteen figures describing the correlation between allele frequency change and GWAS results (estimated effect and significance level).

Additional file 4. Correlation allele frequency change and GWAS results for loci with MAF > 0.1. Fourteen figures describing the correlation between allele frequency change and GWAS results (estimated effect and significance level) for the loci with a minor allele frequency above 0.1.

Additional file 5. GWAS results across years. Thirteen figures with the Manhattan plots for the different GWAS analyses across the different years.

## Data Availability

The data that support the findings of this study are available from Hendrix Genetics B.V. but restrictions apply to the availability of these data, which were used under license for the current study, and so are not publicly available. Data are however available from the authors upon reasonable request and with permission of Hendrix Genetics B.V.
