# Supplementary material for: Changes in allele frequencies and genetic architecture due to selection in two pig populations
Source: Genet Sel Evol. 2024 Dec 17;56:76. doi: 10.1186/s12711-024-00941-3 (PMC11650847; doi:10.1186/s12711-024-00941-3)
Supplement: Supplementary file 1 — Additional file 1. Six additional figures related to the manuscript. [file 12711_2024_941_MOESM1_ESM.docx]

**Additional file 1: Additional figures**


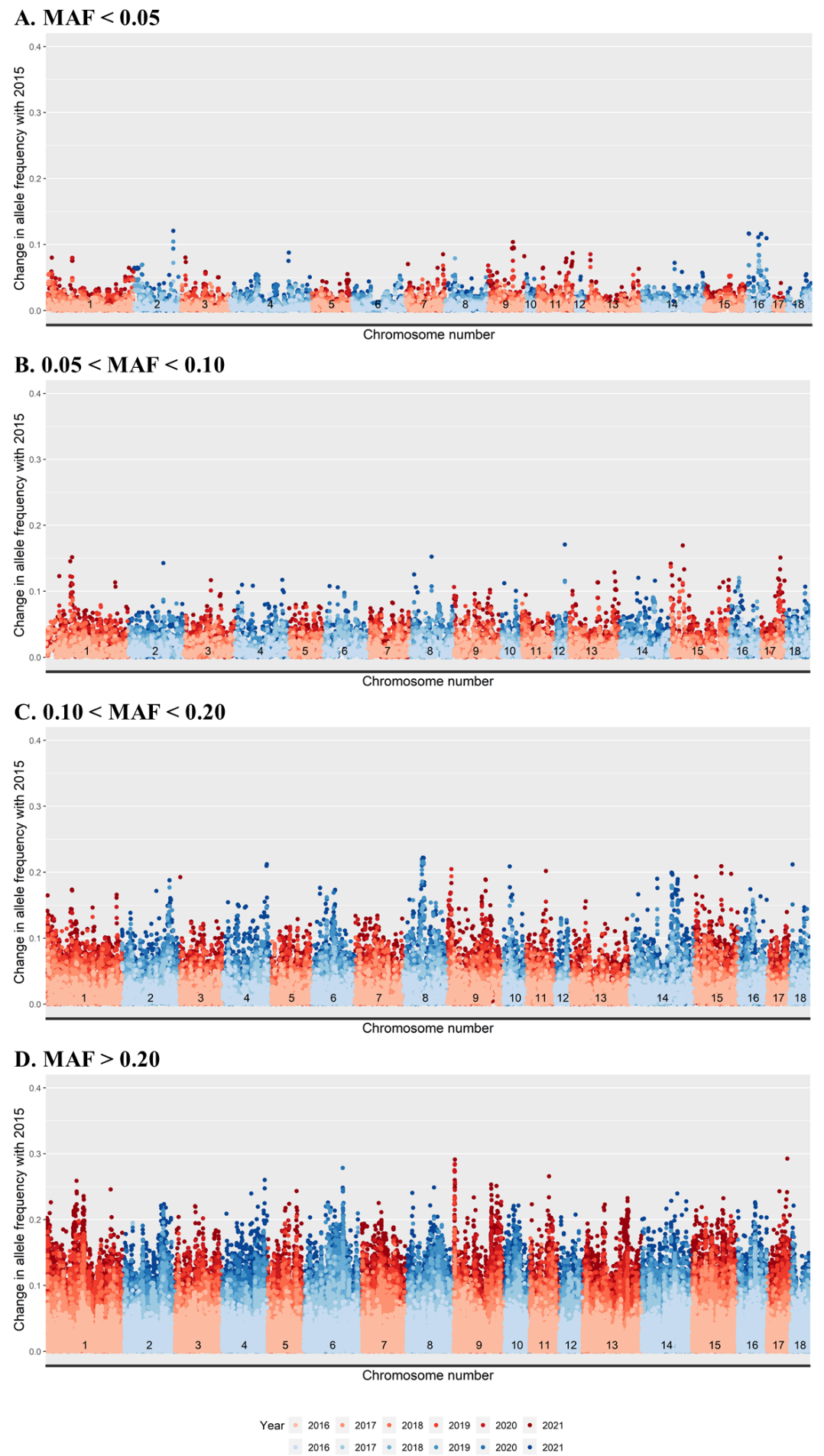


**Figure S1.1** Absolute allele frequency change compared to 2015 versus the genome location in line A, for different MAF classes in 2015.


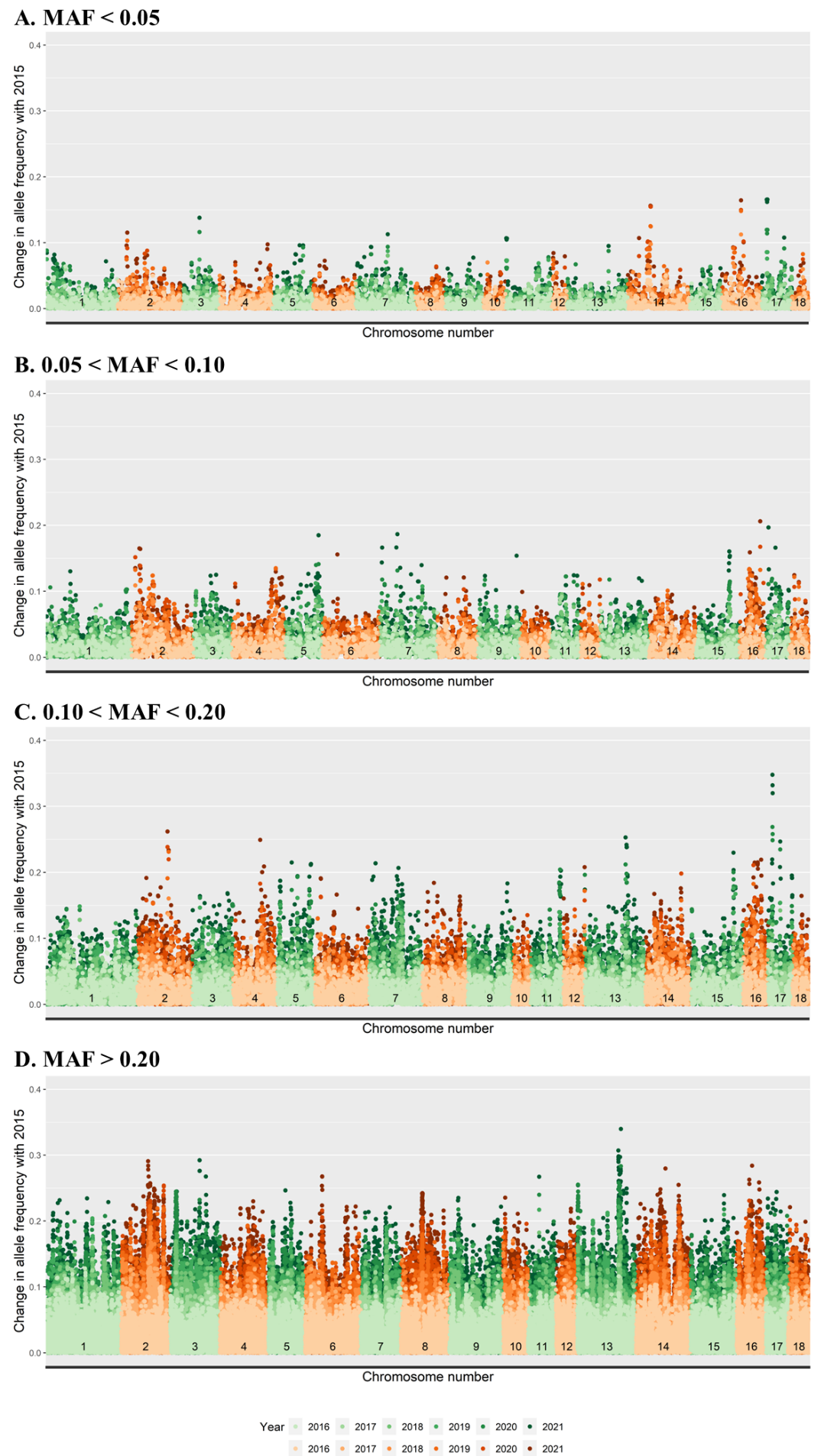


**Figure S1.2** Absolute allele frequency change compared to 2015 versus the genome location in line B, for different MAF classes in 2015.


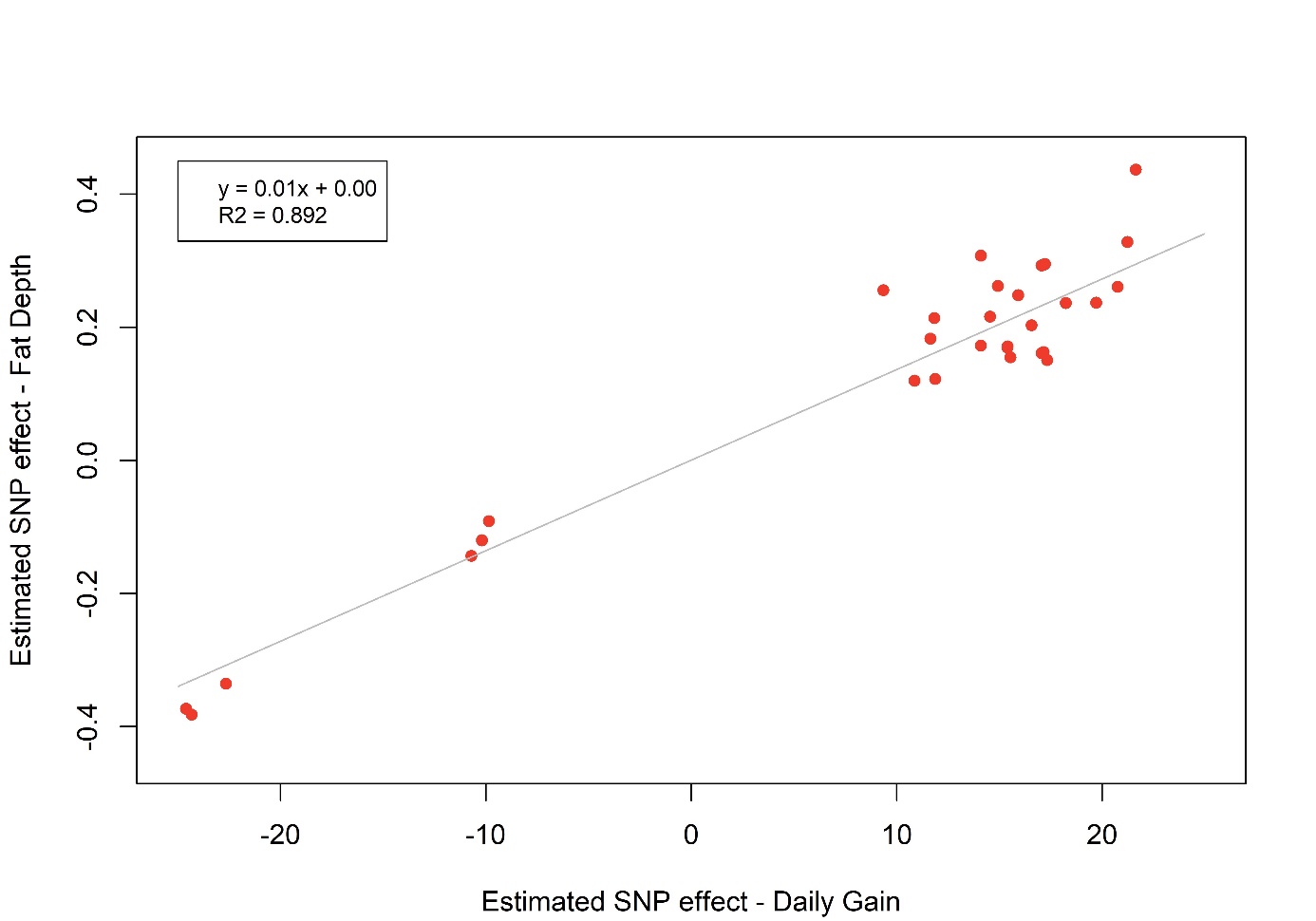


**Figure S1.3** Correlation of the estimated SNP effects of significant SNPs on SSC1 for daily gain and fat depth in Line A.


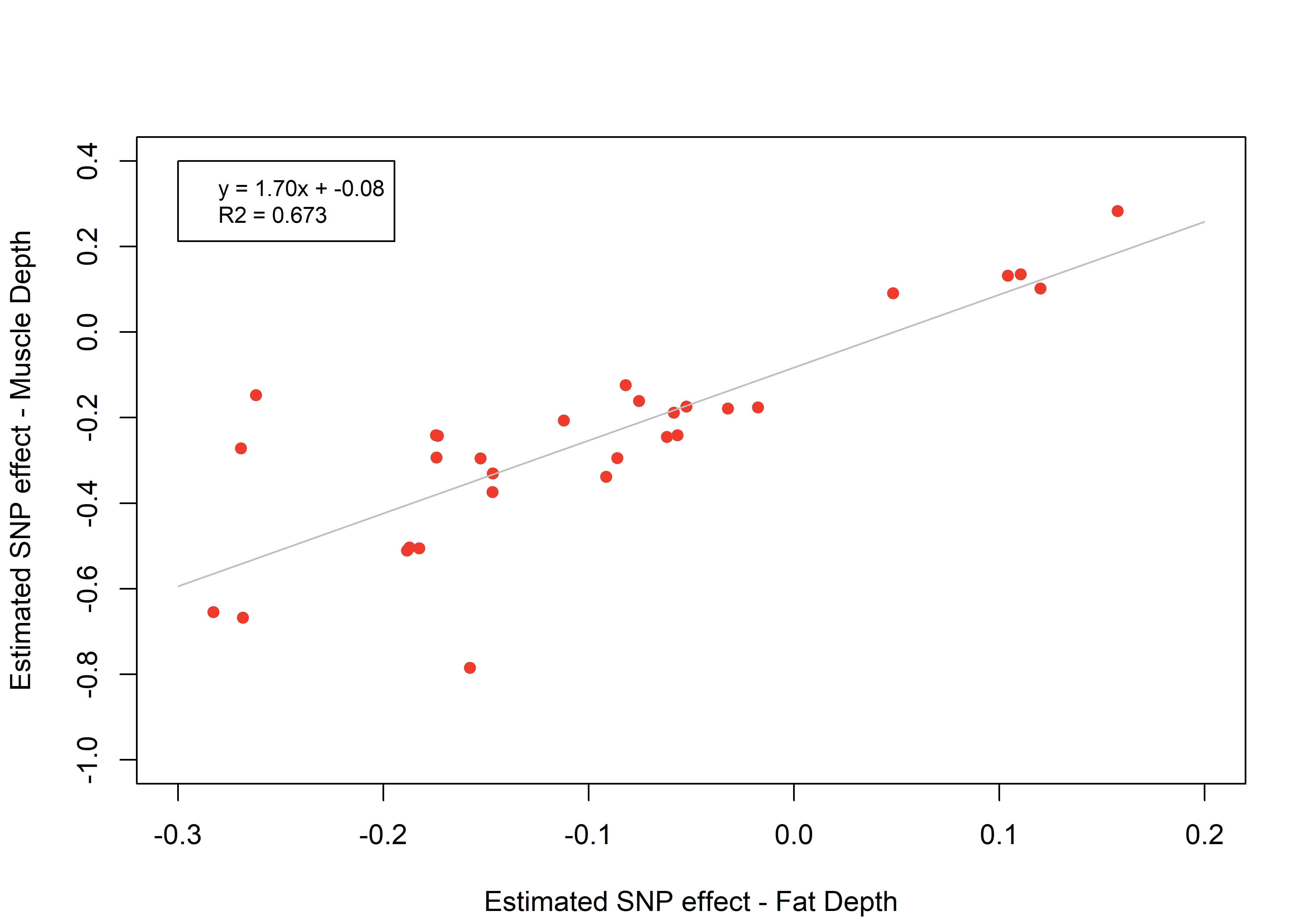


**Figure S1.4** Correlation of the estimated SNP effects of significant SNPs on SSC7 for fat depth and muscle depth in Line A.


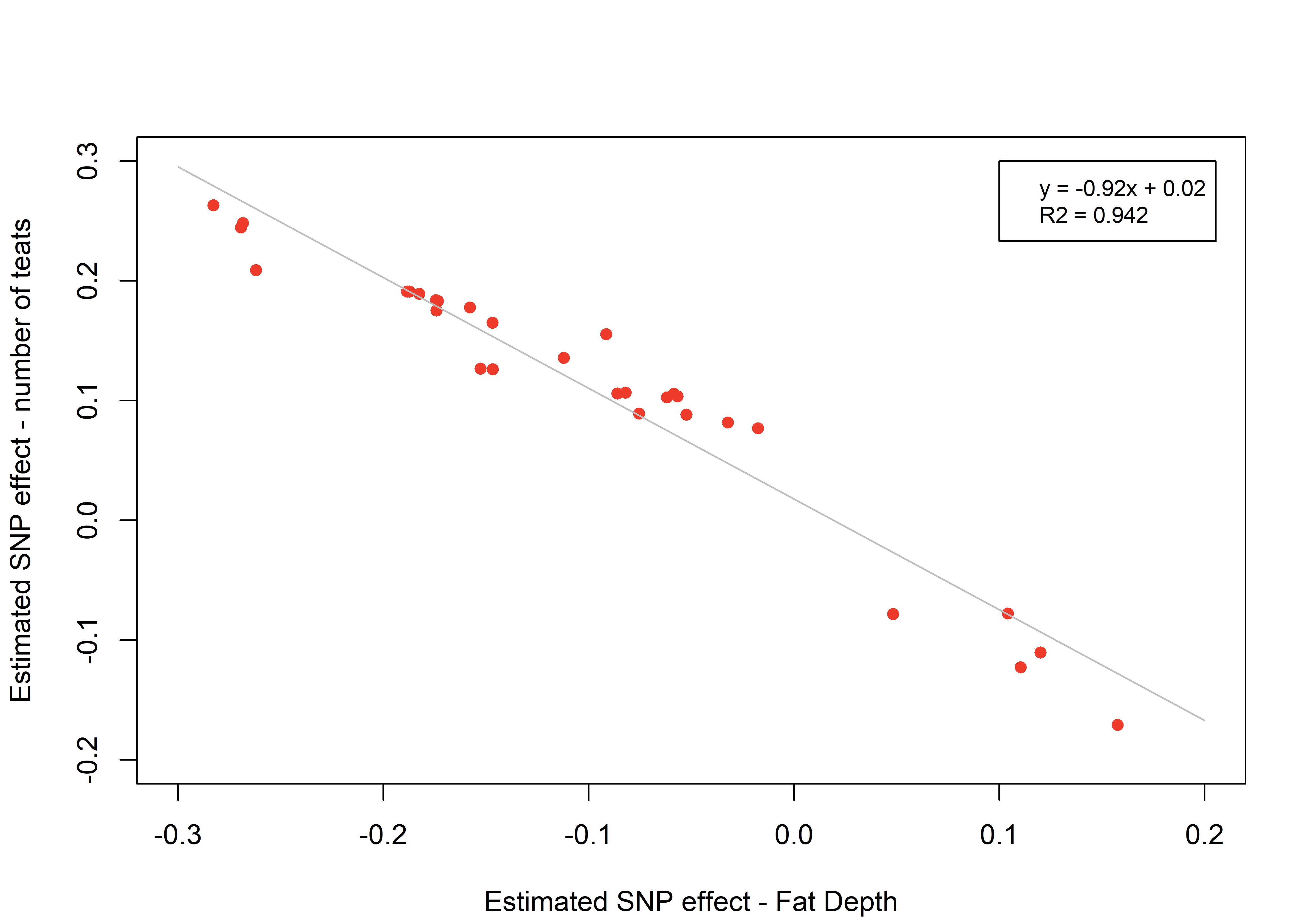


**Figure S1.5** Correlation of the estimated SNP effects of significant SNPs on SSC7 for fat depth and number of teats in Line A.


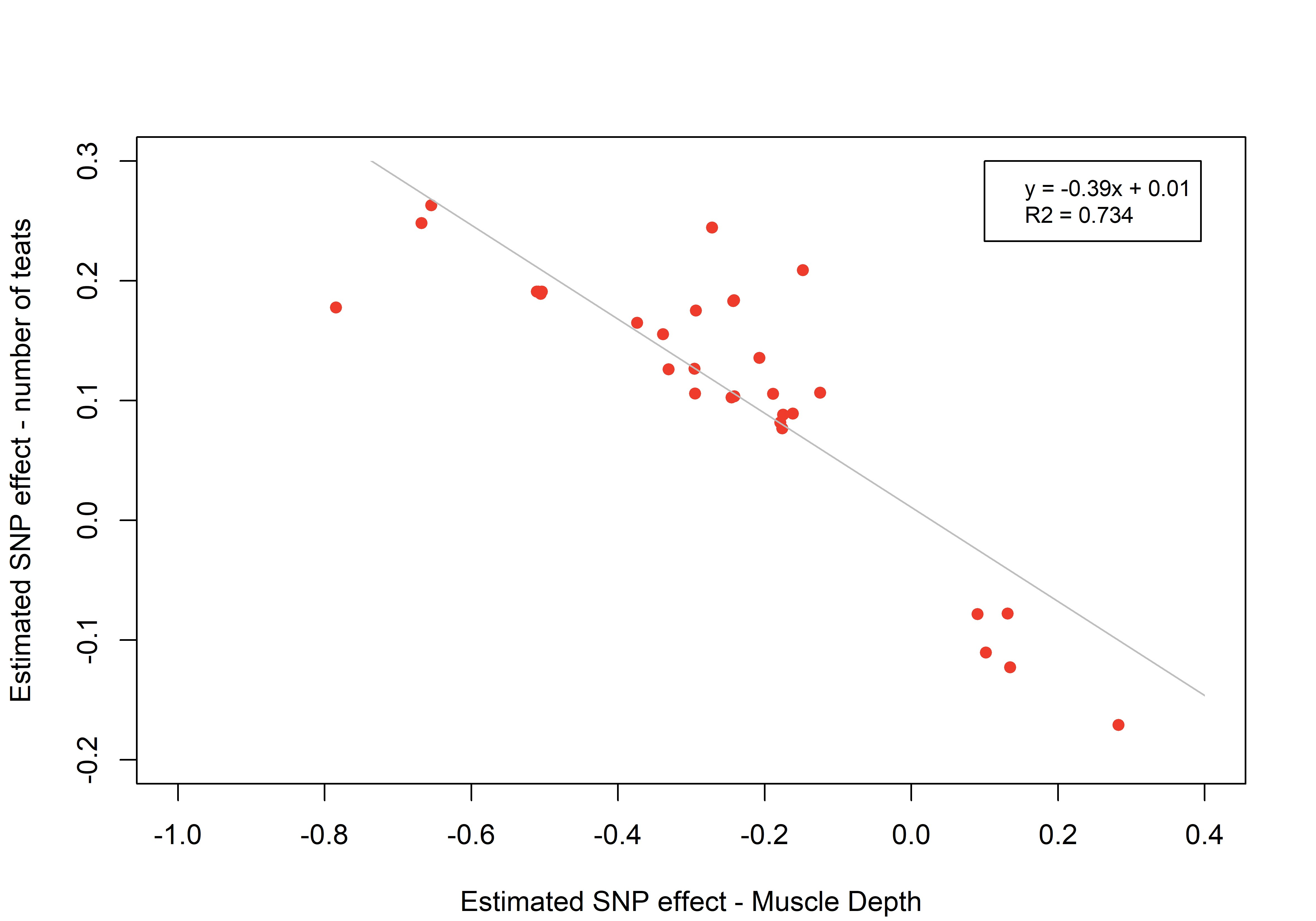


**Figure S1.6** Correlation of the estimated SNP effects of significant SNPs on SSC7 for muscle depth and number of teats in Line A.
