# Supplementary material for: Changes in allele frequencies and genetic architecture due to selection in two pig populations
Source: Genet Sel Evol. 2024 Dec 17;56:76. doi: 10.1186/s12711-024-00941-3 (PMC11650847; doi:10.1186/s12711-024-00941-3)
Supplement: Supplementary file 2 — Additional file 2. Quantile–Quantile (QQ) plots of the GWAS analyses. Eighteen figures with the QQ plots for the different analyses. [file 12711_2024_941_MOESM2_ESM.docx]

**Additional file 2: Quantile-Quantile (QQ) plots of GWAS analyses**


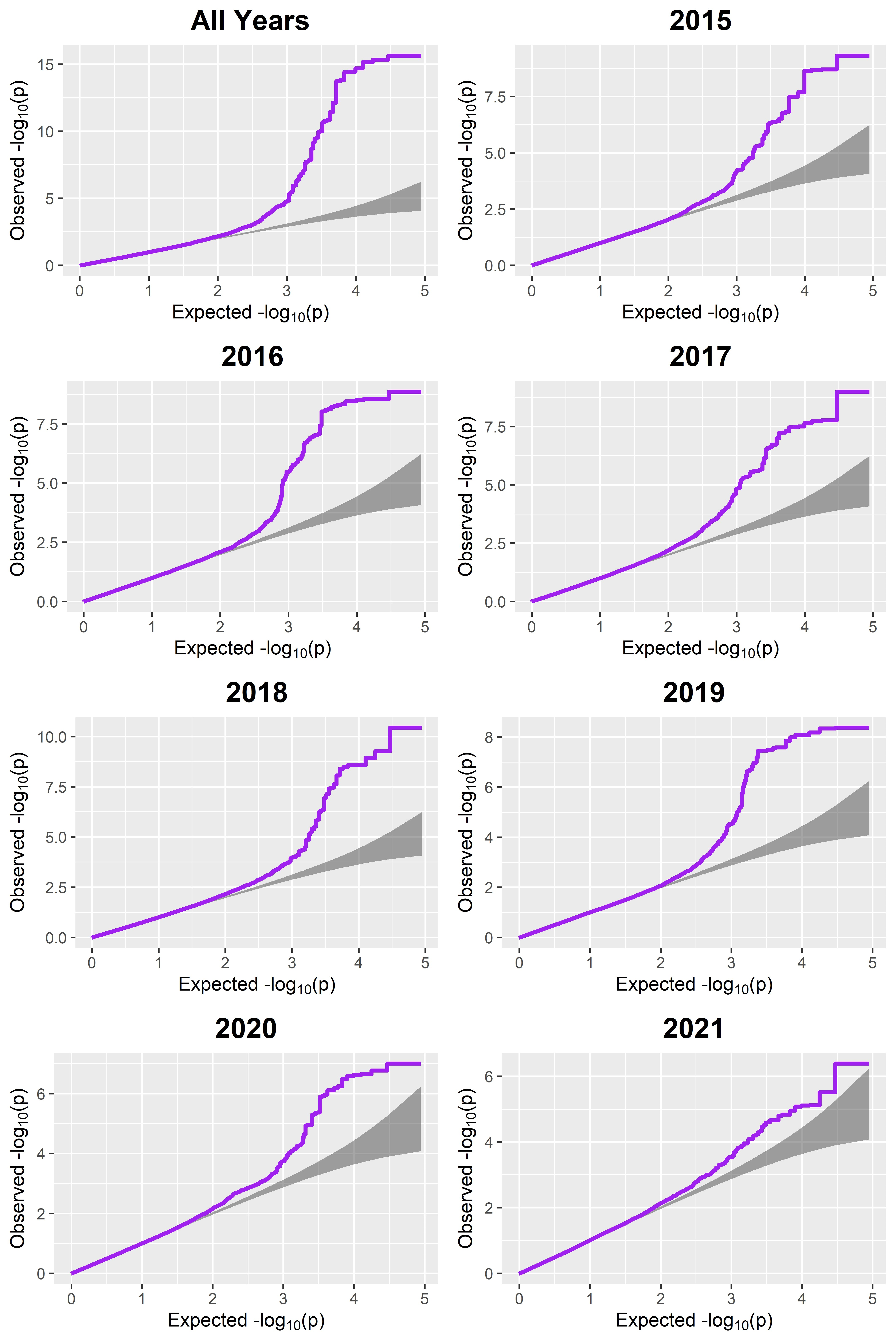


**Figure S2.1** QQ plots for daily gain in line A for the different years. The grey area indicates the 95% confidence interval under the null hypothesis.


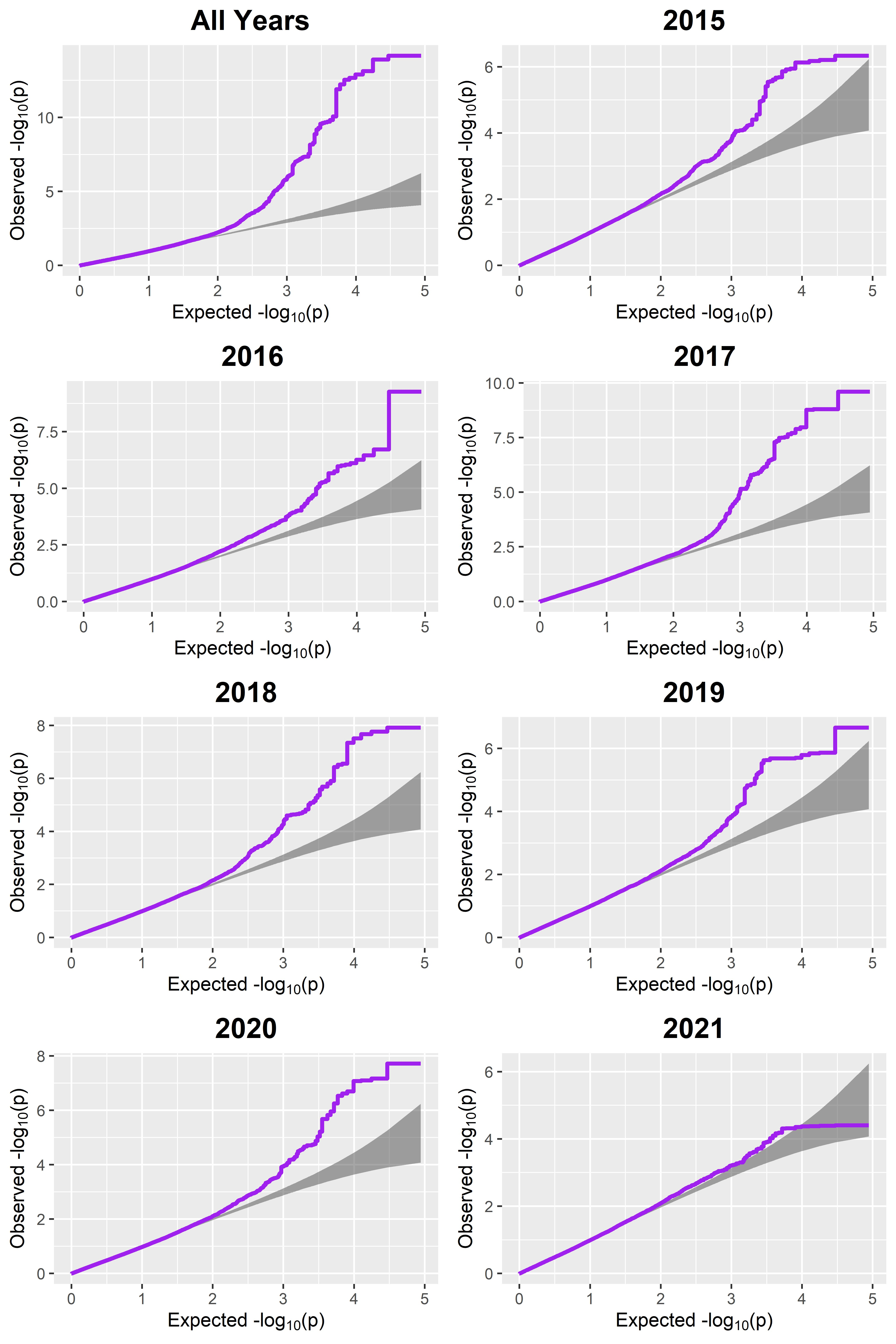


**Figure S2.2** QQ plots for fat depth in line A for the different years. The grey area indicates the 95% confidence interval under the null hypothesis.


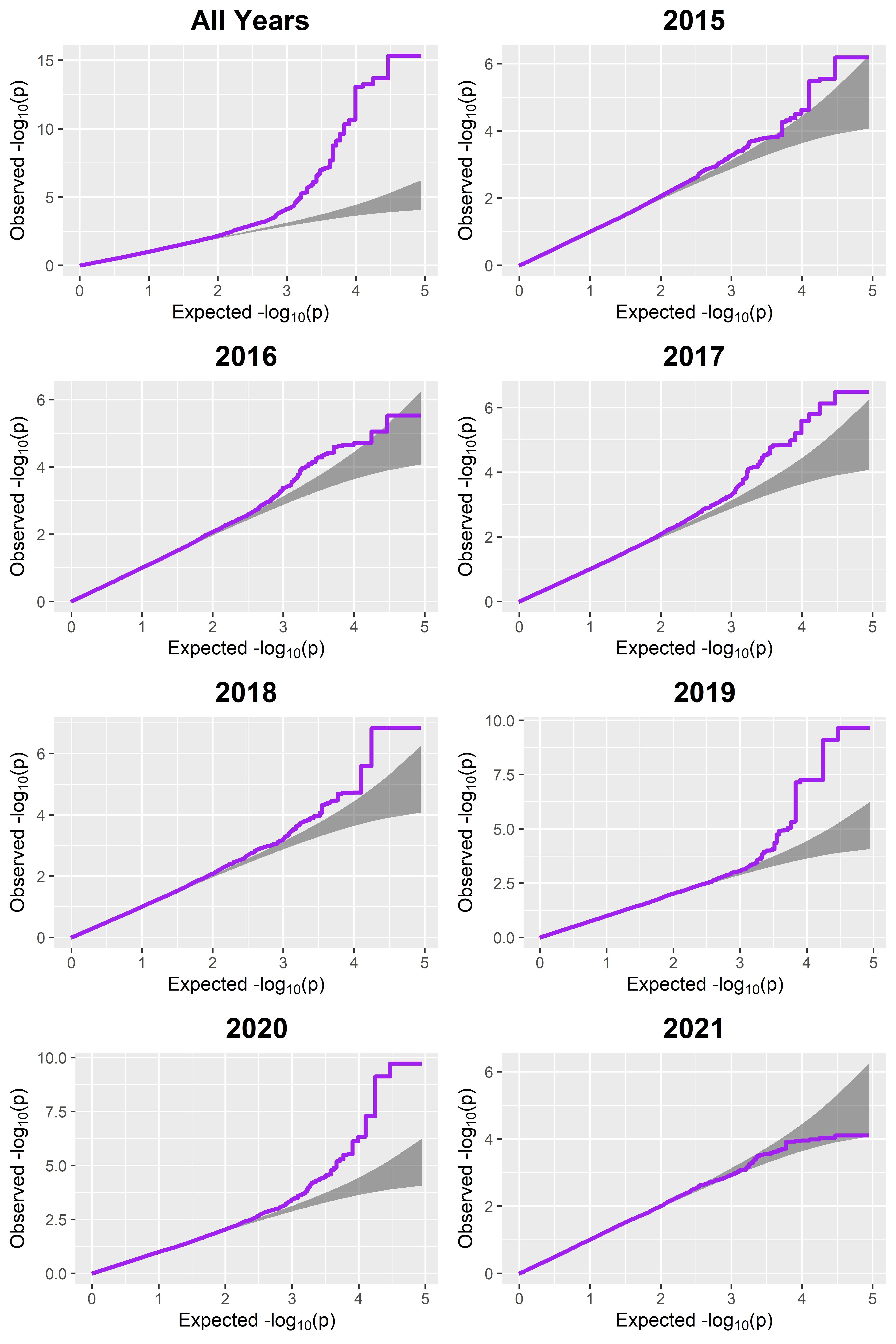


**Figure S2.3** QQ plots for muscle depth in line A for the different years. The grey area indicates the 95% confidence interval under the null hypothesis.


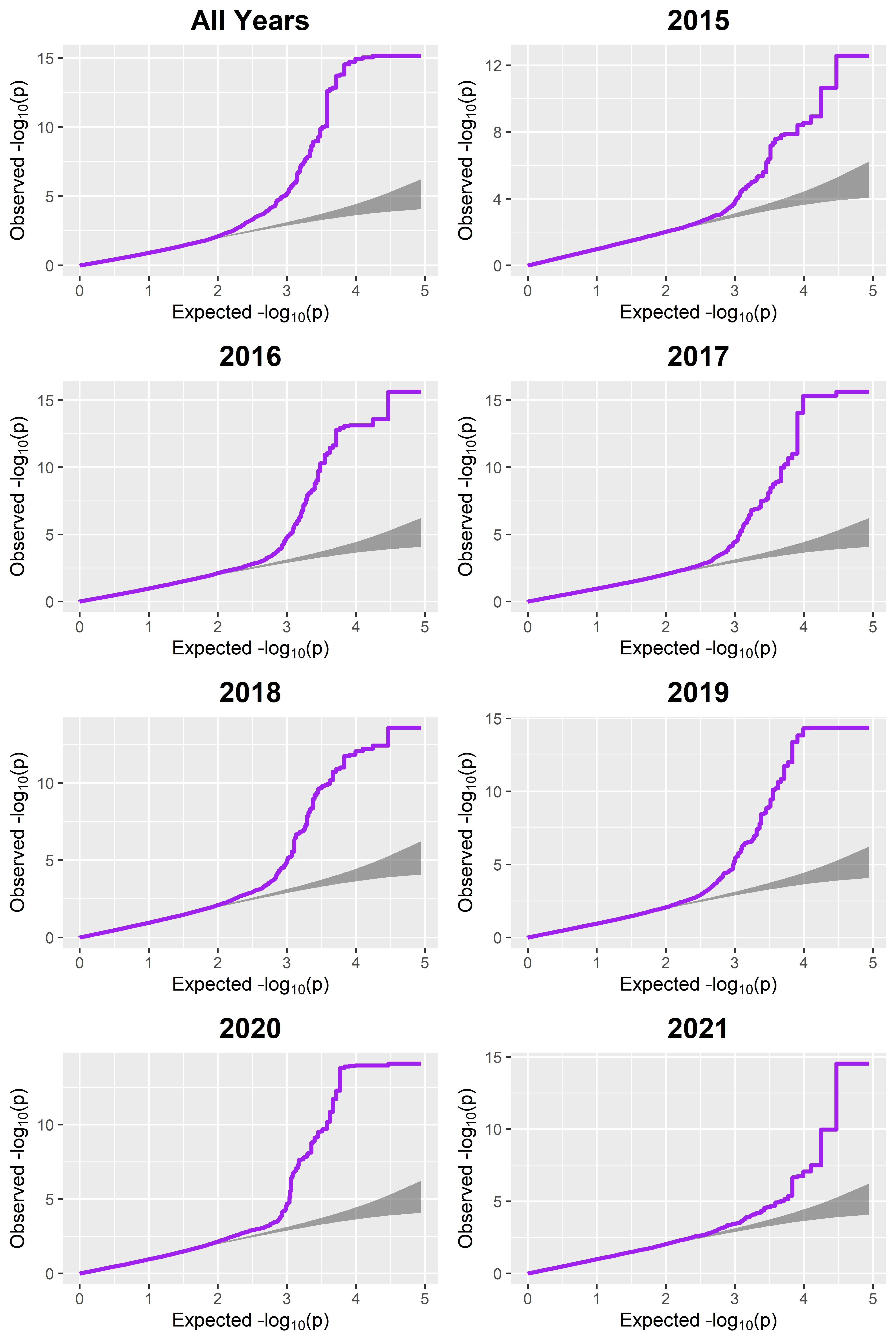


**Figure S2.4** QQ plots for number of teats in line A for the different years. The grey area indicates the 95% confidence interval under the null hypothesis.


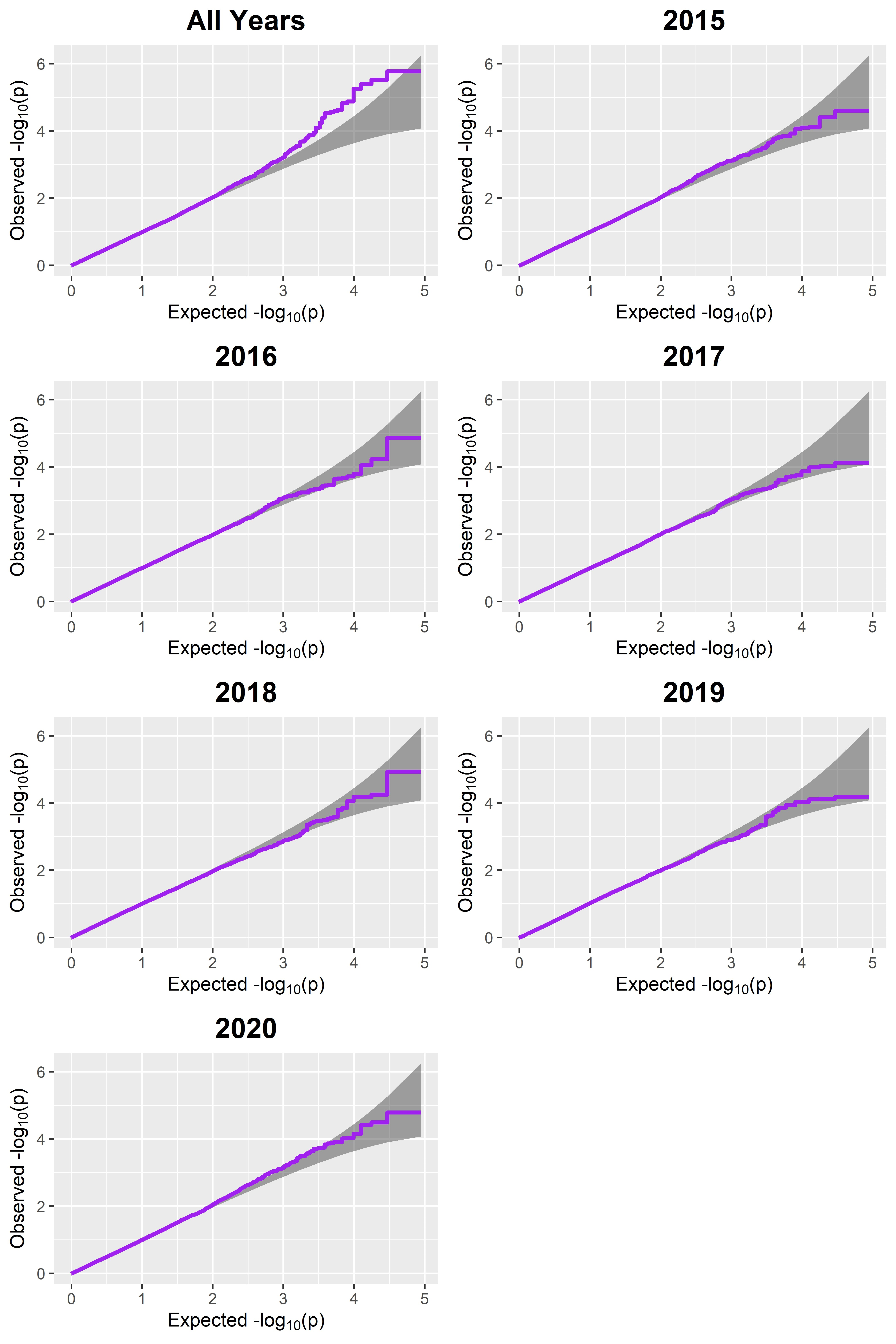


**Figure S2.5** QQ plots for total number born first parity in line A for the different years. The grey area indicates the 95% confidence interval under the null hypothesis.


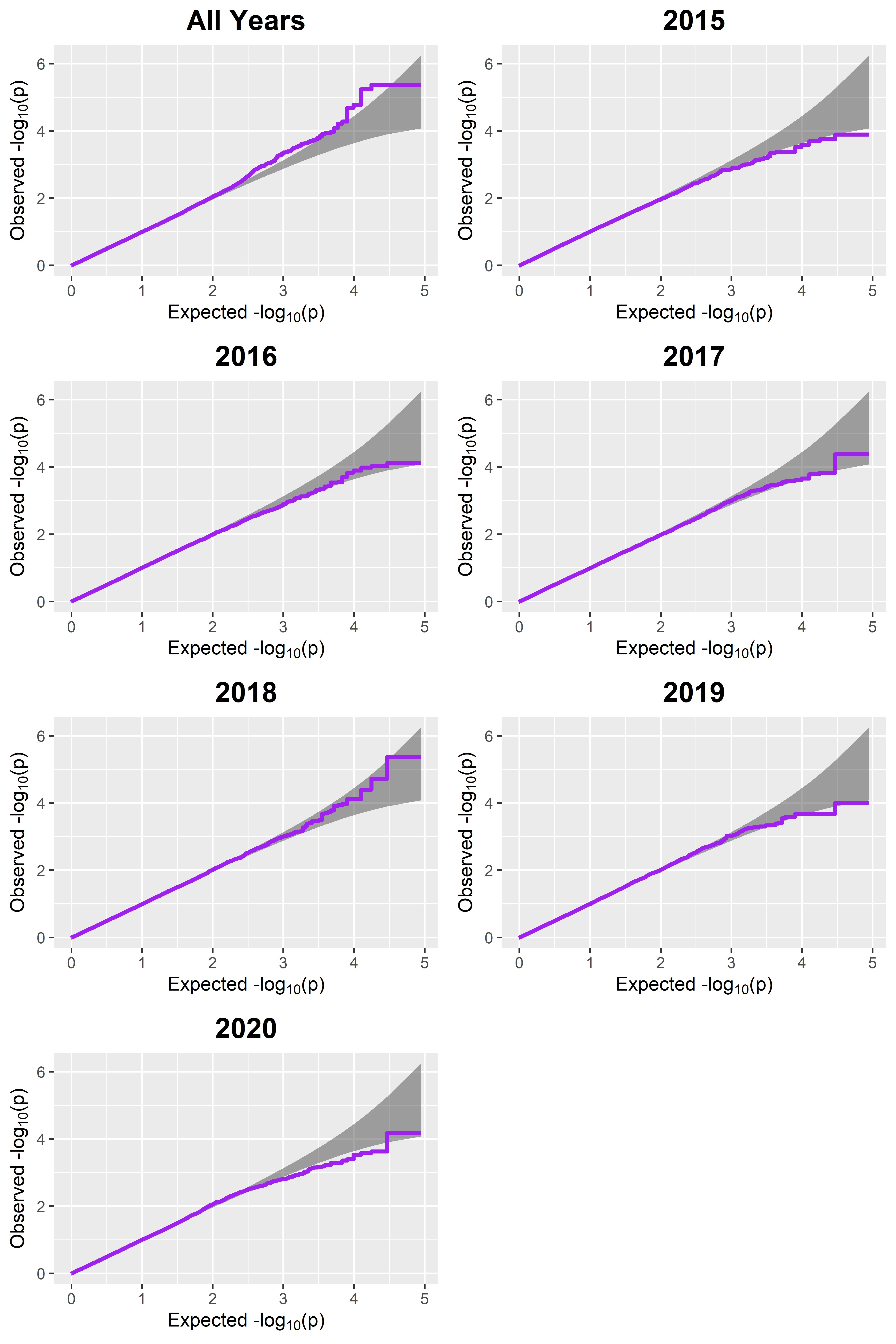


**Figure S2.6** QQ plots for average birth weight first litter in line A for the different years. The grey area indicates the 95% confidence interval under the null hypothesis.


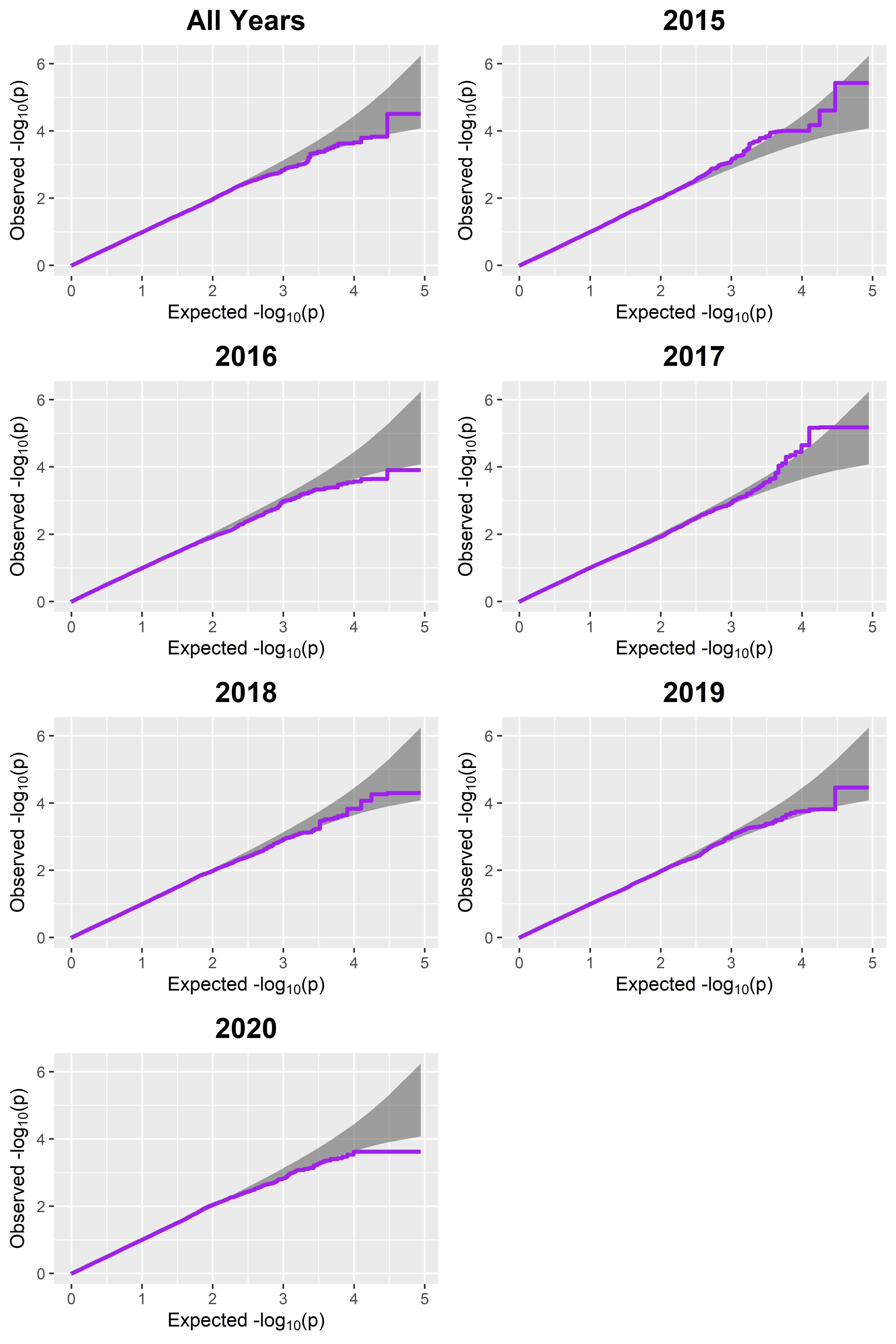


**Figure S2.7** QQ plots for CV of birth weight first litter in line A for the different years. The grey area indicates the 95% confidence interval under the null hypothesis.


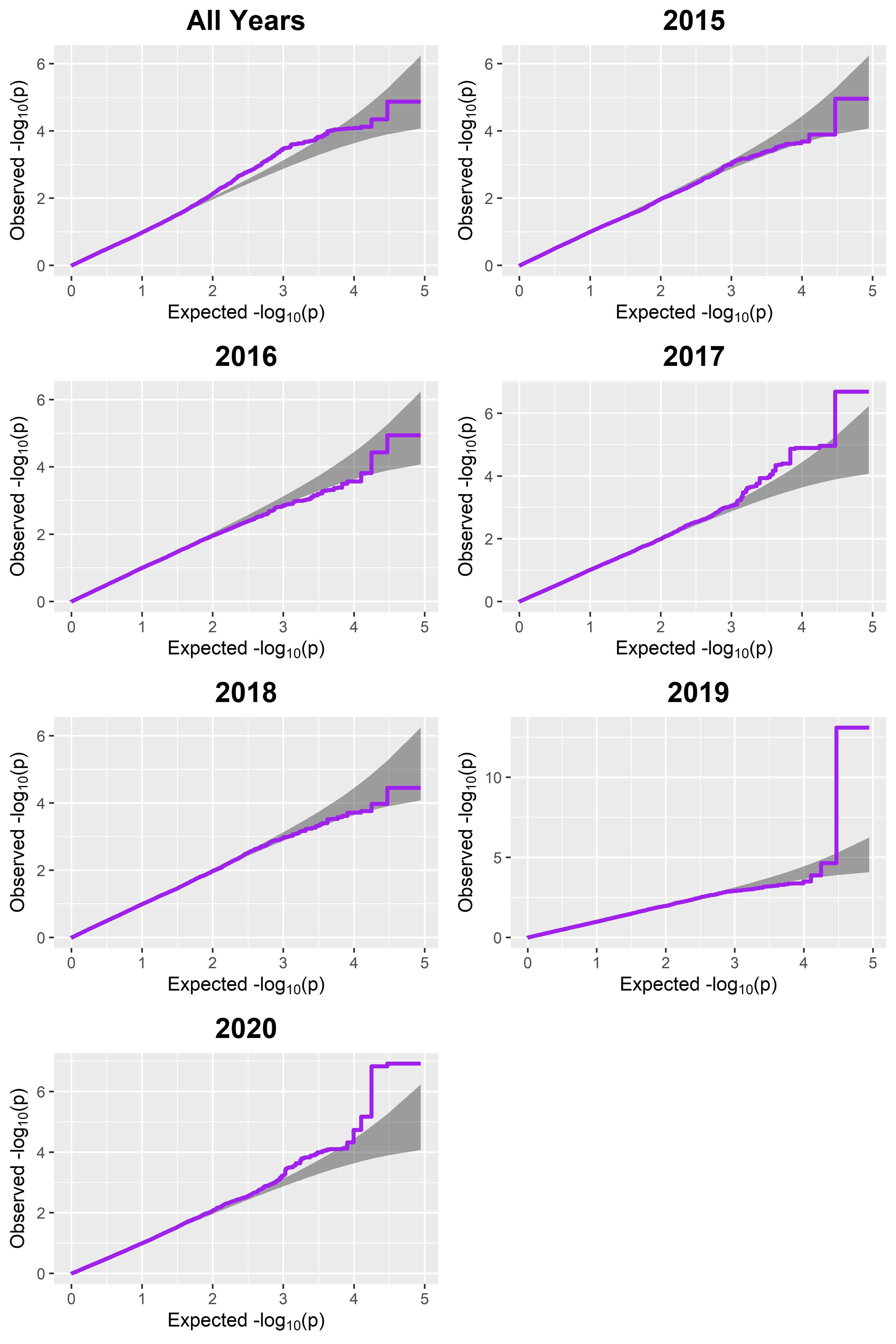


**Figure S2.8** QQ plots for number of small piglets in line A for the different years. The grey area indicates the 95% confidence interval under the null hypothesis.


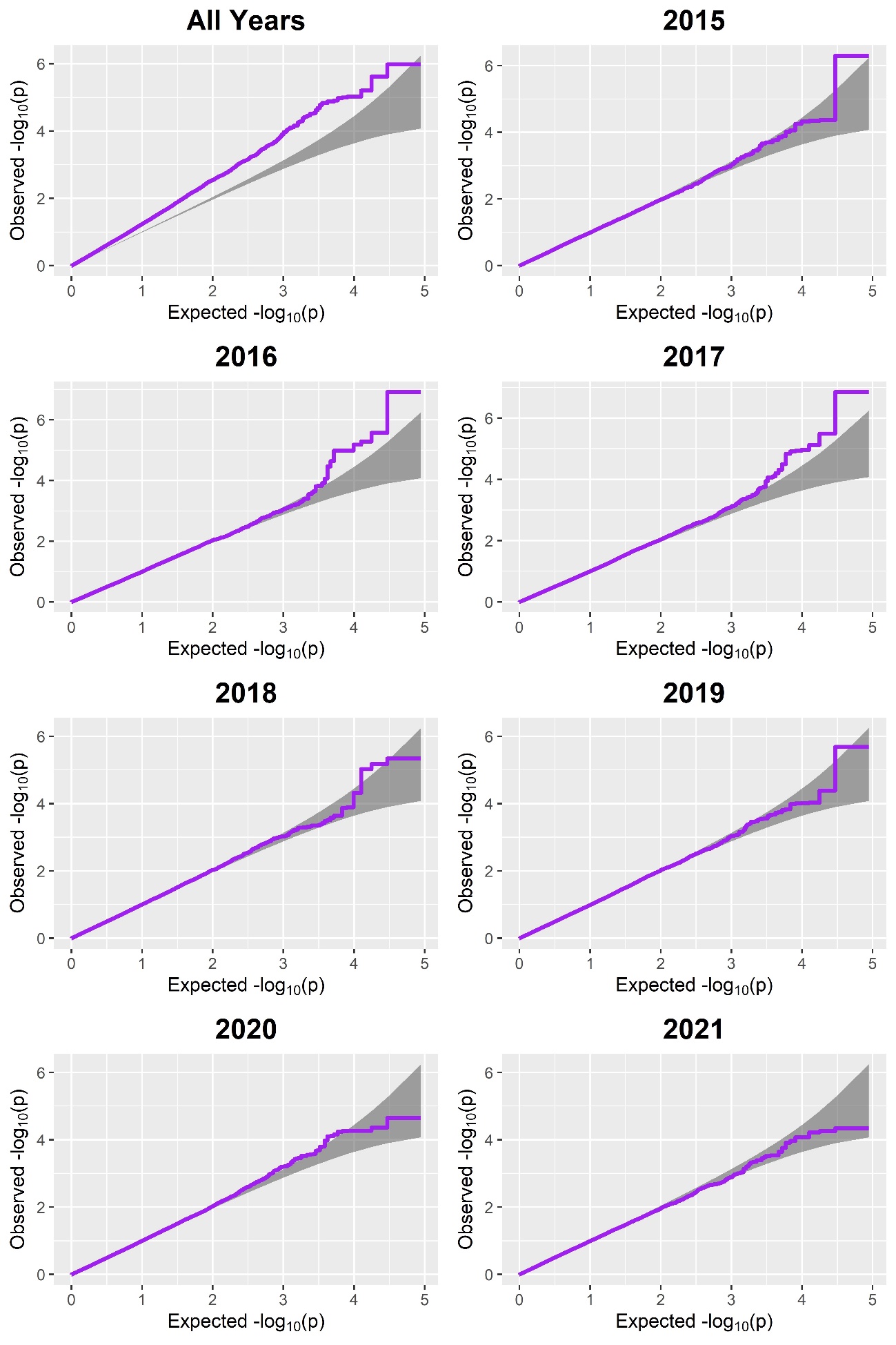


**Figure S2.9** QQ plots for the index in line A for the different years. The grey area indicates the 95% confidence interval under the null hypothesis.


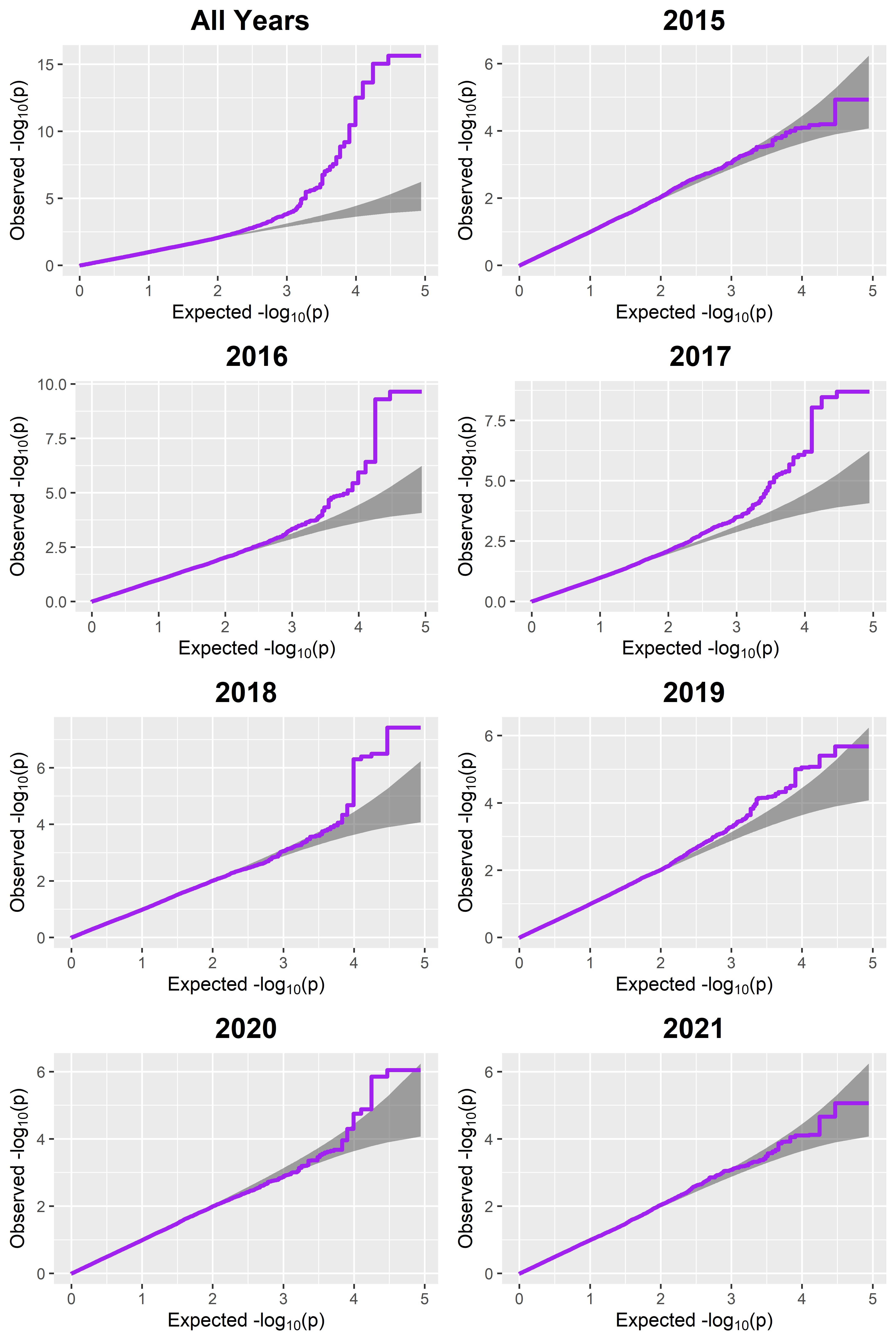


**Figure S2.10** QQ plots for daily gain in line B for the different years. The grey area indicates the 95% confidence interval under the null hypothesis.


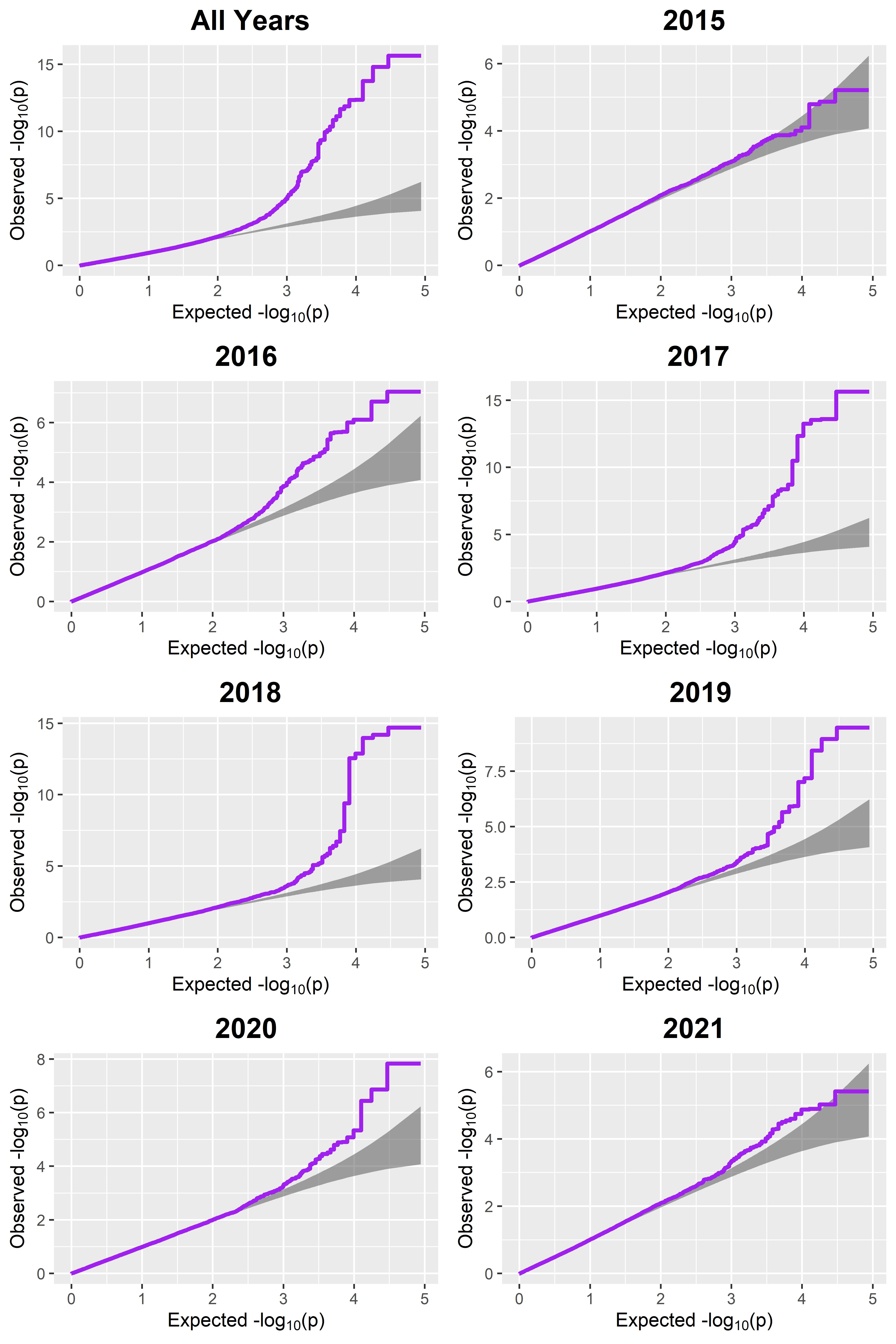


**Figure S2.11** QQ plots for fat depth in line B for the different years. The grey area indicates the 95% confidence interval under the null hypothesis.


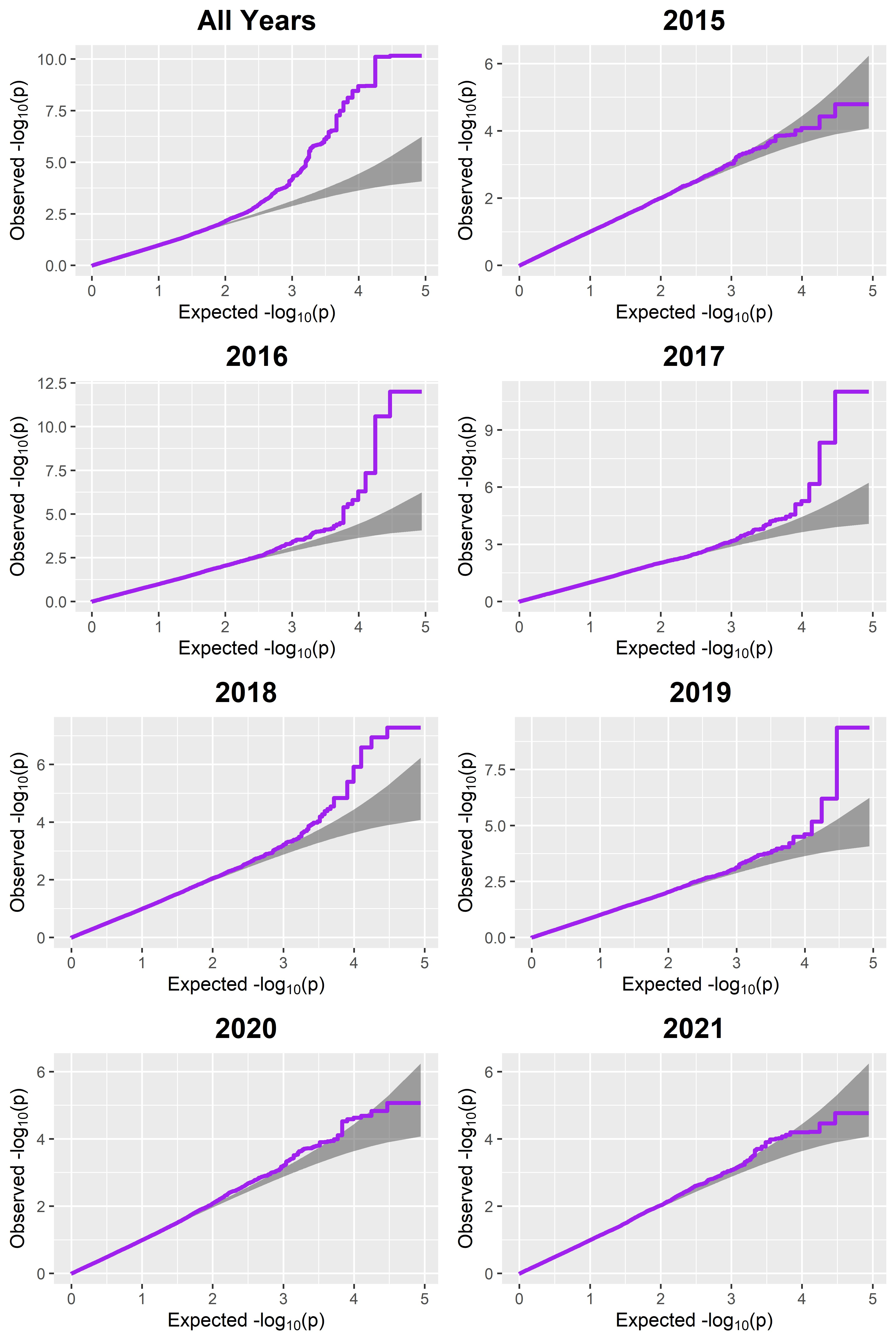


**Figure S2.12** QQ plots for muscle depth in line B for the different years. The grey area indicates the 95% confidence interval under the null hypothesis.


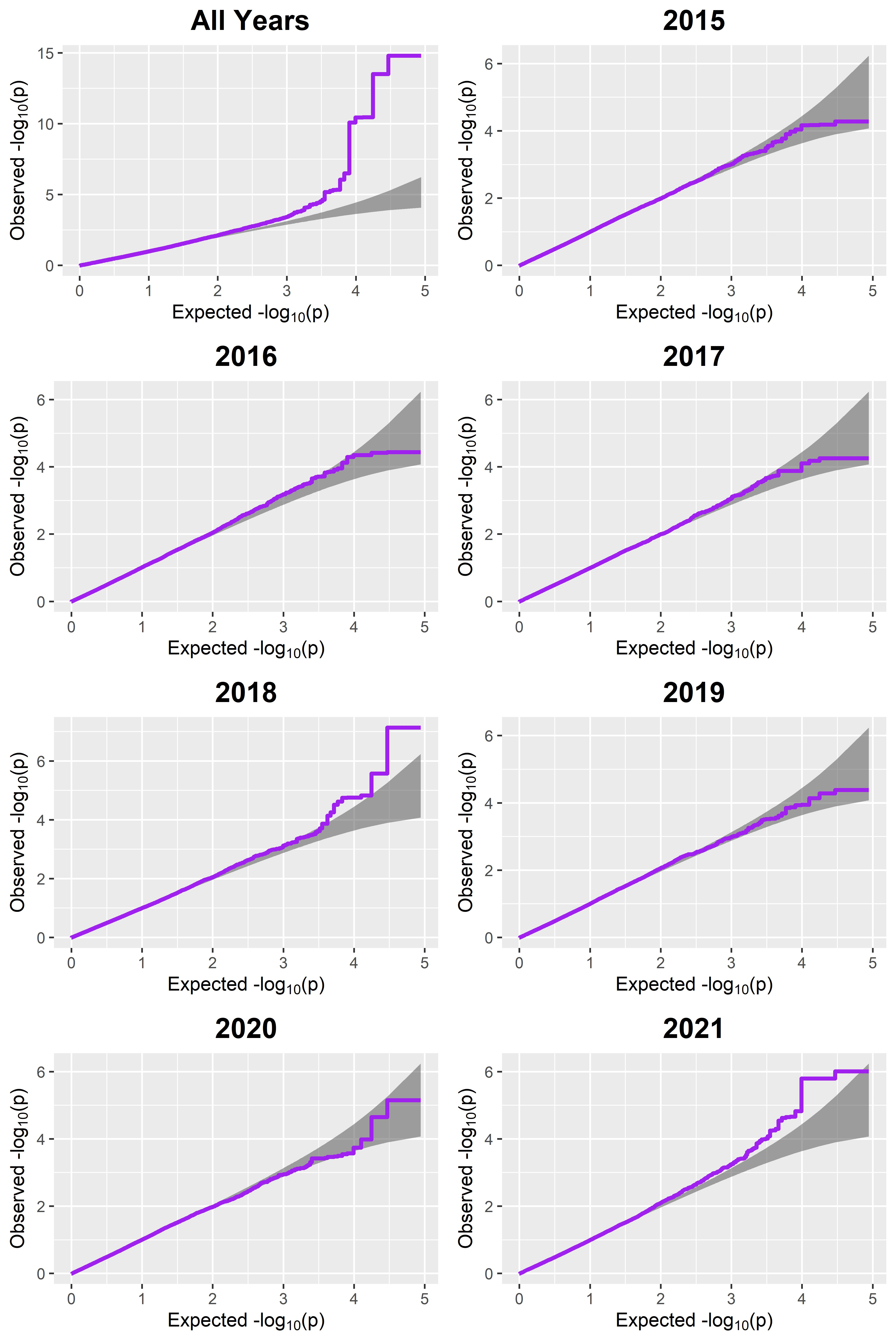


**Figure S2.13** QQ plots for number of teats in line B for the different years. The grey area indicates the 95% confidence interval under the null hypothesis.


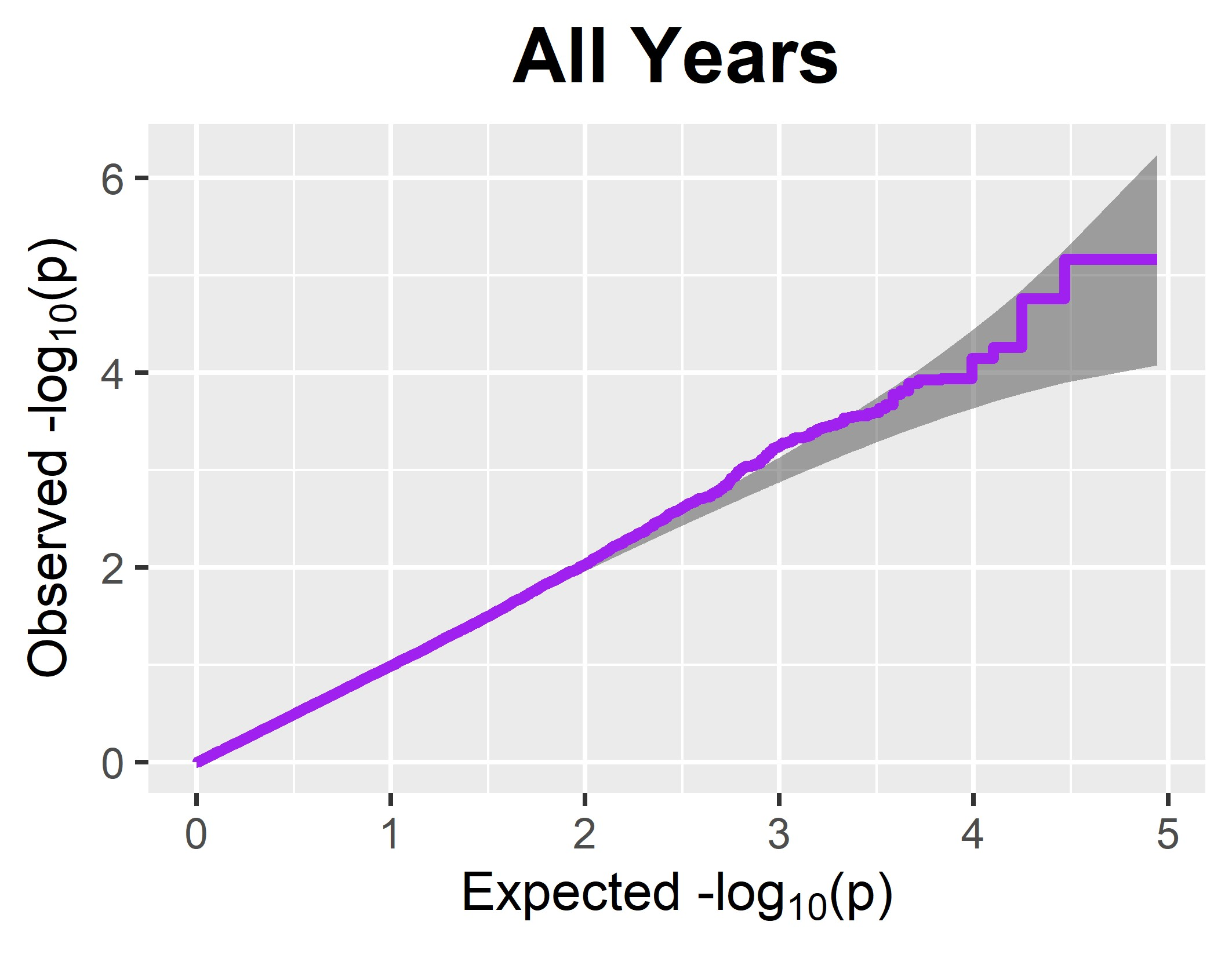


**Figure S2.14** QQ plot for total number born first parity in line B. The grey area indicates the 95% confidence interval under the null hypothesis.


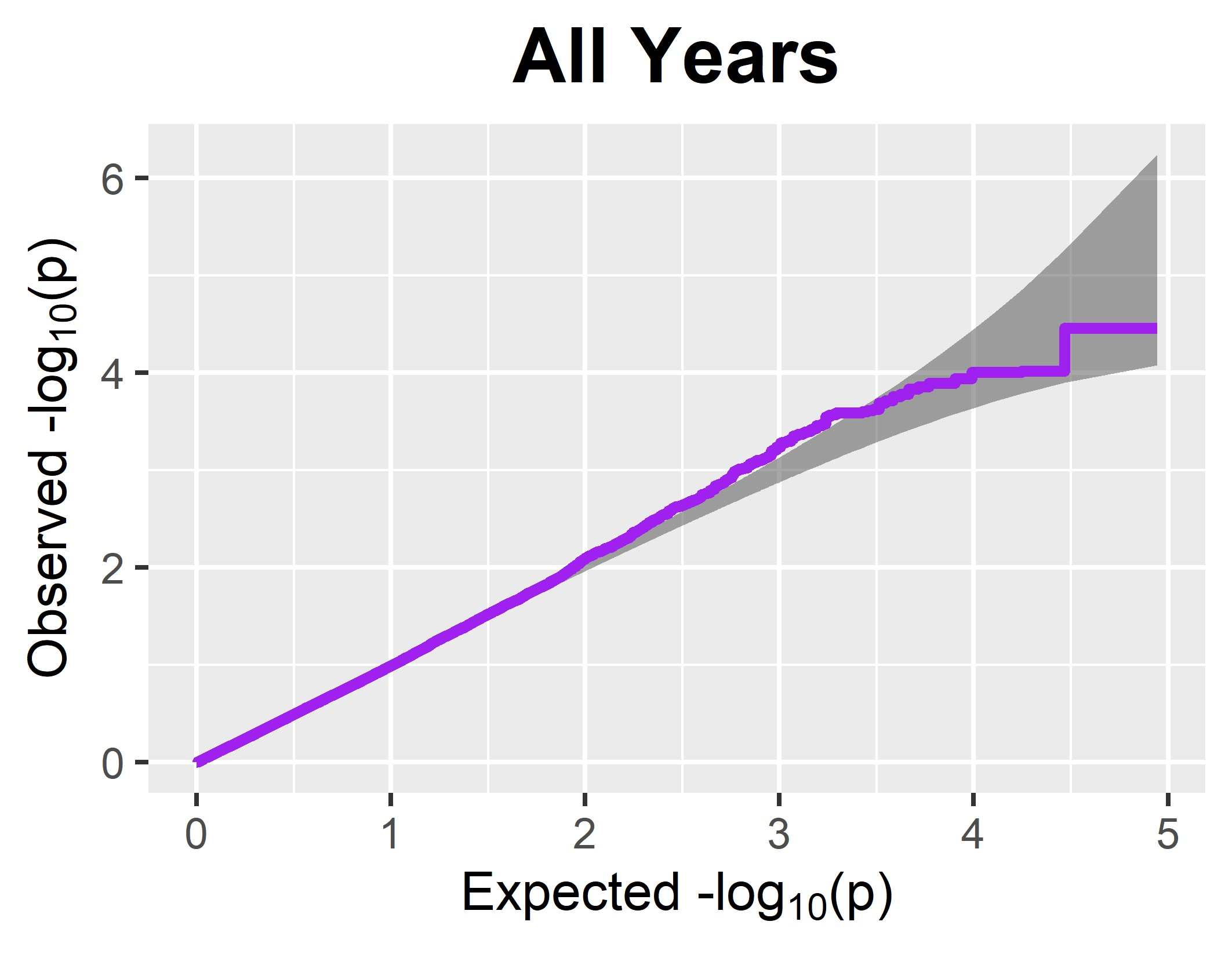


**Figure S2.15** QQ plot for average birth weight first litter in line B. The grey area indicates the 95% confidence interval under the null hypothesis.


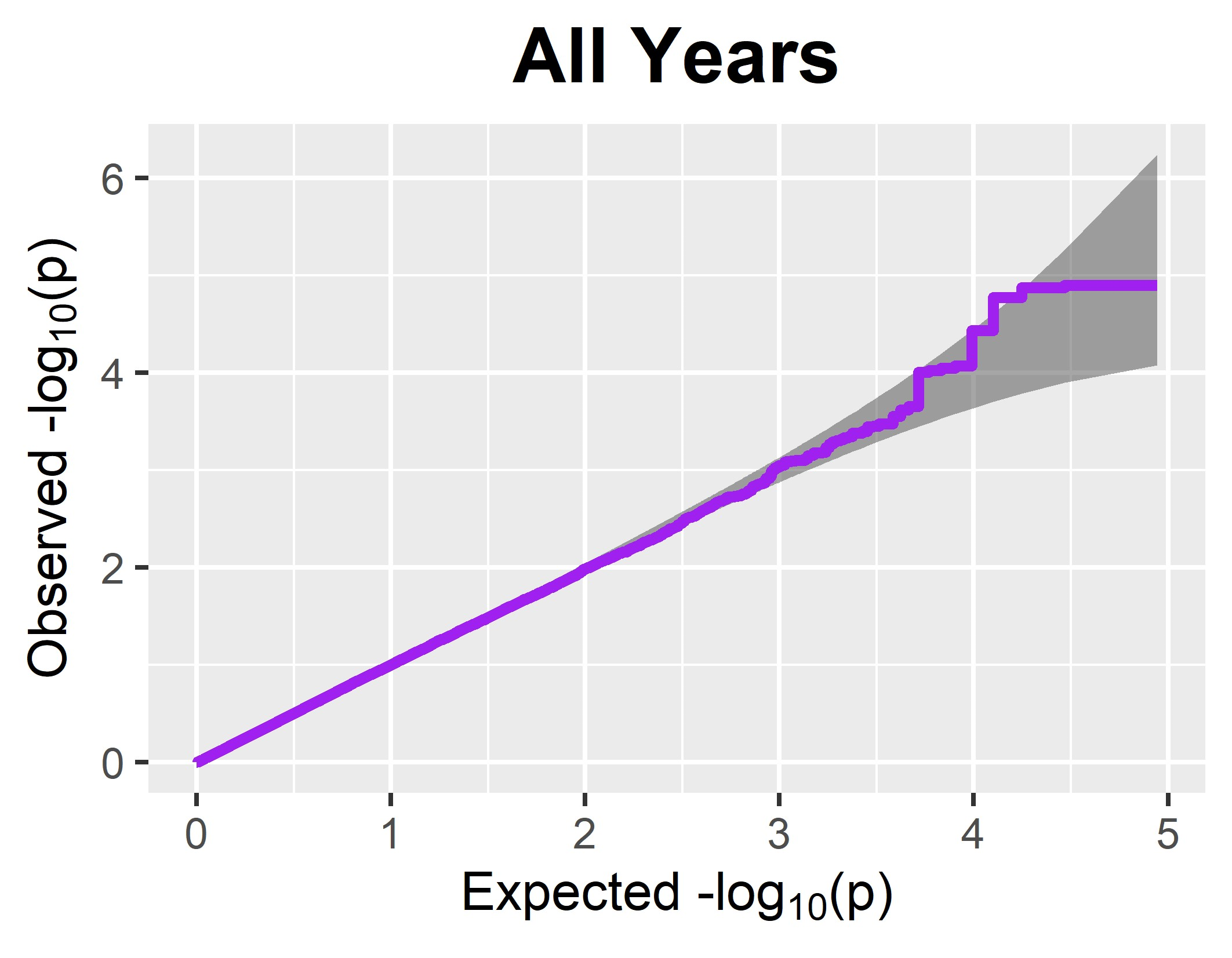


**Figure S2.16** QQ plot for CV of birth weight first litter in line B. The grey area indicates the 95% confidence interval under the null hypothesis.


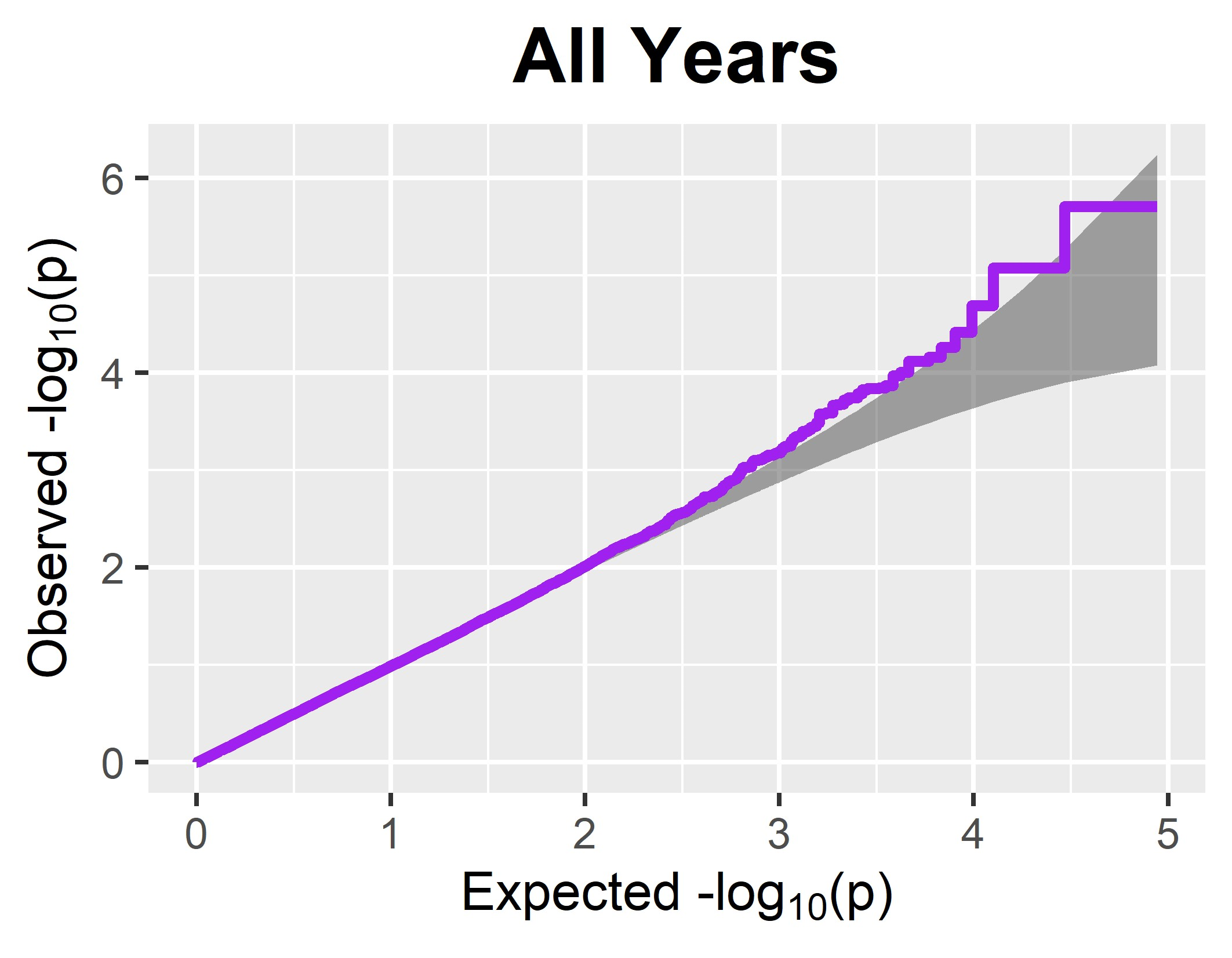


**Figure S2.17** QQ plot for number of small piglets in line B. The grey area indicates the 95% confidence interval under the null hypothesis.

a
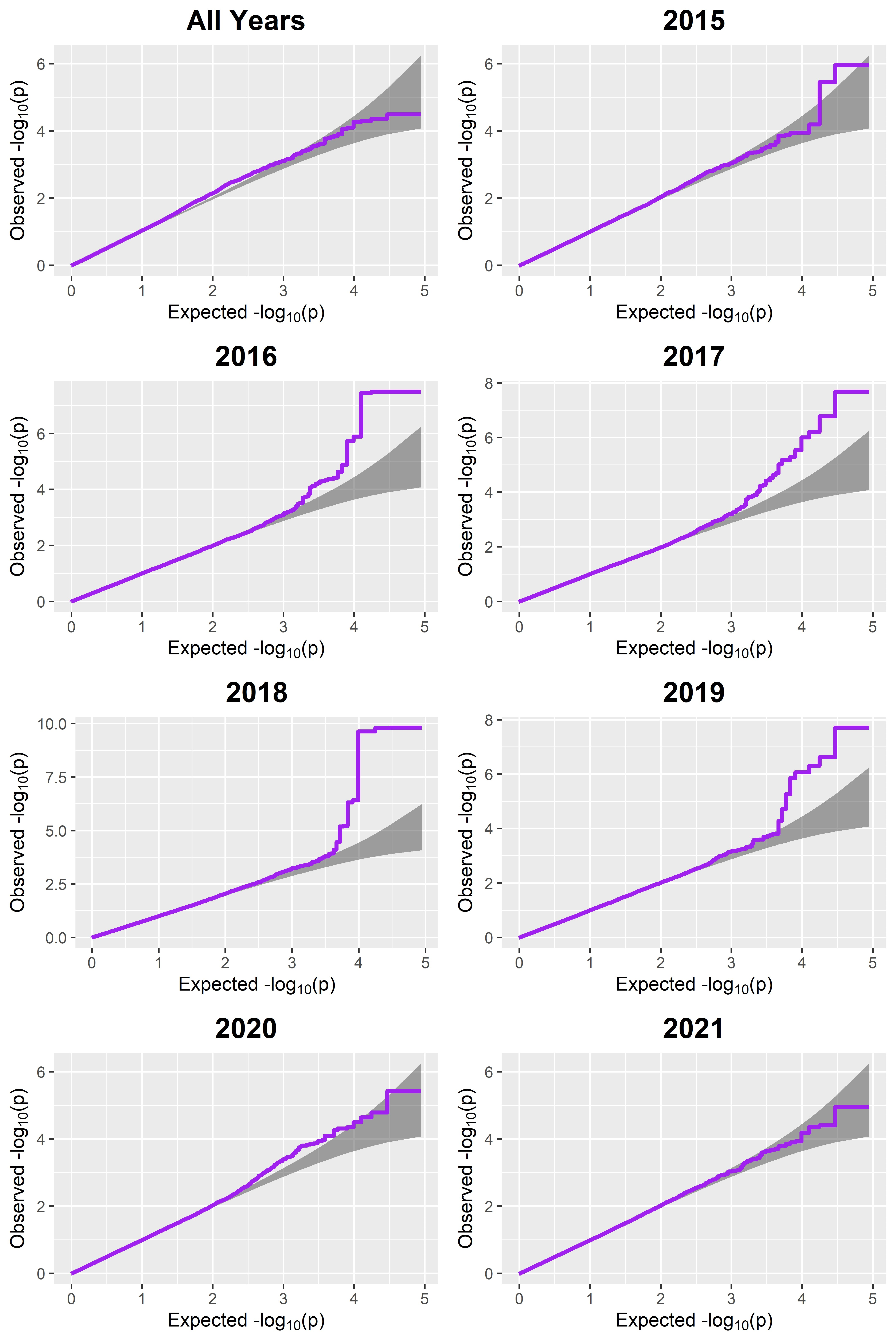


**Figure S2.18** QQ plots for the index in line B for the different years. The grey area indicates the 95% confidence interval under the null hypothesis.
