# Supplementary material for: Changes in allele frequencies and genetic architecture due to selection in two pig populations
Source: Genet Sel Evol. 2024 Dec 17;56:76. doi: 10.1186/s12711-024-00941-3 (PMC11650847; doi:10.1186/s12711-024-00941-3)
Supplement: Supplementary file 3 — Additional file 3. Correlation allele frequency change and GWAS results. Fourteen figures describing the correlation between allele frequency change and GWAS results (estimated effect and significance level). [file 12711_2024_941_MOESM3_ESM.docx]

**Additional file 3: Correlation allele frequency change and GWAS results**


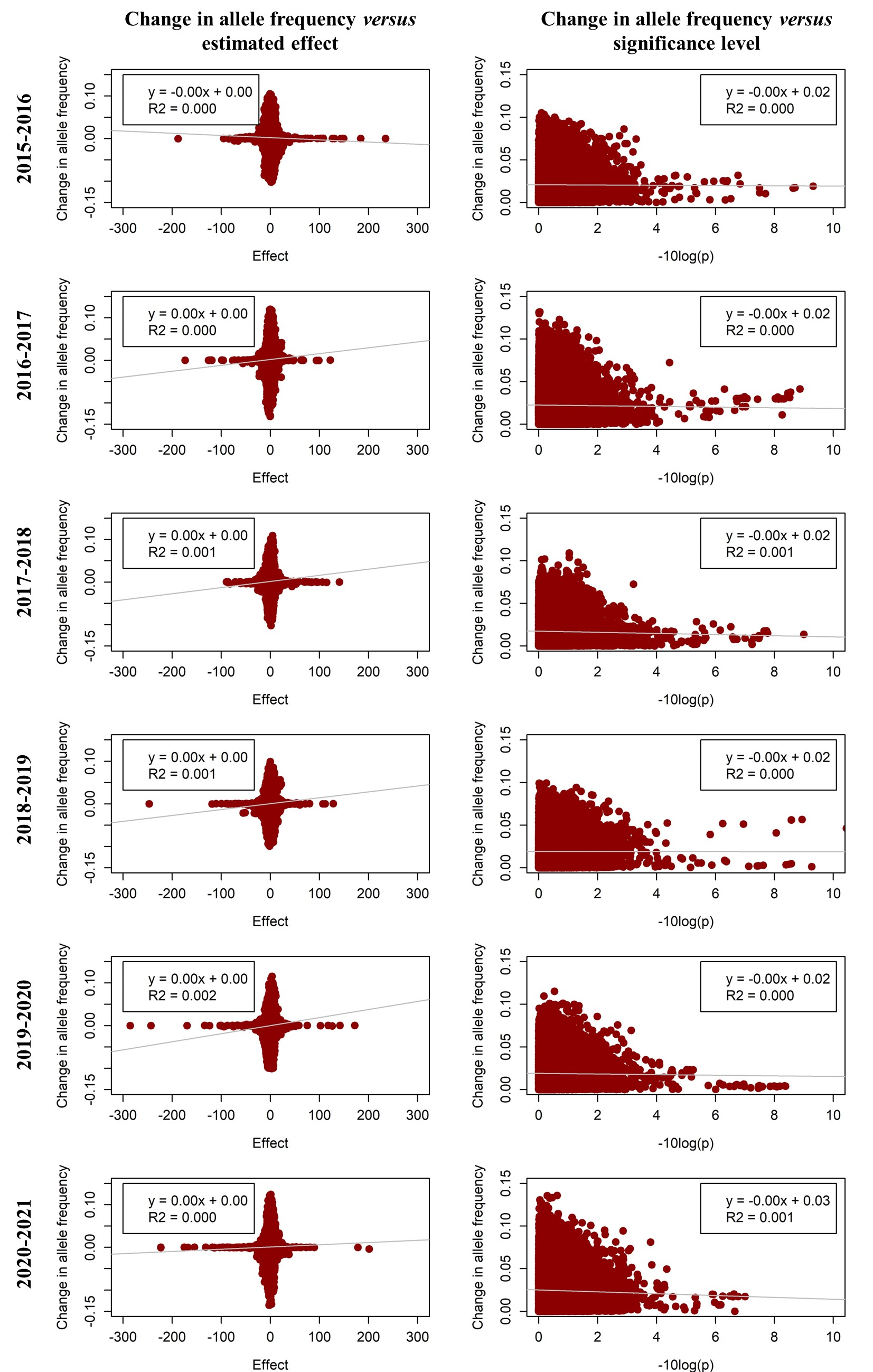


**Figure S3.1** Change in allele frequency versus estimated effect and significance level for daily gain in each year in line A. Estimated effects are from a GWAS per year, and the change in allele frequency is the change towards the next year, with the absolute value of allele frequency change for the significance level.


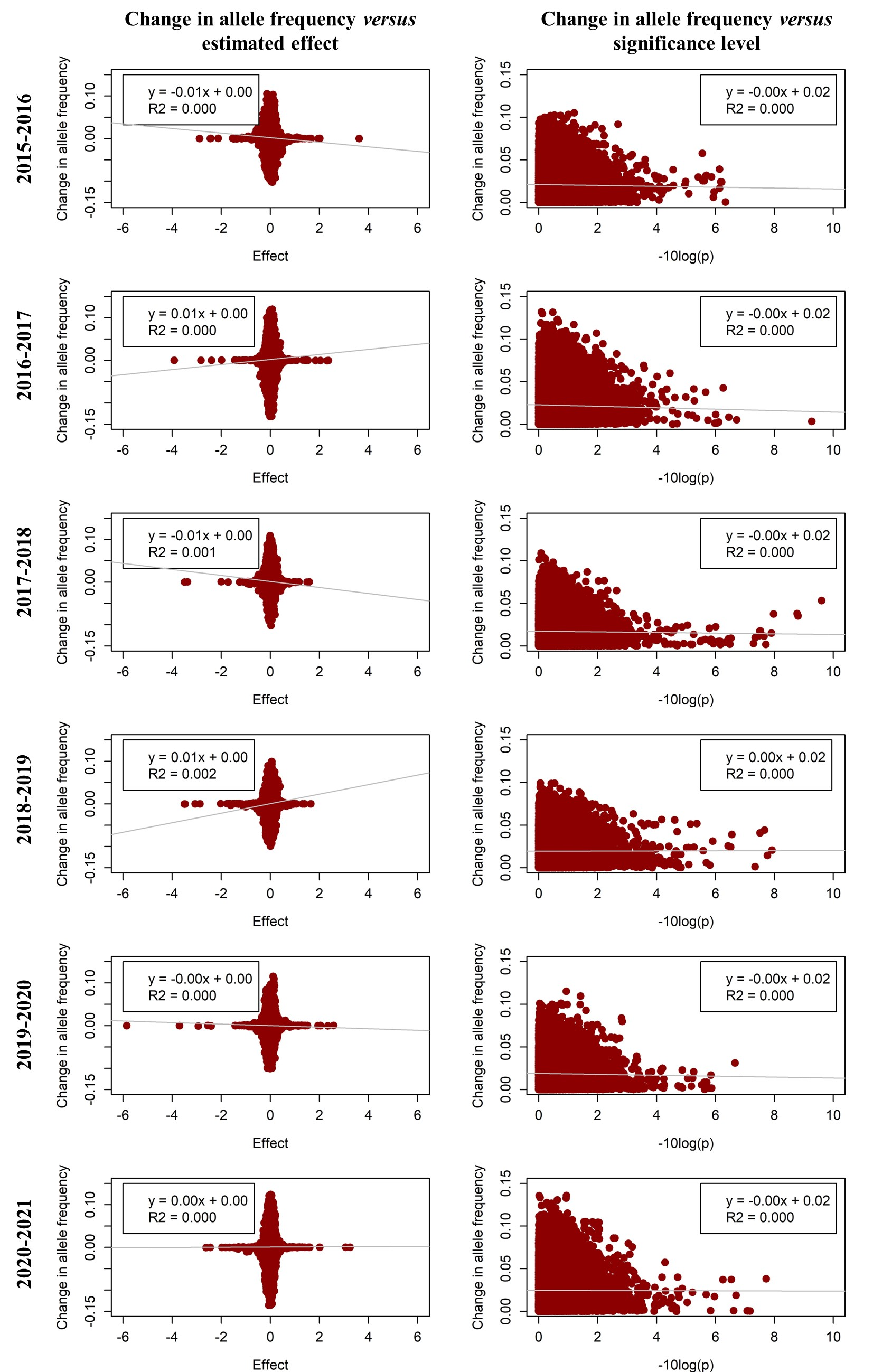


**Figure S3.2** Change in allele frequency versus estimated effect and significance level for fat depth in each year in line A. Estimated effects are from a GWAS per year, and the change in allele frequency is the change towards the next year, with the absolute value of allele frequency change for the significance level.


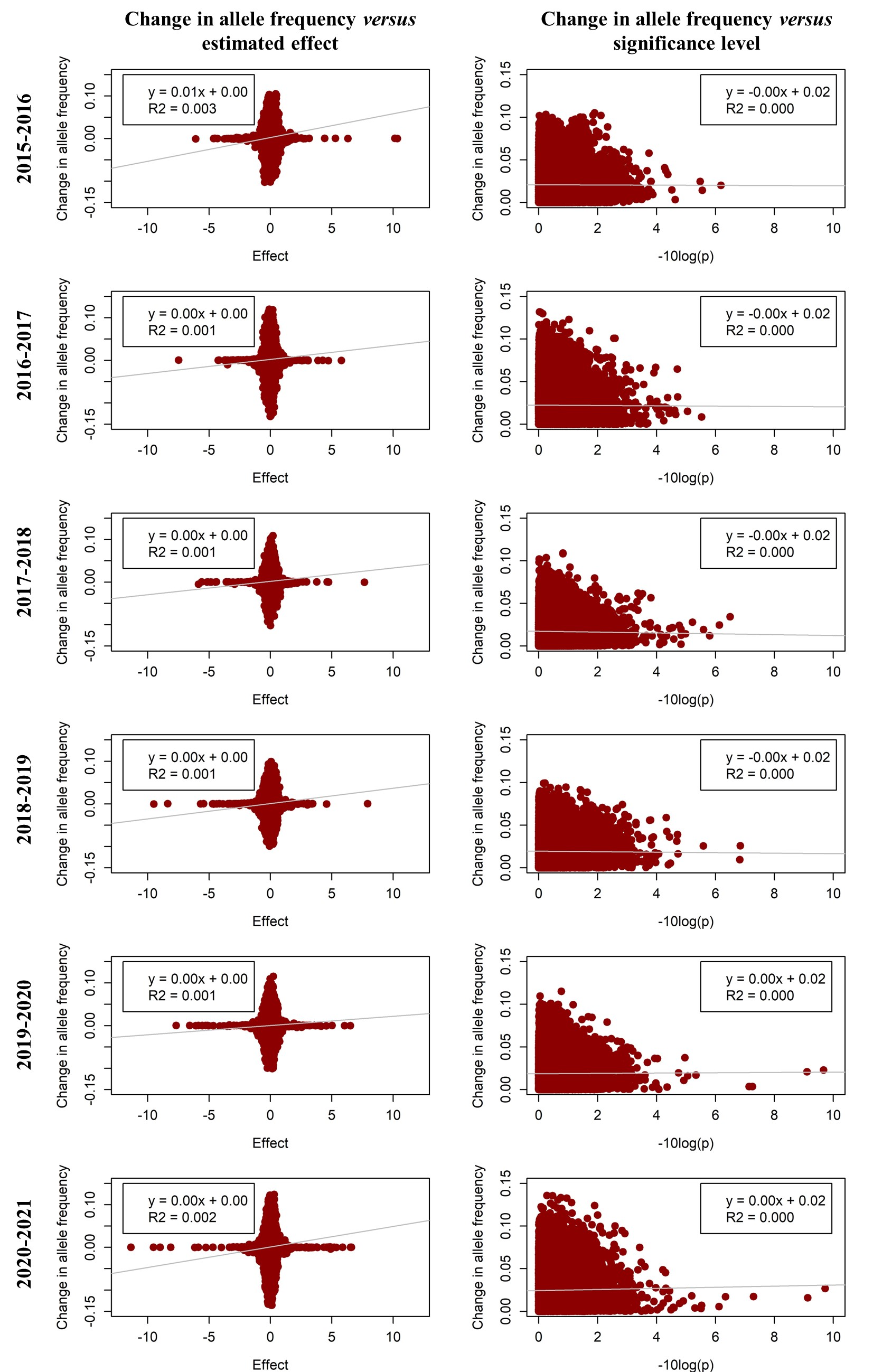


**Figure S3.3** Change in allele frequency versus estimated effect and significance level for muscle depth in each year in line A. Estimated effects are from a GWAS per year, and the change in allele frequency is the change towards the next year, with the absolute value of allele frequency change for the significance level.


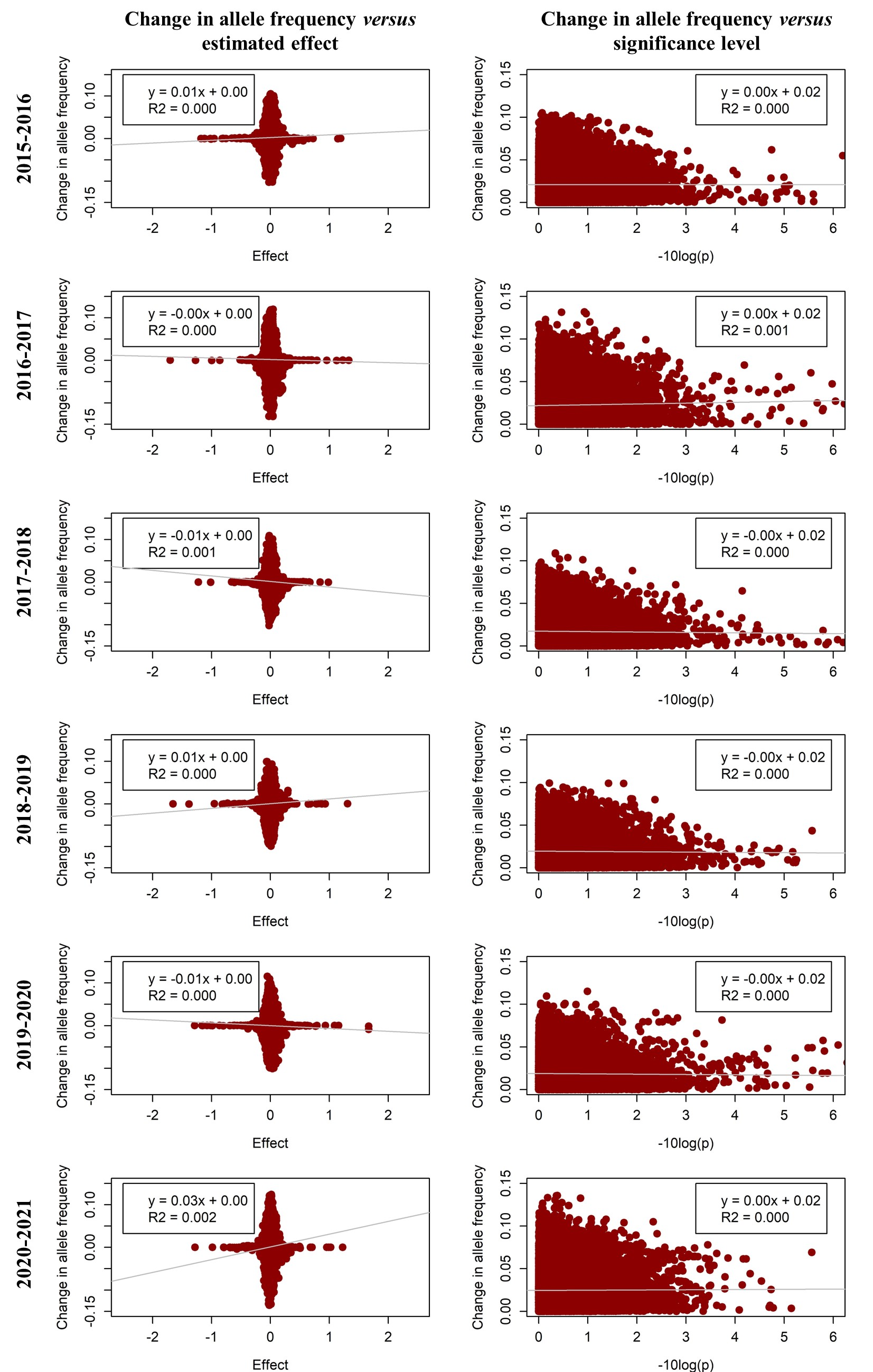


**Figure S3.4** Change in allele frequency versus estimated effect and significance level for number of teats in each year in line A. Estimated effects are from a GWAS per year, and the change in allele frequency is the change towards the next year, with the absolute value of allele frequency change for the significance level.


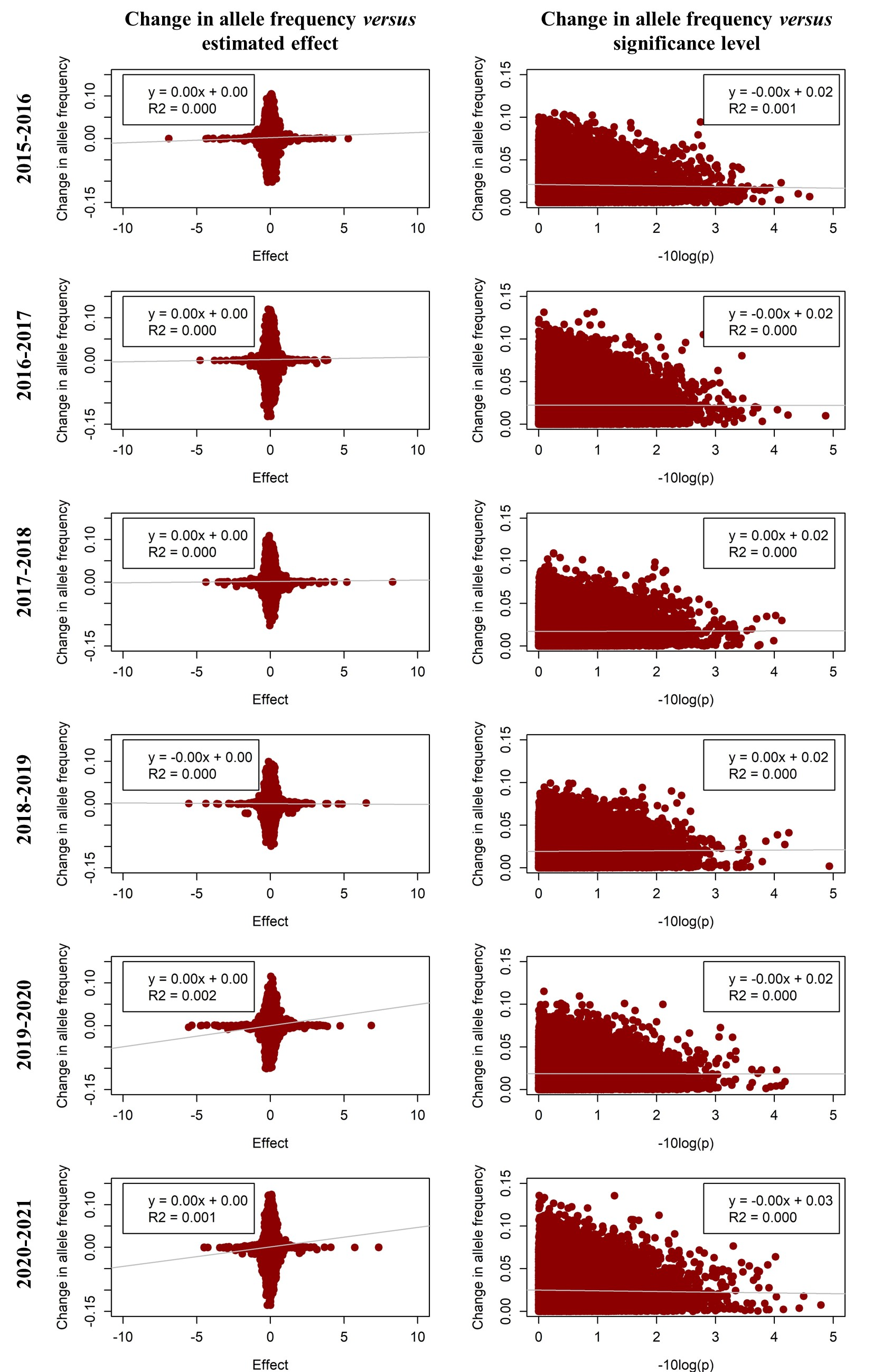


**Figure S3.5** Change in allele frequency versus estimated effect and significance level for total number born first parity in each year in line A. Estimated effects are from a GWAS per year, and the change in allele frequency is the change towards the next year, with the absolute value of allele frequency change for the significance level.


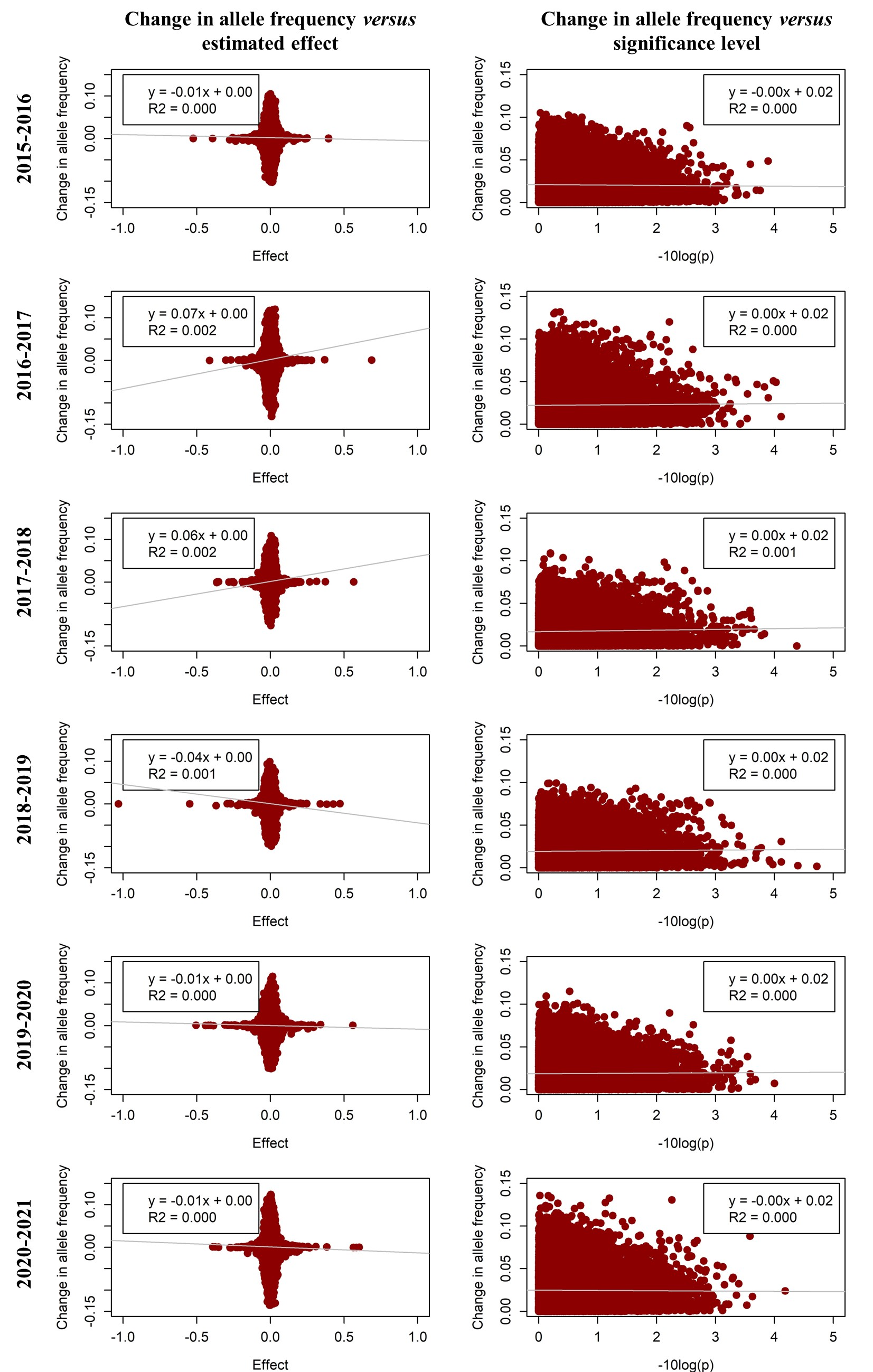


**Figure S3.6** Change in allele frequency versus estimated effect and significance level for average birth weight first litter in each year in line A. Estimated effects are from a GWAS per year, and the change in allele frequency is the change towards the next year, with the absolute value of allele frequency change for the significance level.


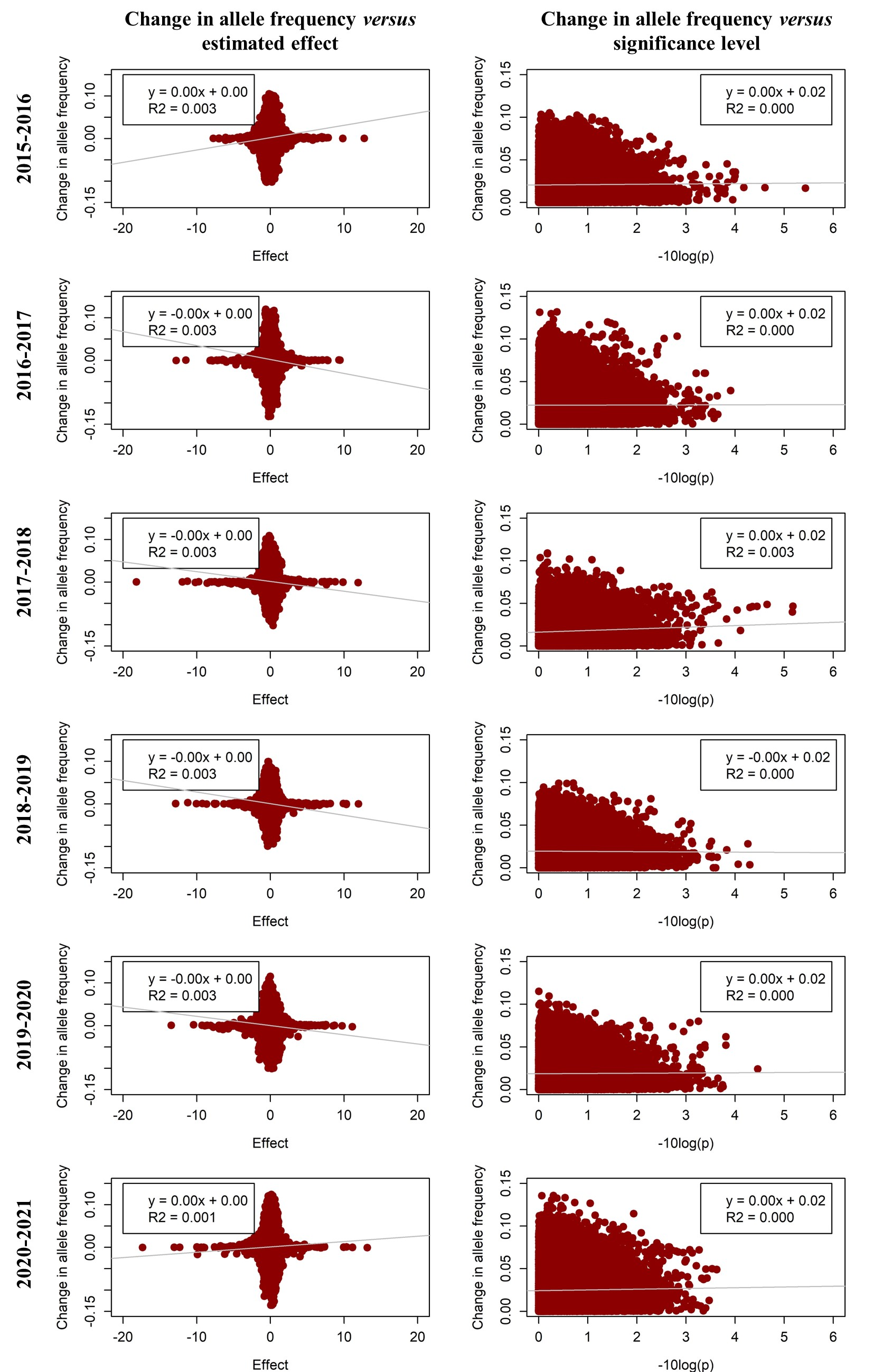


**Figure S3.7** Change in allele frequency versus estimated effect and significance level for CV of birth weight first litter in each year in line A. Estimated effects are from a GWAS per year, and the change in allele frequency is the change towards the next year, with the absolute value of allele frequency change for the significance level.


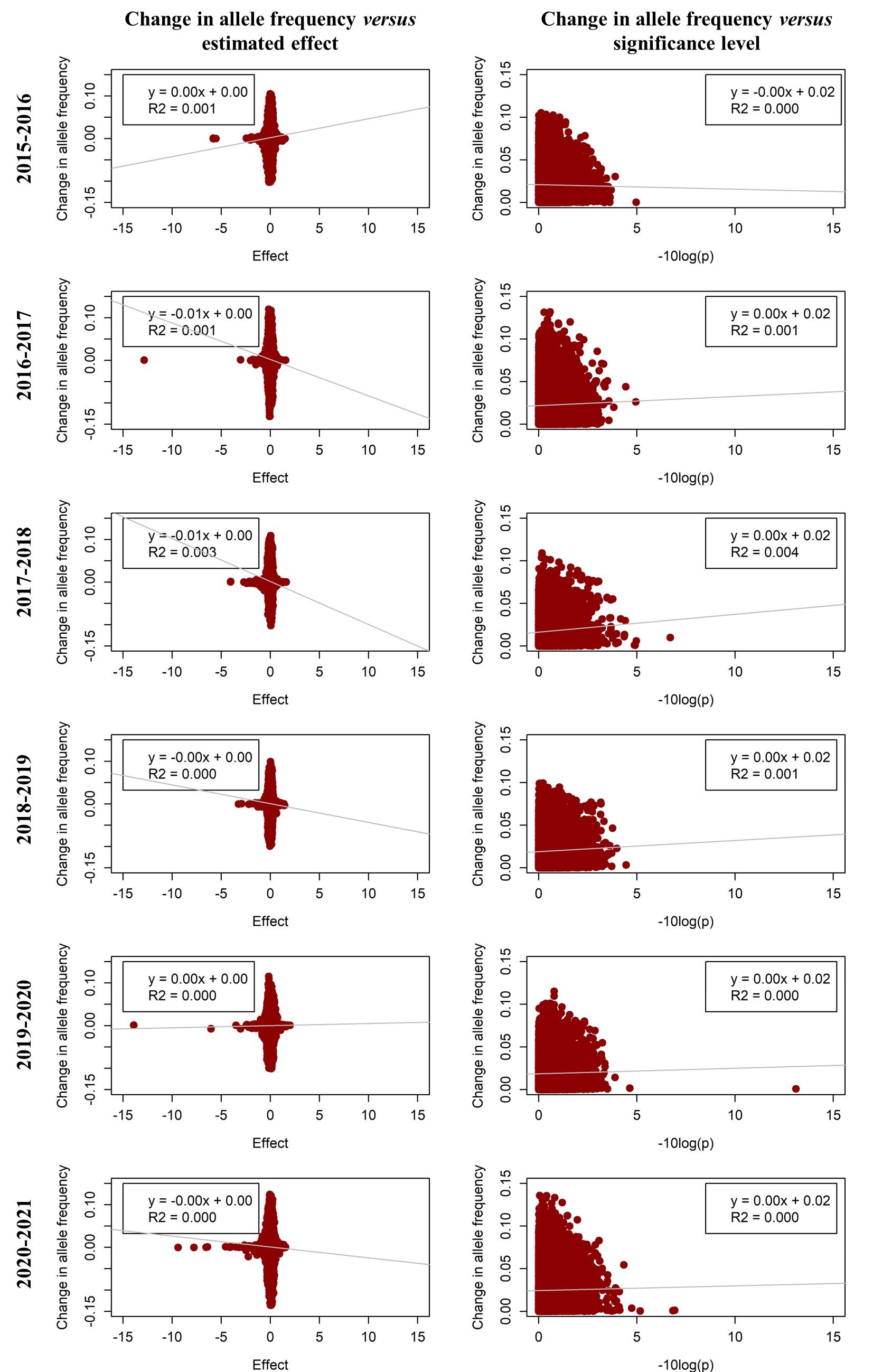


**Figure S3.8** Change in allele frequency versus estimated effect and significance level for number of small piglets in each year in line A. Estimated effects are from a GWAS per year, and the change in allele frequency is the change towards the next year, with the absolute value of allele frequency change for the significance level.


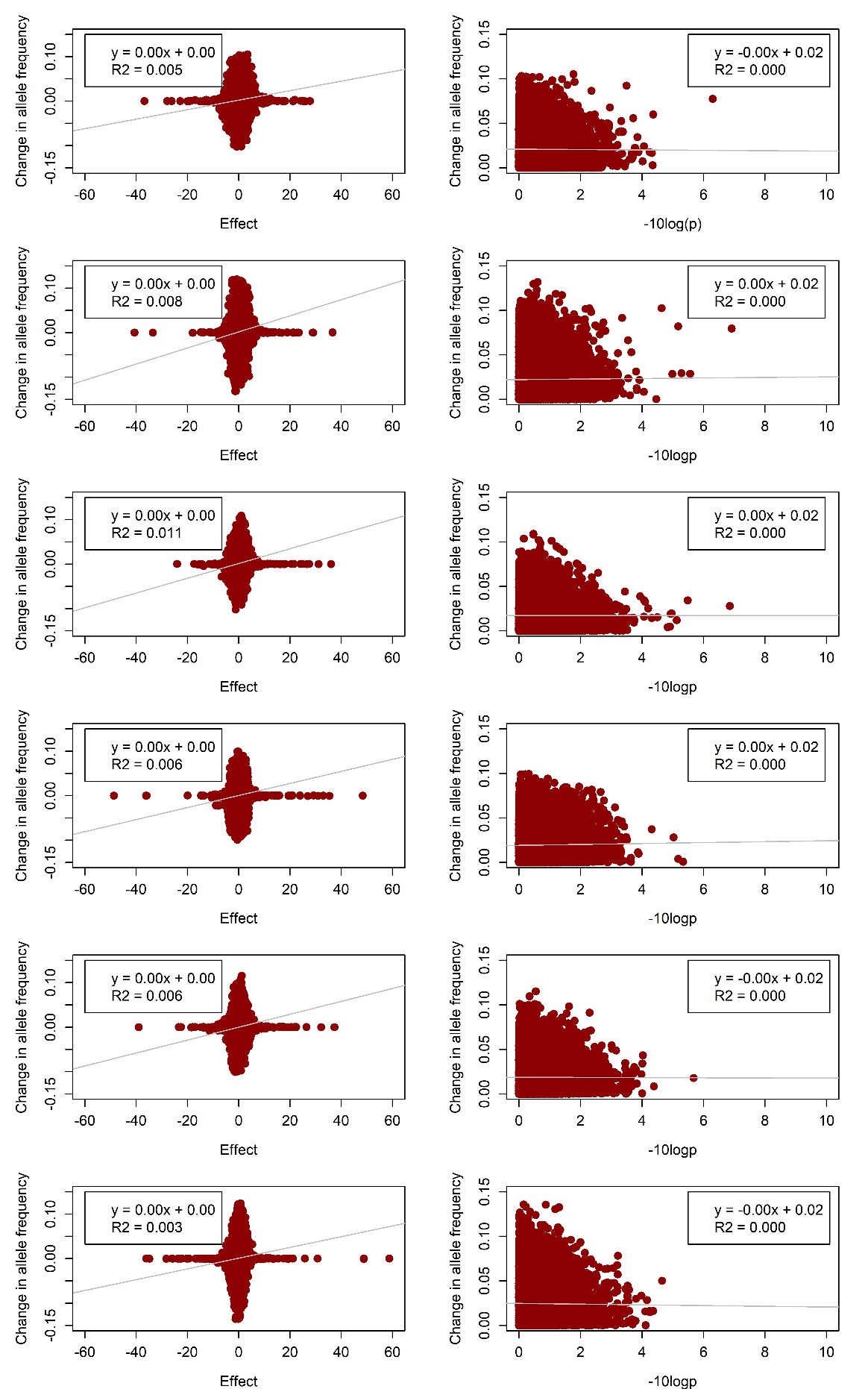


**Figure S3.9** Change in allele frequency versus estimated effect and significance level for the index in each year in line A. Estimated effects are from a GWAS per year, and the change in allele frequency is the change towards the next year, with the absolute value of allele frequency change for the significance level.


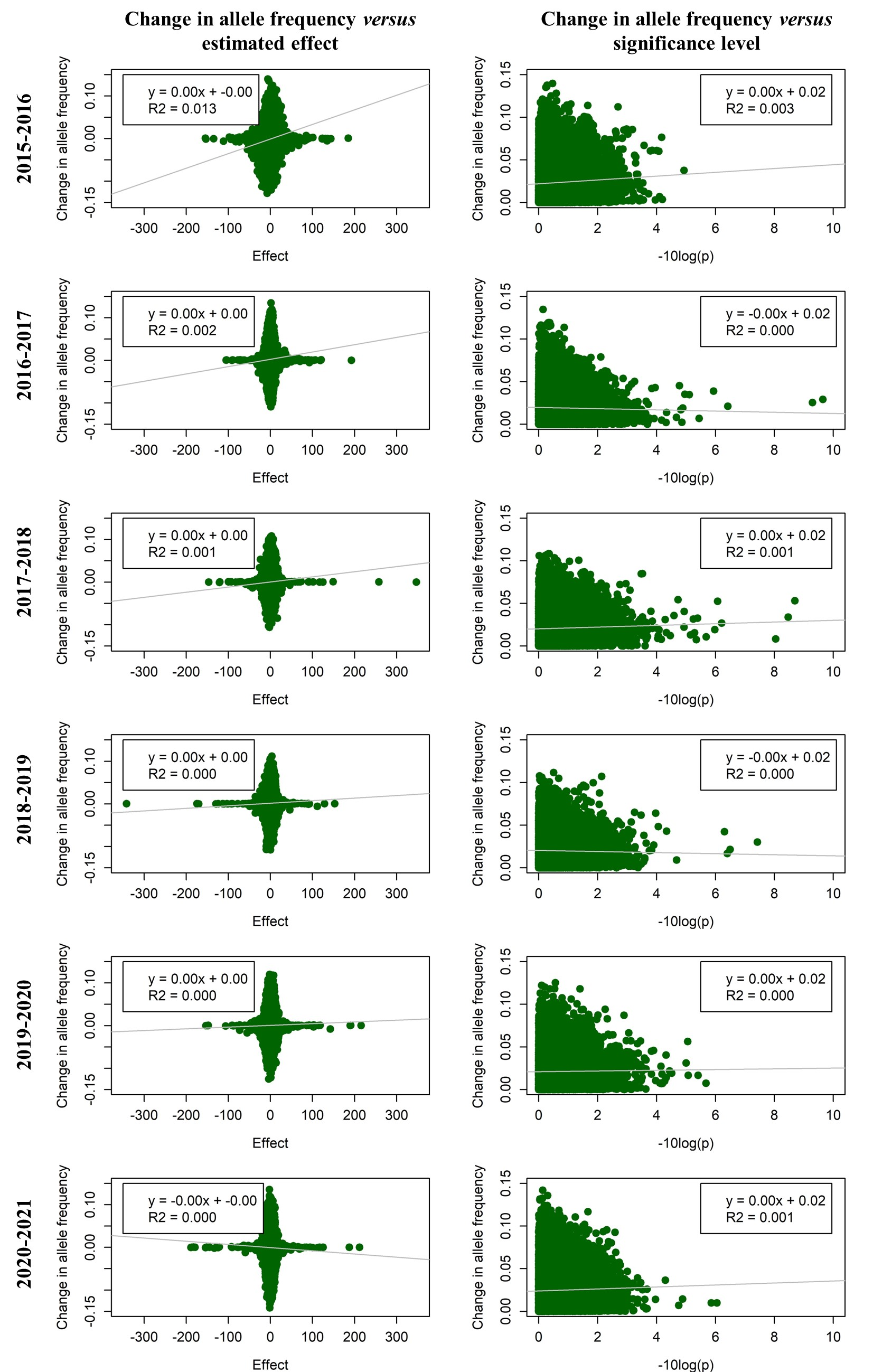


**Figure S3.10** Change in allele frequency versus estimated effect and significance level for daily gain in each year in line B. Estimated effects are from a GWAS per year, and the change in allele frequency is the change towards the next year, with the absolute value of allele frequency change for the significance level.


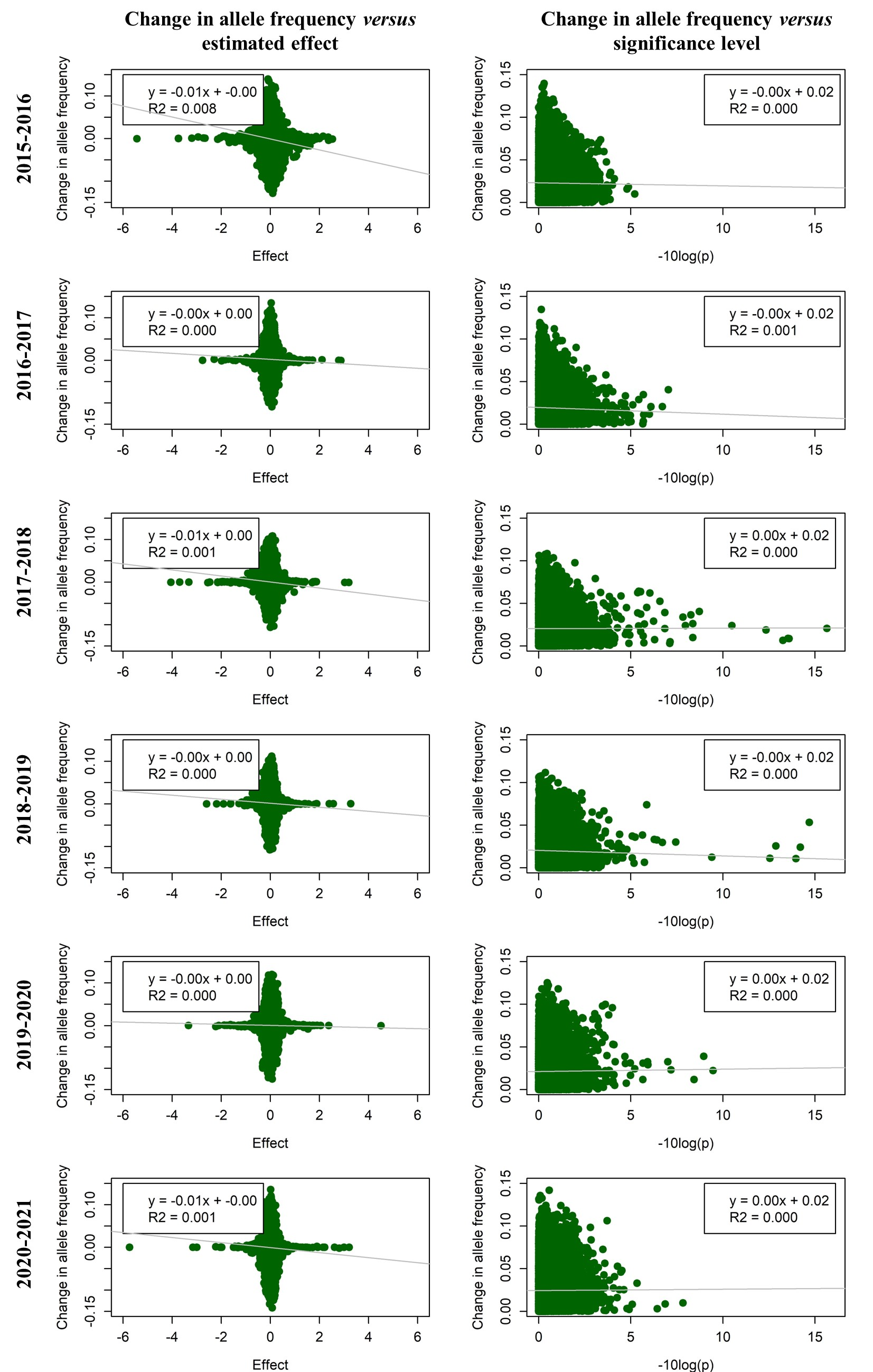


**Figure S3.11** Change in allele frequency versus estimated effect and significance level for fat depth in each year in line B. Estimated effects are from a GWAS per year, and the change in allele frequency is the change towards the next year, with the absolute value of allele frequency change for the significance level.


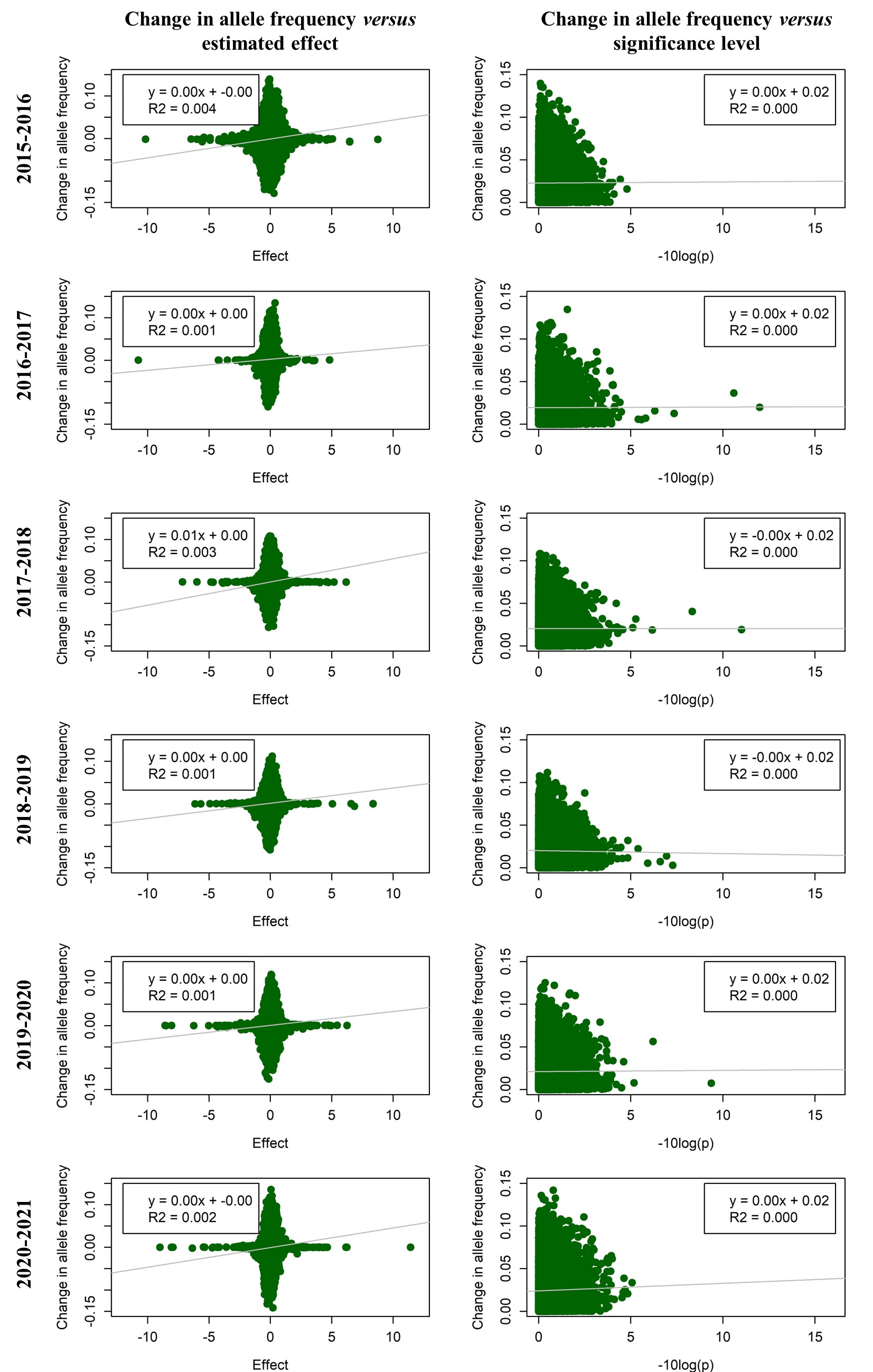


**Figure S3.12** Change in allele frequency versus estimated effect and significance level for muscle depth in each year in line B. Estimated effects are from a GWAS per year, and the change in allele frequency is the change towards the next year, with the absolute value of allele frequency change for the significance level.


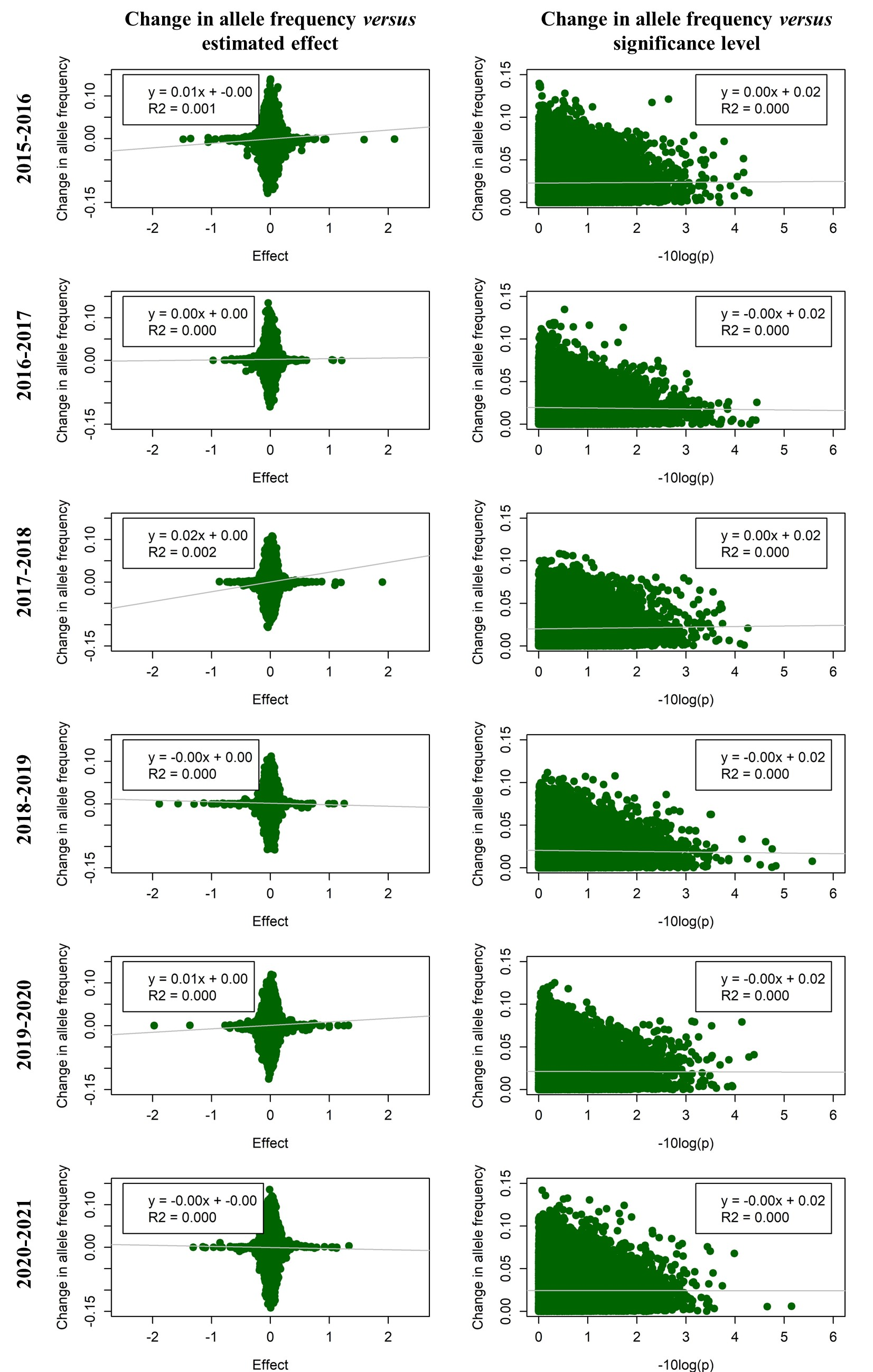


**Figure S3.13** Change in allele frequency versus estimated effect and significance level for number of teats in each year in line B. Estimated effects are from a GWAS per year, and the change in allele frequency is the change towards the next year, with the absolute value of allele frequency change for the significance level.


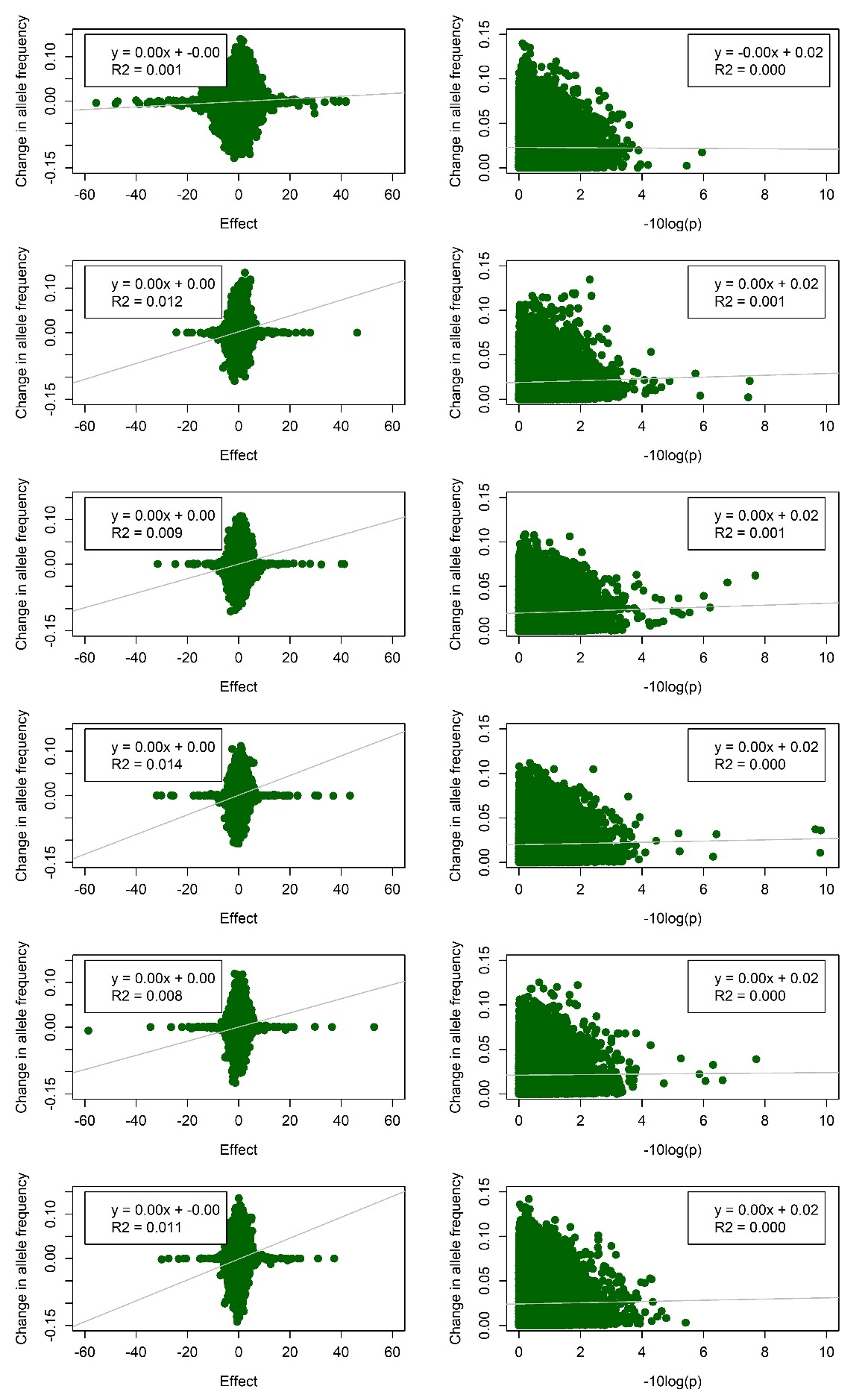


**Figure S3.14** Change in allele frequency versus estimated effect and significance level for the index in each year in line B. Estimated effects are from a GWAS per year, and the change in allele frequency is the change towards the next year, with the absolute value of allele frequency change for the significance level.
