# Supplementary material for: Changes in allele frequencies and genetic architecture due to selection in two pig populations
Source: Genet Sel Evol. 2024 Dec 17;56:76. doi: 10.1186/s12711-024-00941-3 (PMC11650847; doi:10.1186/s12711-024-00941-3)
Supplement: Supplementary file 4 — Additional file 4. Correlation allele frequency change and GWAS results for loci with MAF > 0.1. Fourteen figures describing the correlation between allele frequency change and GWAS results (estimated effect and significance level) for the loci with a minor allele frequency above 0.1. [file 12711_2024_941_MOESM4_ESM.docx]

**Additional file 4: Correlation allele frequency change and GWAS results for loci with MAF > 0.1**


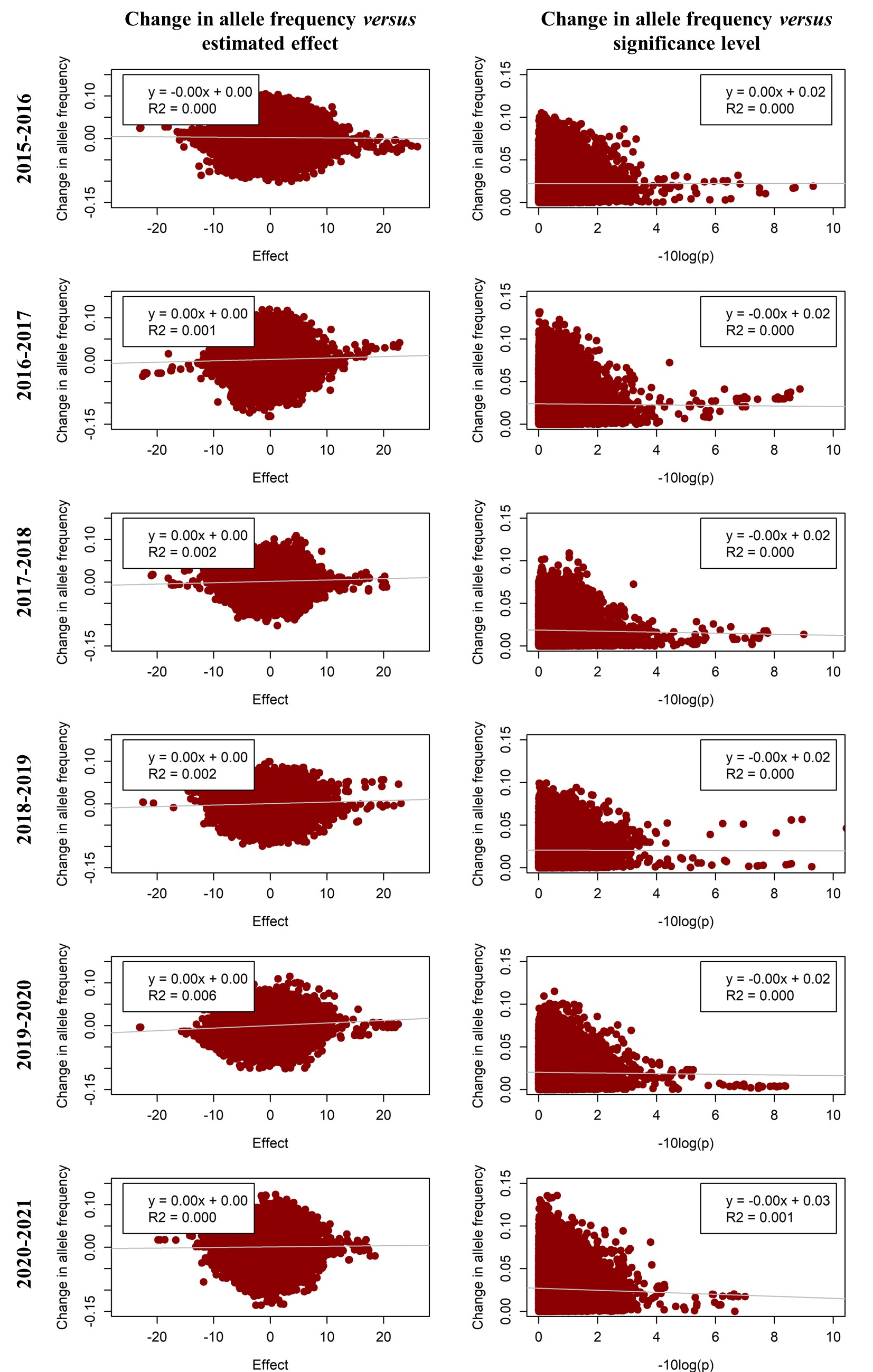


**Figure S4.1** Change in allele frequency versus estimated effect and significance level for daily gain in each year in line A using only loci with a MAF above 0.1. Estimated effects are from a GWAS per year, and the change in allele frequency is the change towards the next year, with the absolute value of allele frequency change for the significance level.


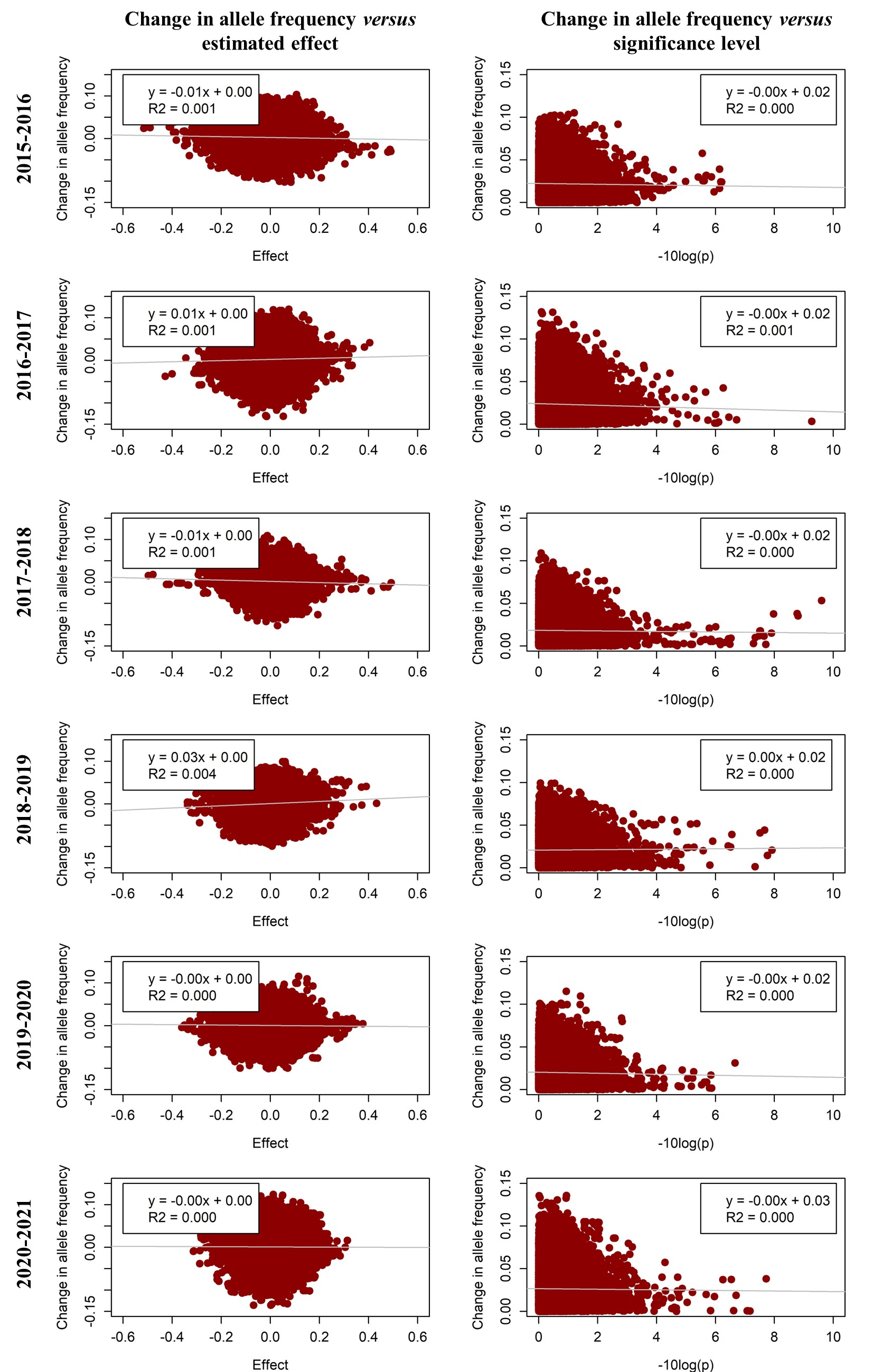


**Figure S4.2** Change in allele frequency versus estimated effect and significance level for fat depth in each year in line A using only loci with a MAF above 0.1. Estimated effects are from a GWAS per year, and the change in allele frequency is the change towards the next year, with the absolute value of allele frequency change for the significance level.


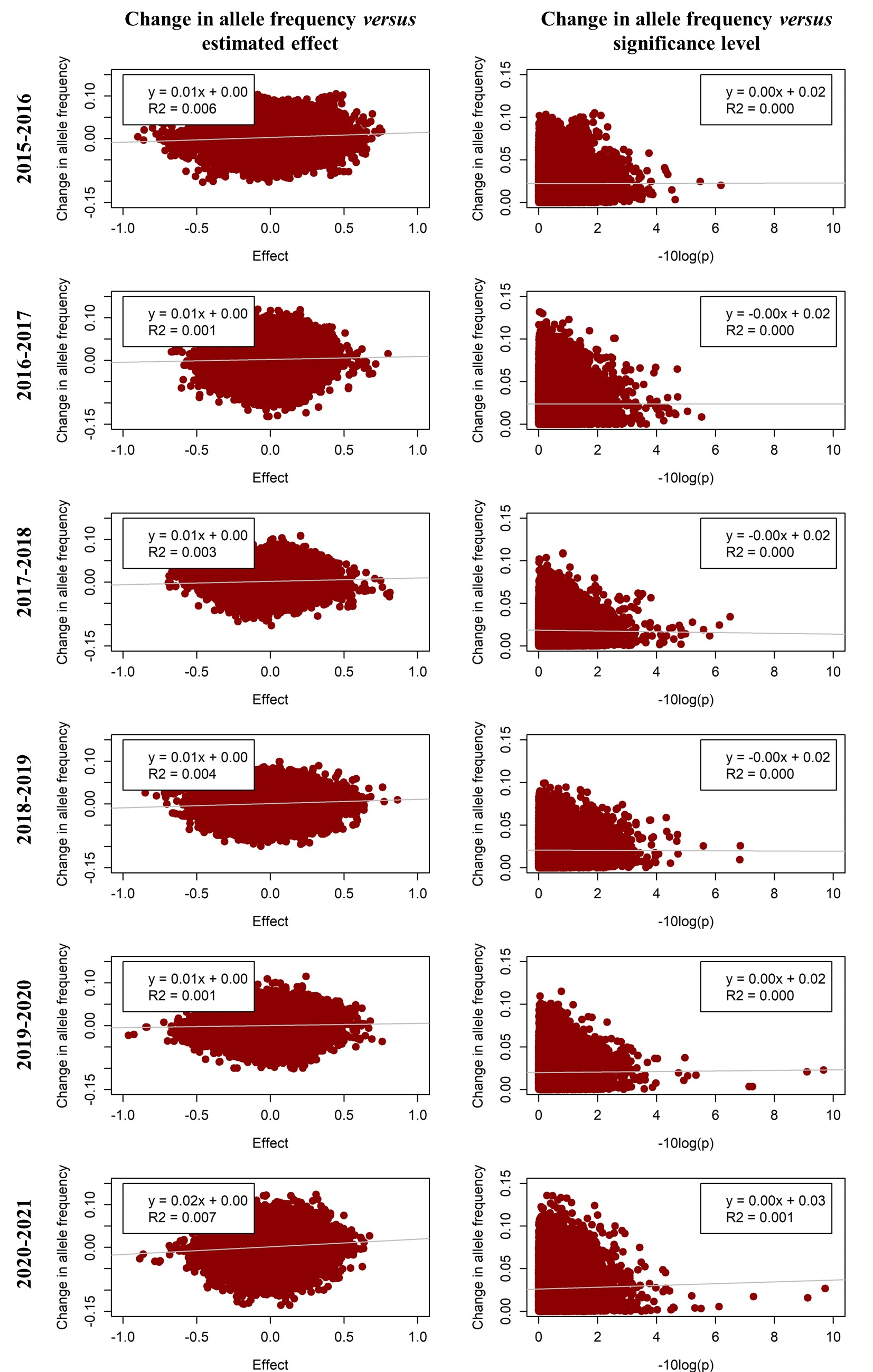


**Figure S4.3** Change in allele frequency versus estimated effect and significance level for muscle depth in each year in line A using only loci with a MAF above 0.1. Estimated effects are from a GWAS per year, and the change in allele frequency is the change towards the next year, with the absolute value of allele frequency change for the significance level.


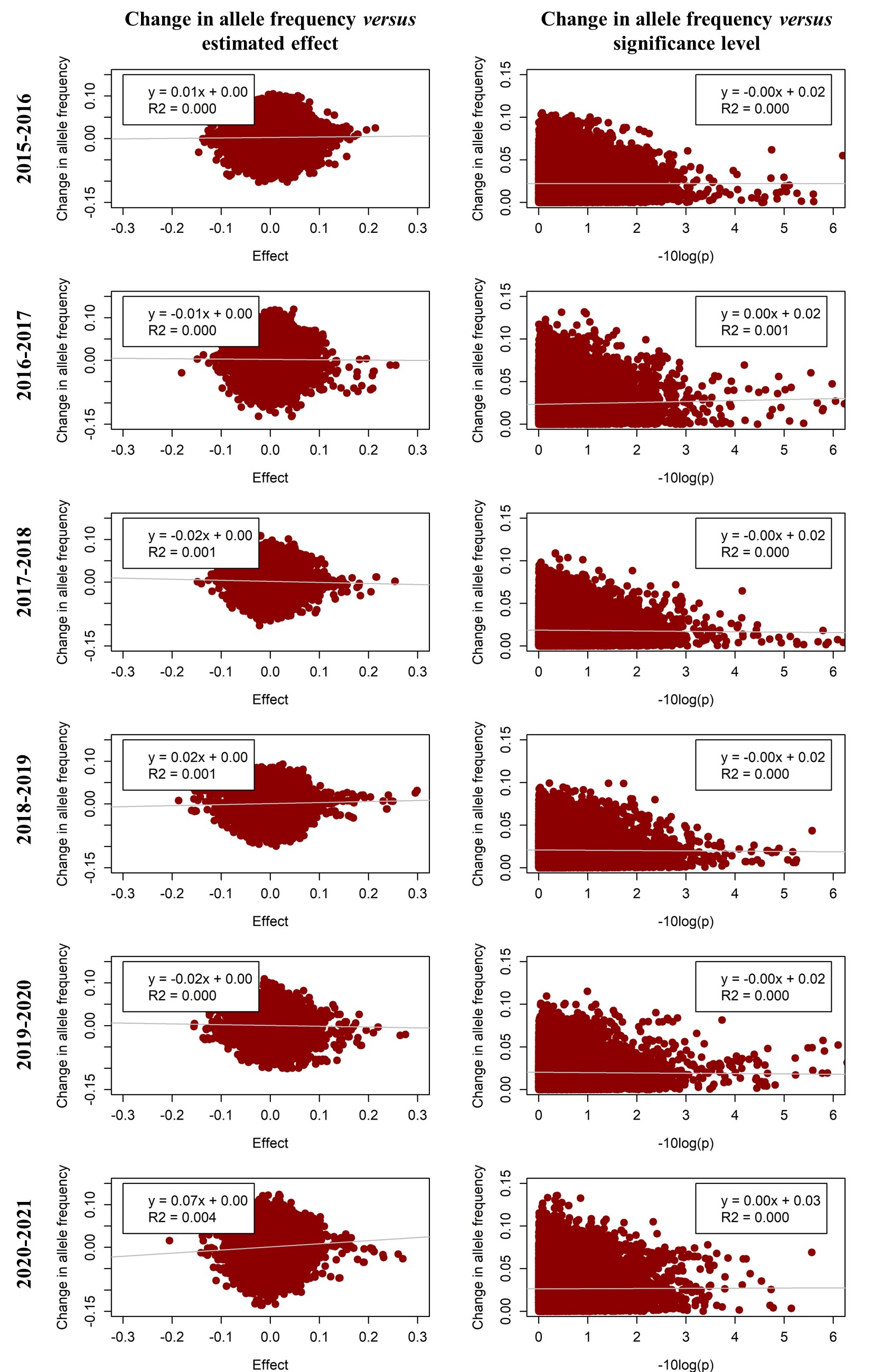


**Figure S4.4** Change in allele frequency versus estimated effect and significance level for number of teats in each year in line A using only loci with a MAF above 0.1. Estimated effects are from a GWAS per year, and the change in allele frequency is the change towards the next year, with the absolute value of allele frequency change for the significance level.


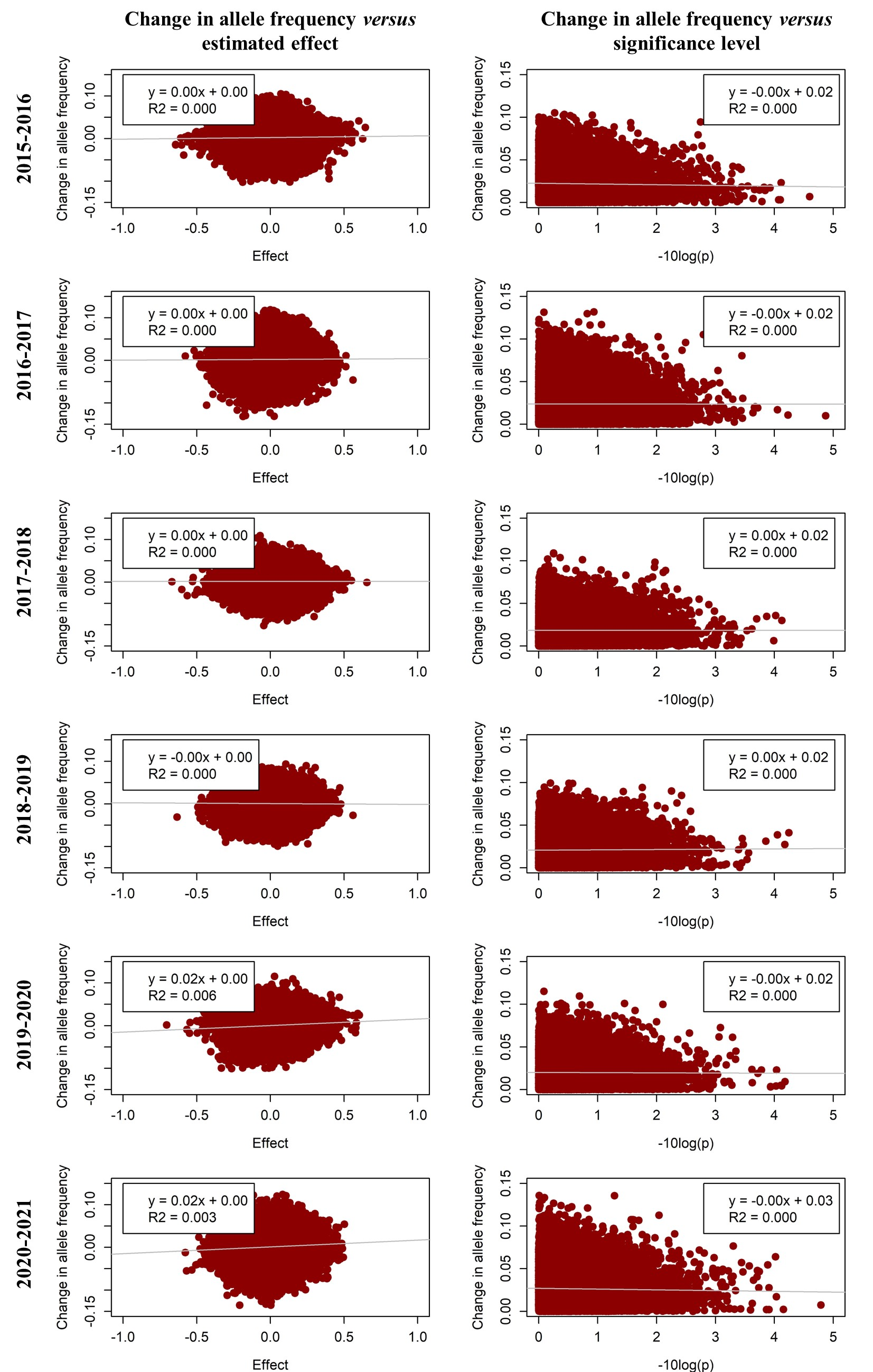


**Figure S4.5** Change in allele frequency versus estimated effect and significance level for total number born first parity in each year in line A using only loci with a MAF above 0.1. Estimated effects are from a GWAS per year, and the change in allele frequency is the change towards the next year, with the absolute value of allele frequency change for the significance level.


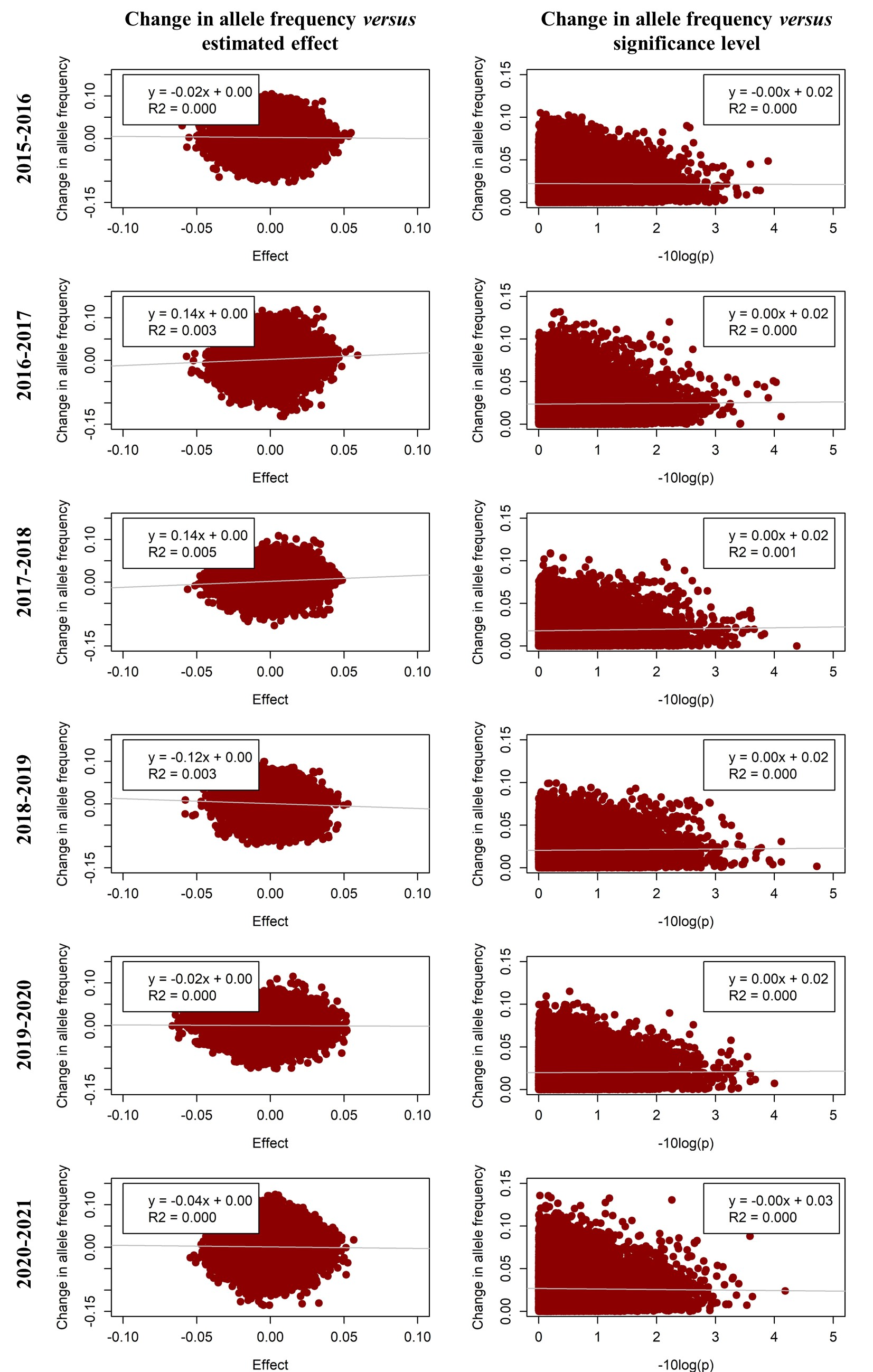


**Figure S4.6** Change in allele frequency versus estimated effect and significance level for average birth weight first litter in each year in line A using only loci with a MAF above 0.1. Estimated effects are from a GWAS per year, and the change in allele frequency is the change towards the next year, with the absolute value of allele frequency change for the significance level.


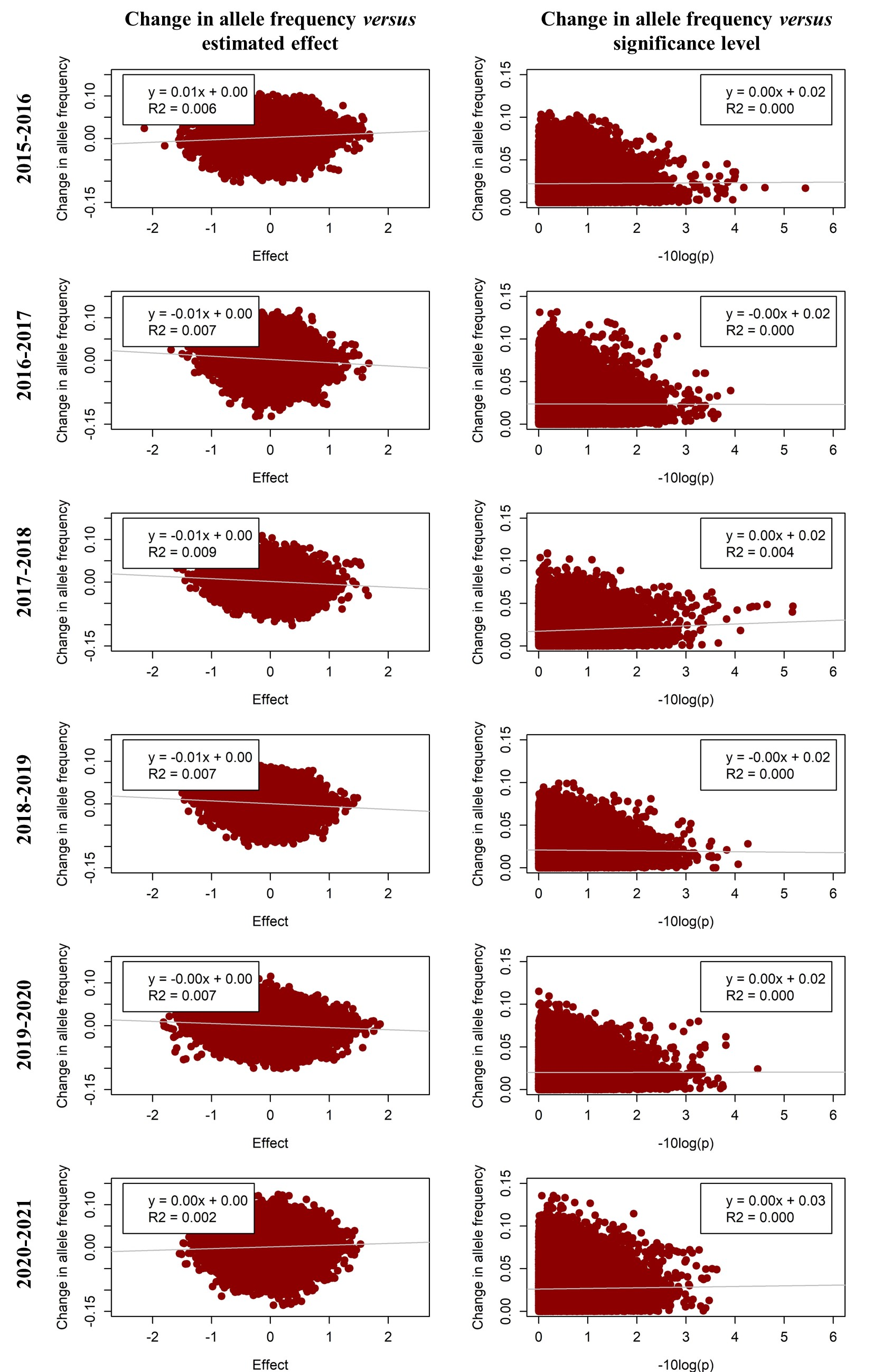


**Figure S4.7** Change in allele frequency versus estimated effect and significance level for CV of birth weight first litter in each year in line A using only loci with a MAF above 0.1. Estimated effects are from a GWAS per year, and the change in allele frequency is the change towards the next year, with the absolute value of allele frequency change for the significance level.


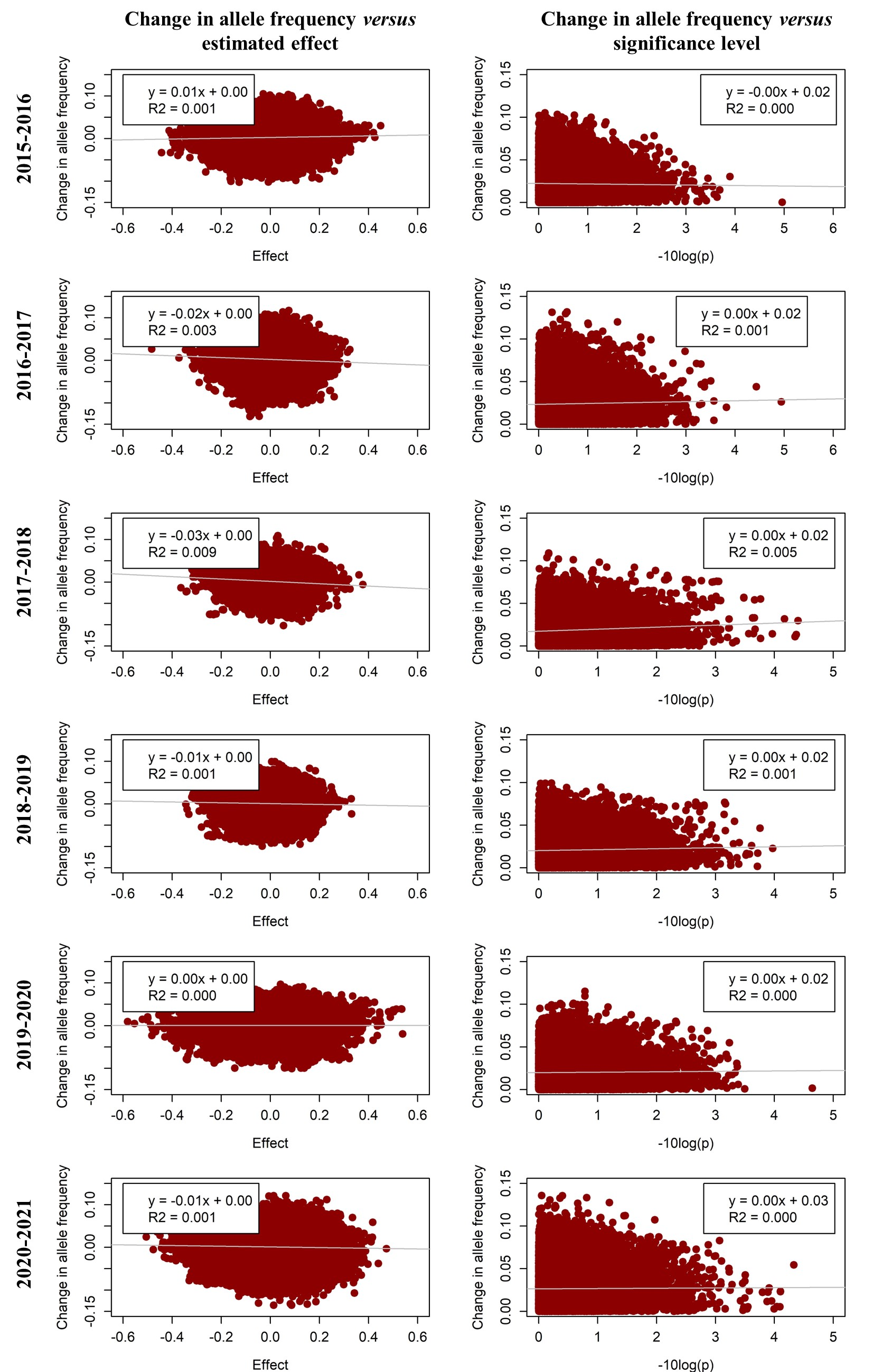


**Figure S4.8** Change in allele frequency versus estimated effect and significance level for number of small piglets in each year in line A using only loci with a MAF above 0.1. Estimated effects are from a GWAS per year, and the change in allele frequency is the change towards the next year, with the absolute value of allele frequency change for the significance level.


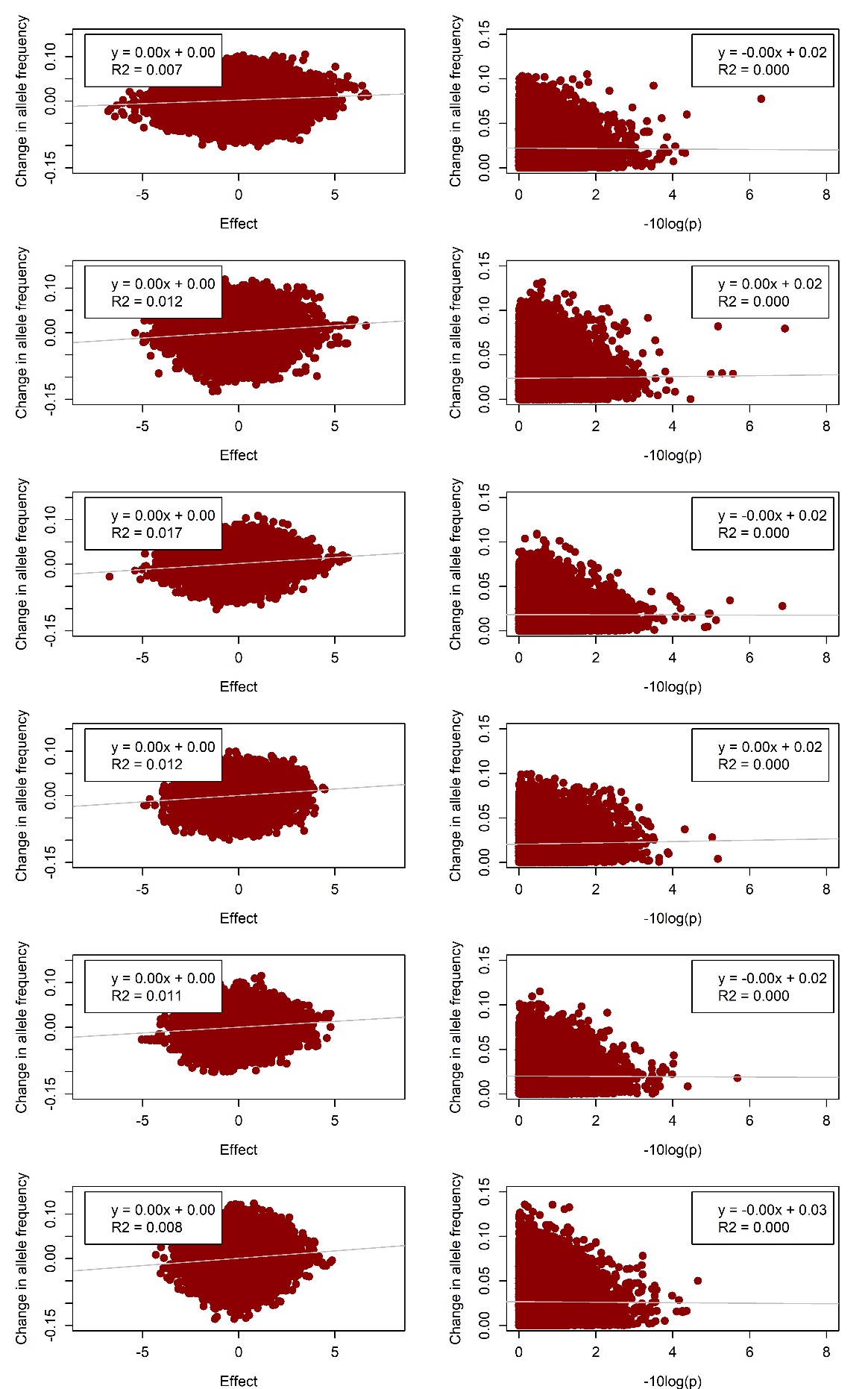


**Figure S4.9** Change in allele frequency versus estimated effect and significance level for the index in each year in line A using only loci with a MAF above 0.1. Estimated effects are from a GWAS per year, and the change in allele frequency is the change towards the next year, with the absolute value of allele frequency change for the significance level.


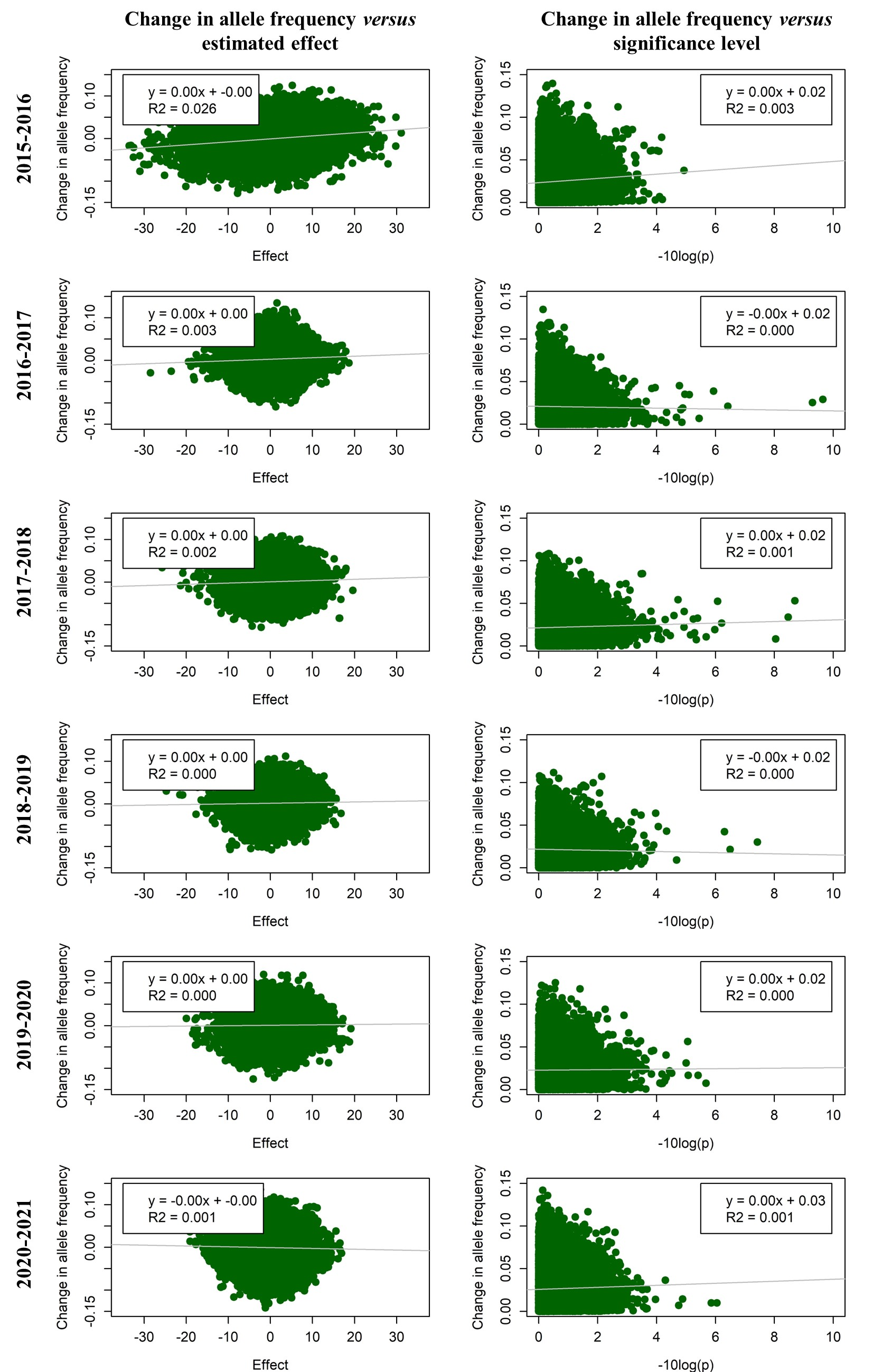


**Figure S4.10** Change in allele frequency versus estimated effect and significance level for daily gain in each year in line B using only loci with a MAF above 0.1. Estimated effects are from a GWAS per year, and the change in allele frequency is the change towards the next year, with the absolute value of allele frequency change for the significance level.


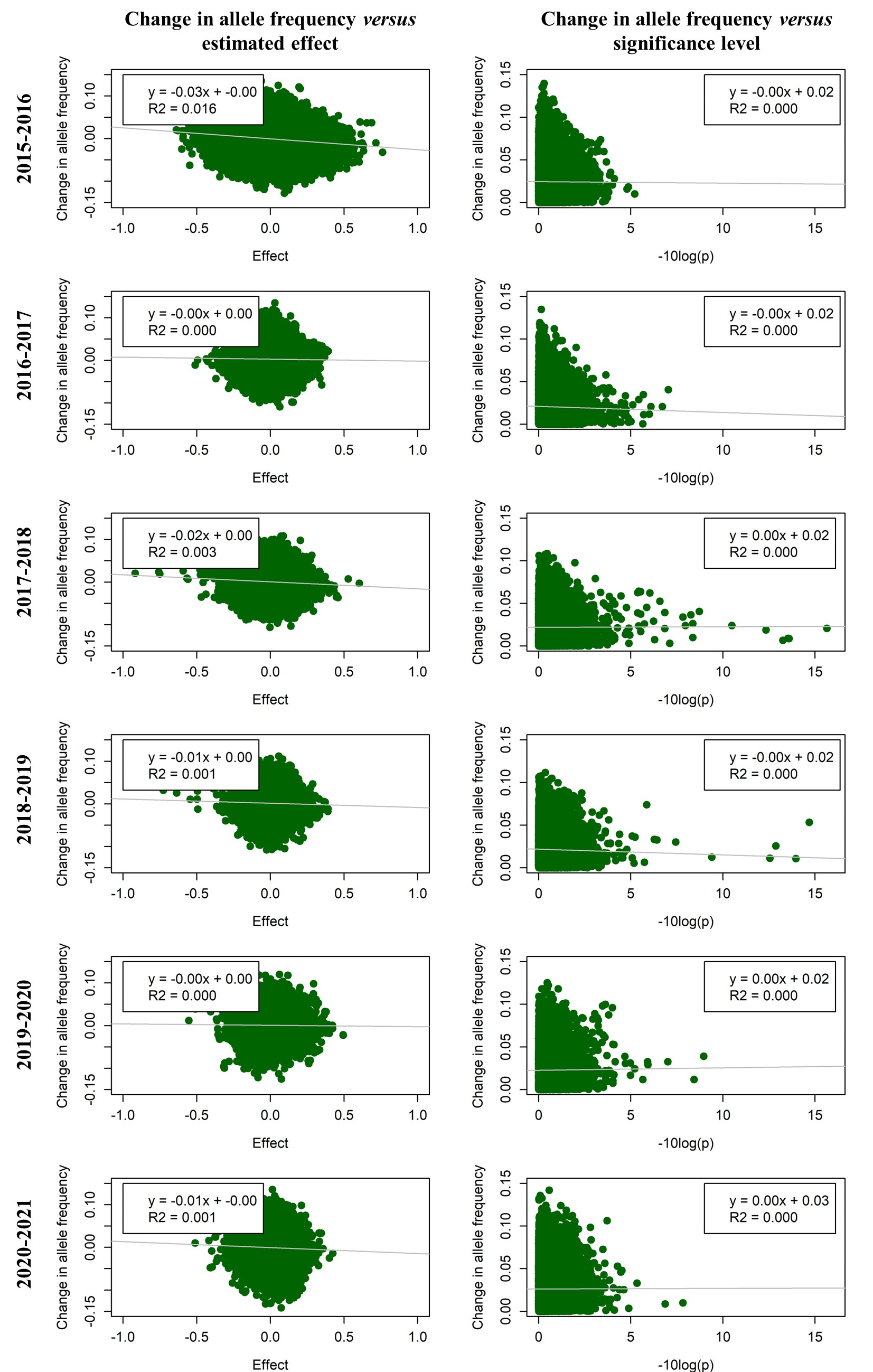


**Figure S4.11** Change in allele frequency versus estimated effect and significance level for fat depth in each year in line B using only loci with a MAF above 0.1. Estimated effects are from a GWAS per year, and the change in allele frequency is the change towards the next year, with the absolute value of allele frequency change for the significance level.


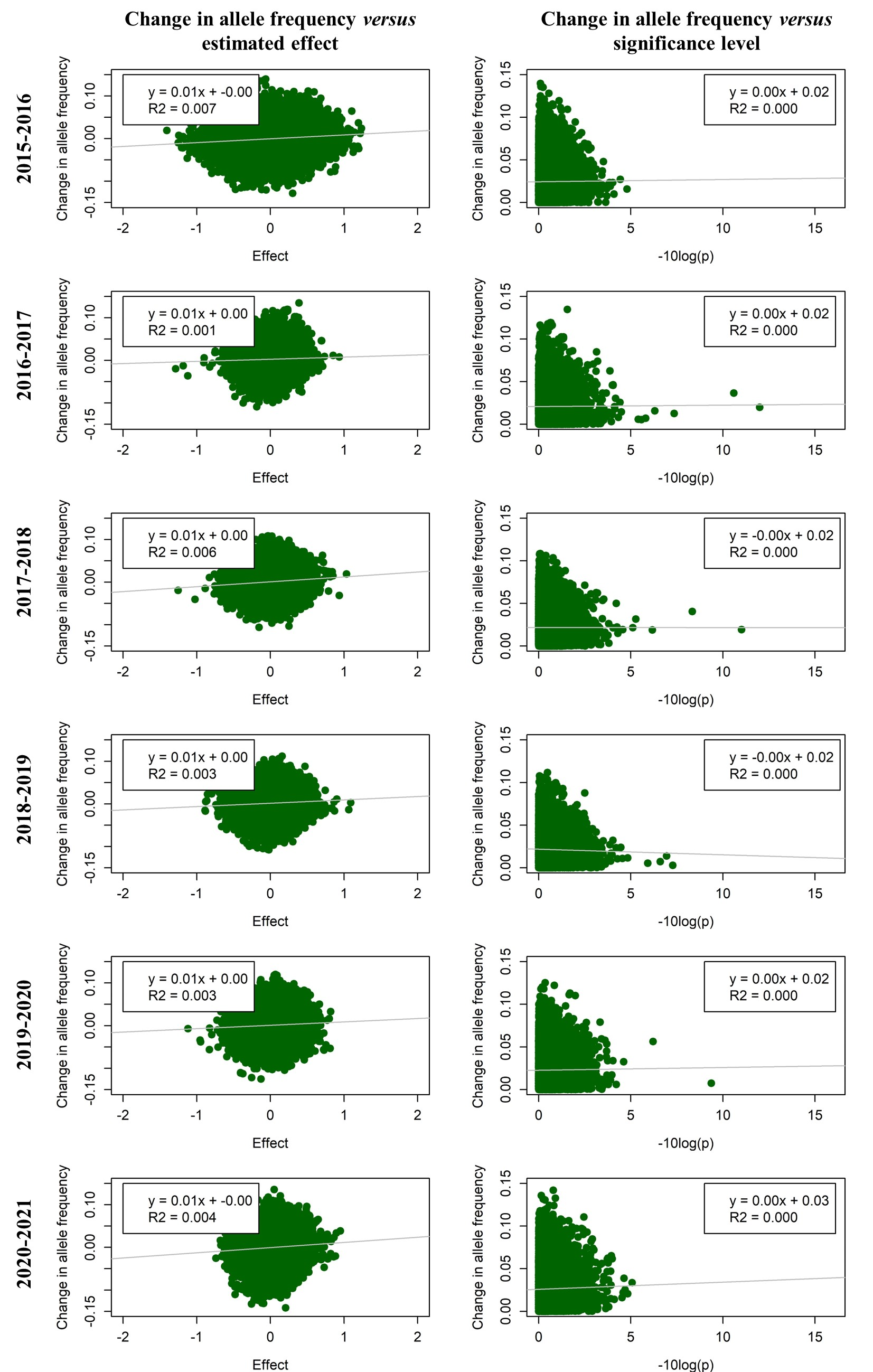


**Figure S4.12** Change in allele frequency versus estimated effect and significance level for muscle depth in each year in line B using only loci with a MAF above 0.1. Estimated effects are from a GWAS per year, and the change in allele frequency is the change towards the next year, with the absolute value of allele frequency change for the significance level.


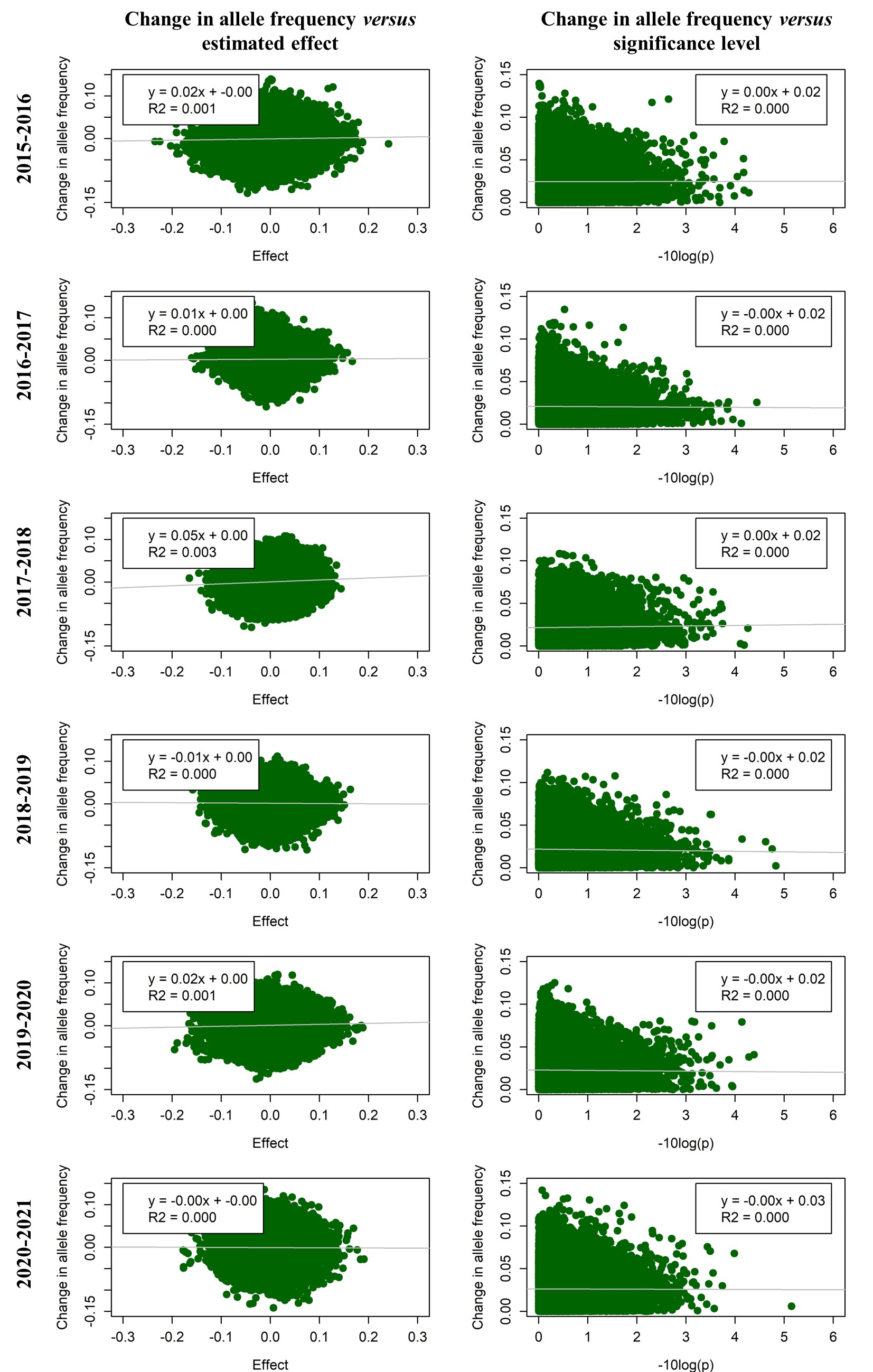


**Figure S4.13** Change in allele frequency versus estimated effect and significance level for number of teats in each year in line B using only loci with a MAF above 0.1. Estimated effects are from a GWAS per year, and the change in allele frequency is the change towards the next year, with the absolute value of allele frequency change for the significance level.


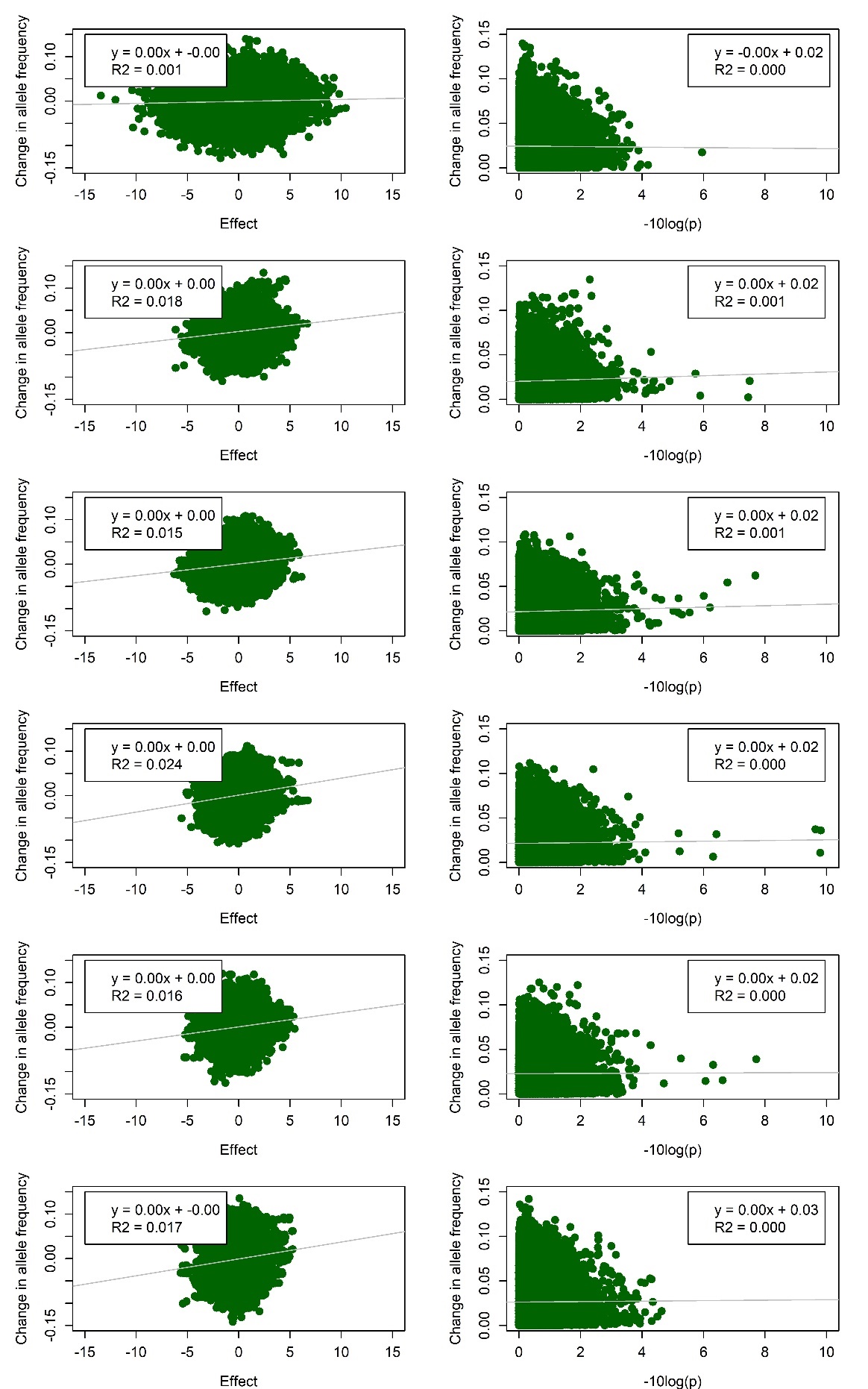


**Figure S4.14** Change in allele frequency versus estimated effect and significance level for the index in each year in line B using only loci with a MAF above 0.1. Estimated effects are from a GWAS per year, and the change in allele frequency is the change towards the next year, with the absolute value of allele frequency change for the significance level.
