# Supplementary material for: Changes in allele frequencies and genetic architecture due to selection in two pig populations
Source: Genet Sel Evol. 2024 Dec 17;56:76. doi: 10.1186/s12711-024-00941-3 (PMC11650847; doi:10.1186/s12711-024-00941-3)
Supplement: Supplementary file 5 — Additional file 5. GWAS results across years. Thirteen figures with the Manhattan plots for the different GWAS analyses across the different years. [file 12711_2024_941_MOESM5_ESM.docx]

**Additional file 5: GWAS results across years**


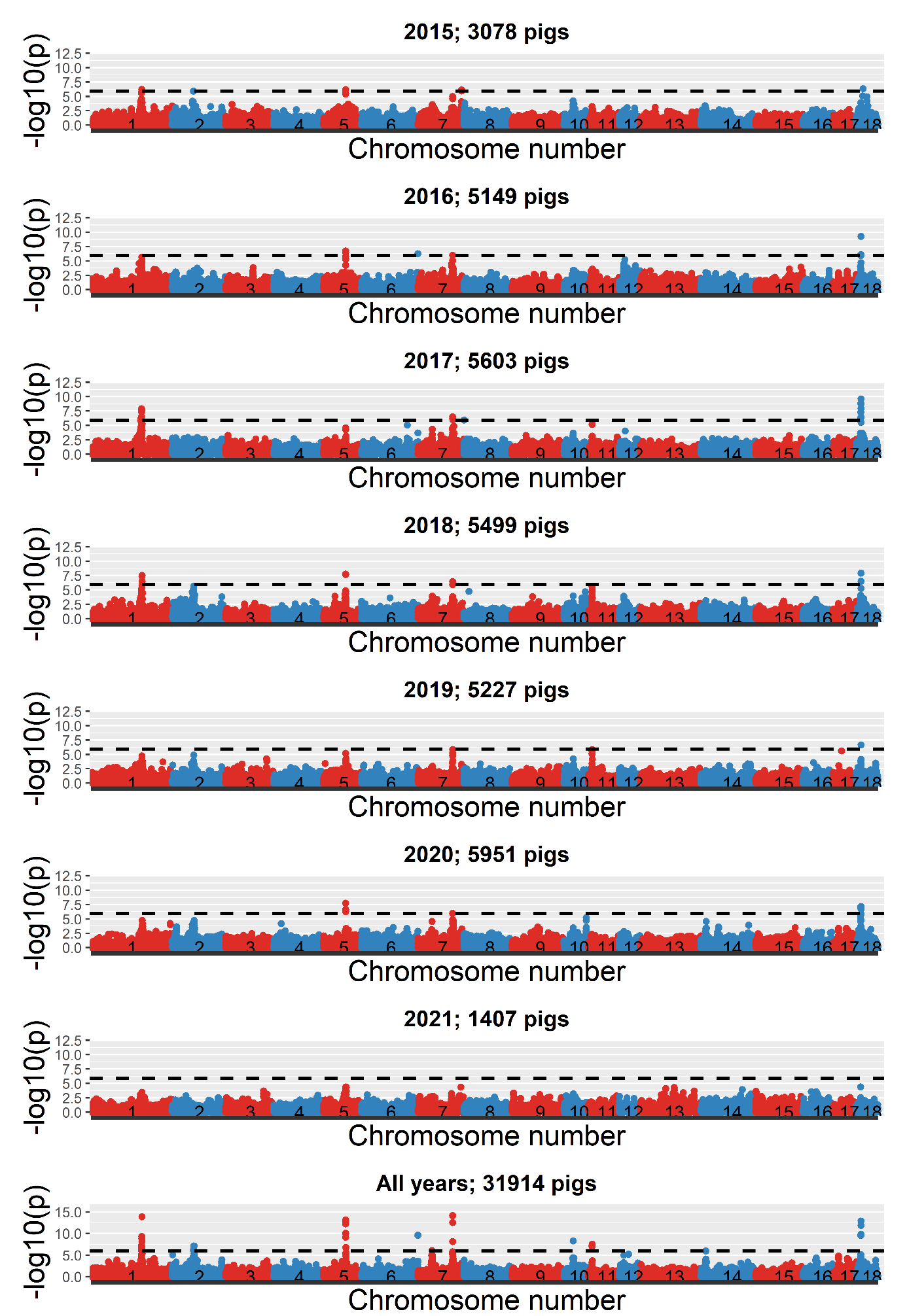


**Figure S5.1** Manhattan plots for fat depth in line A for the different years.


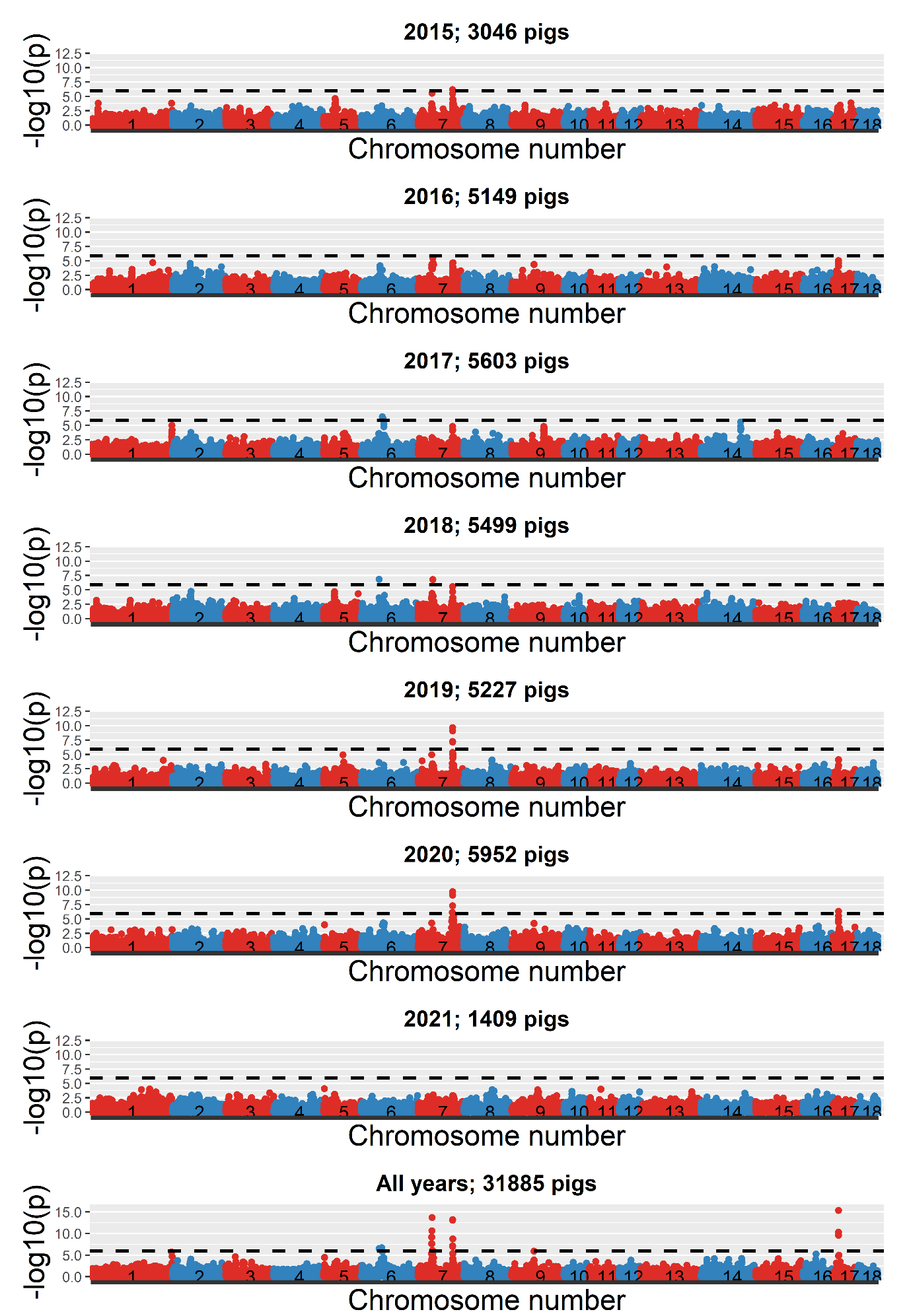


**Figure S5.2** Manhattan plots for muscle depth in line A for the different years.


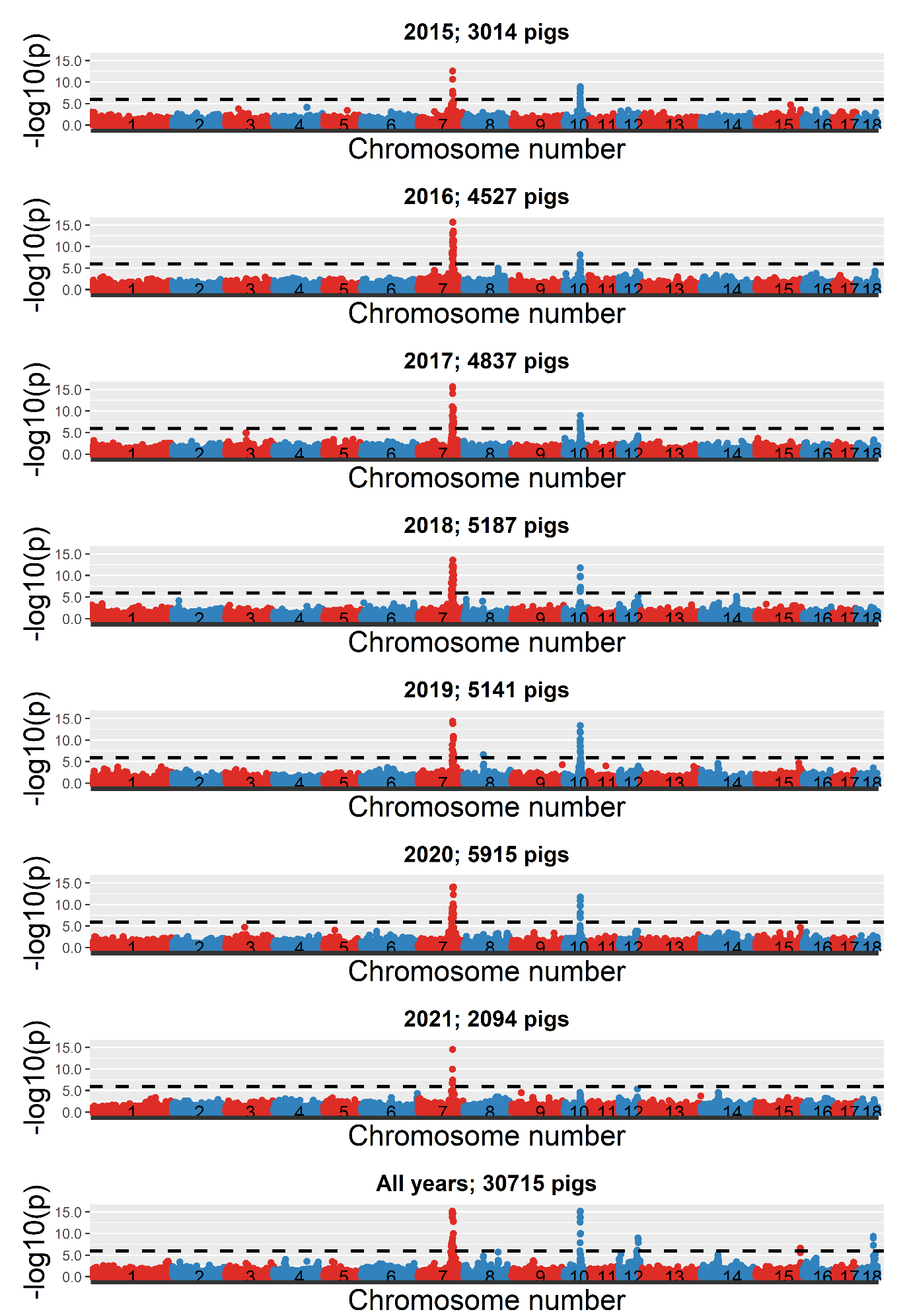


**Figure S5.3** Manhattan plots for number of teats in line A for the different years.


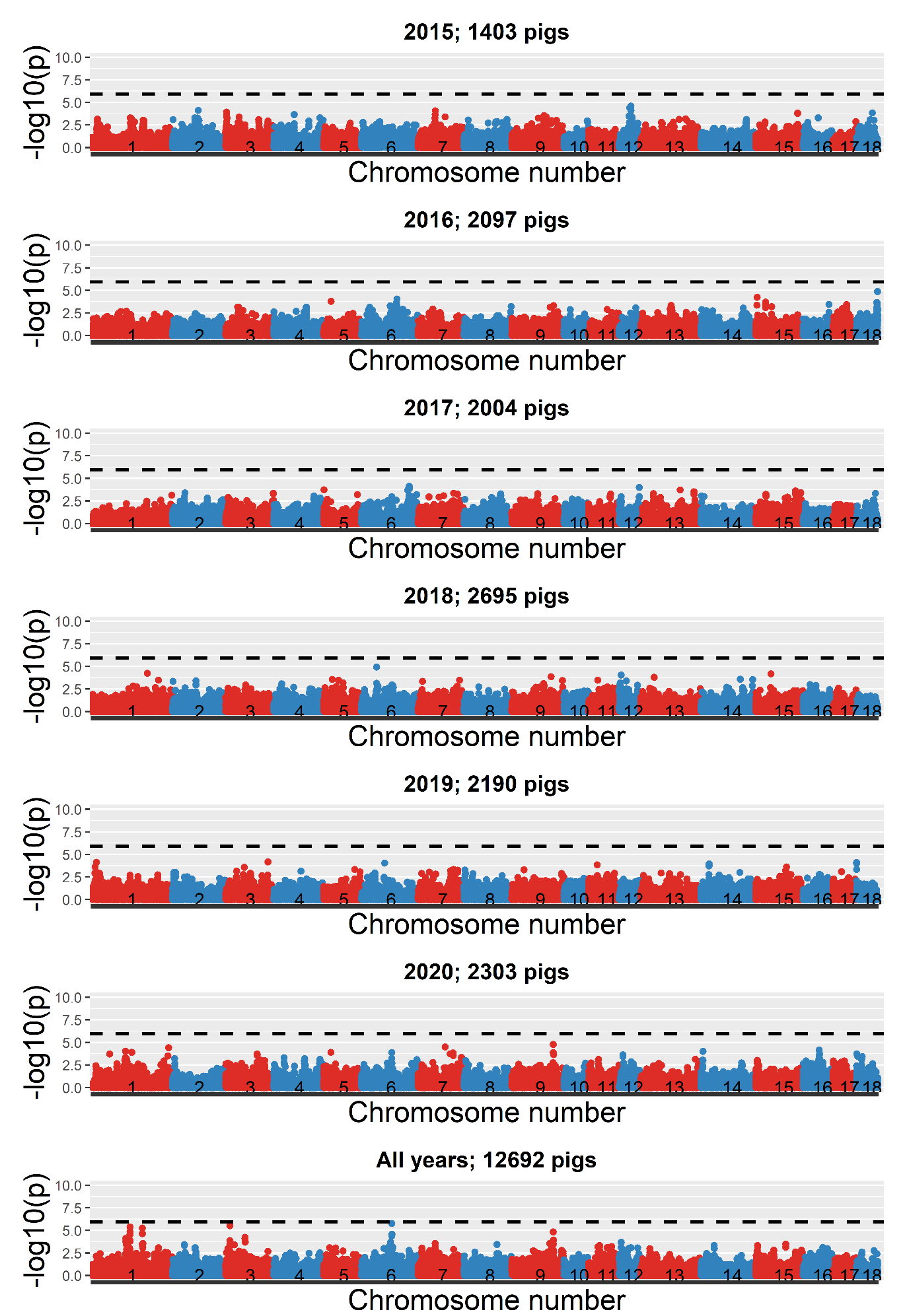


**Figure S5.4** Manhattan plots for total number born first parity in line A for the different years.


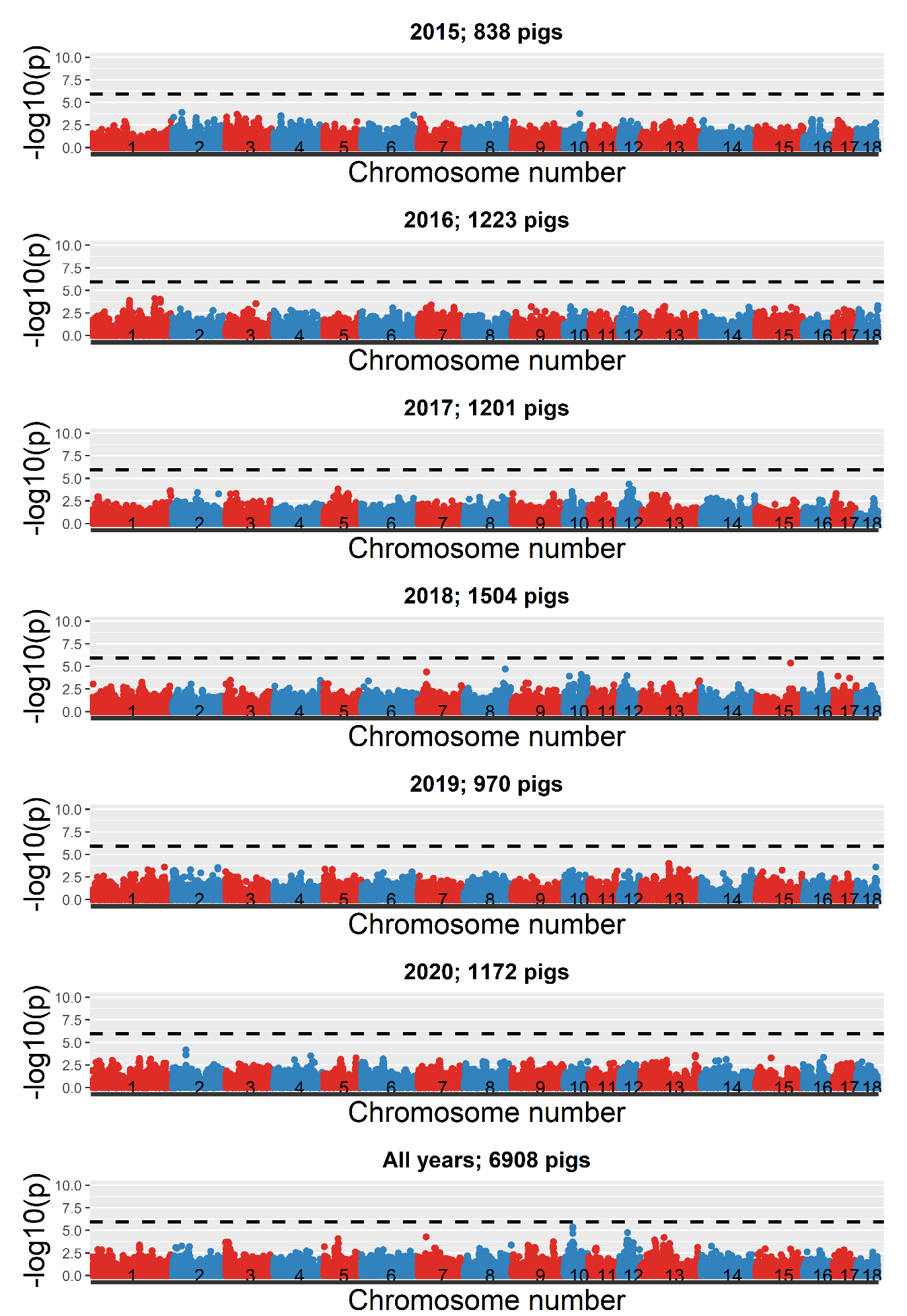


**Figure S5.5** Manhattan plots for average birth weight first litter in line A for the different years.


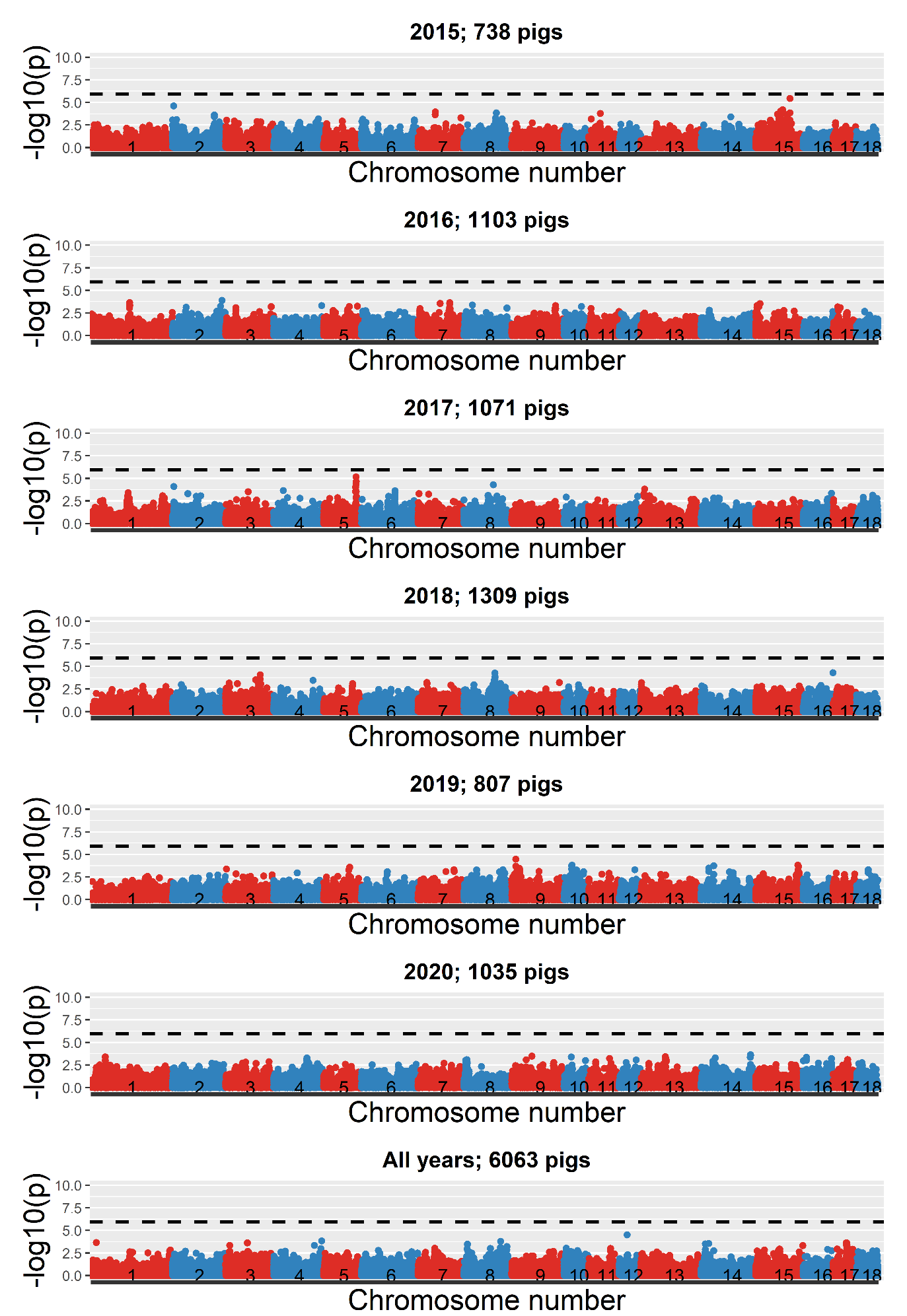


**Figure S5.6** Manhattan plots for CV of birth weight first litter in line A for the different years.


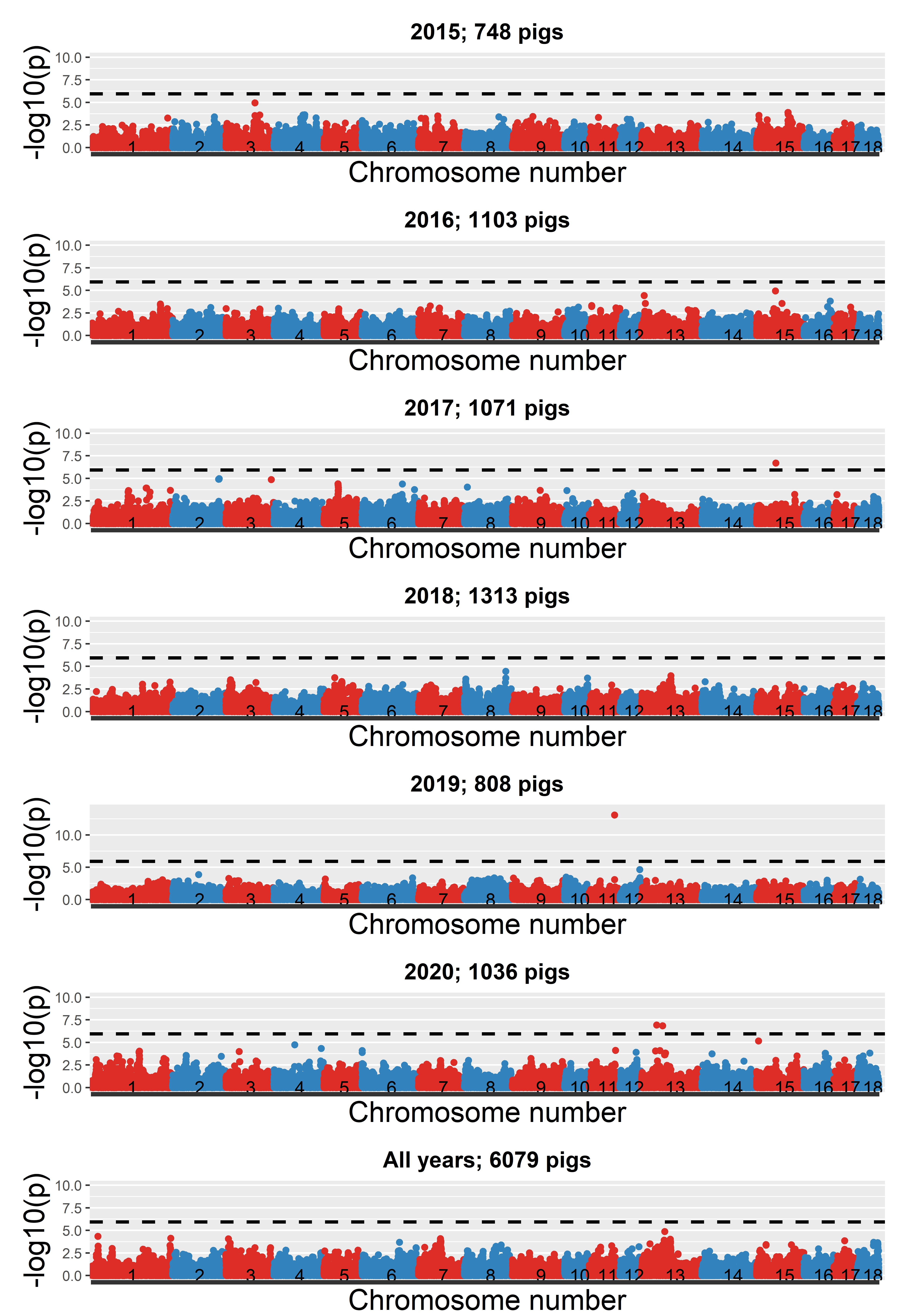


**Figure S5.7** Manhattan plots for number of small piglets in line A for the different years.


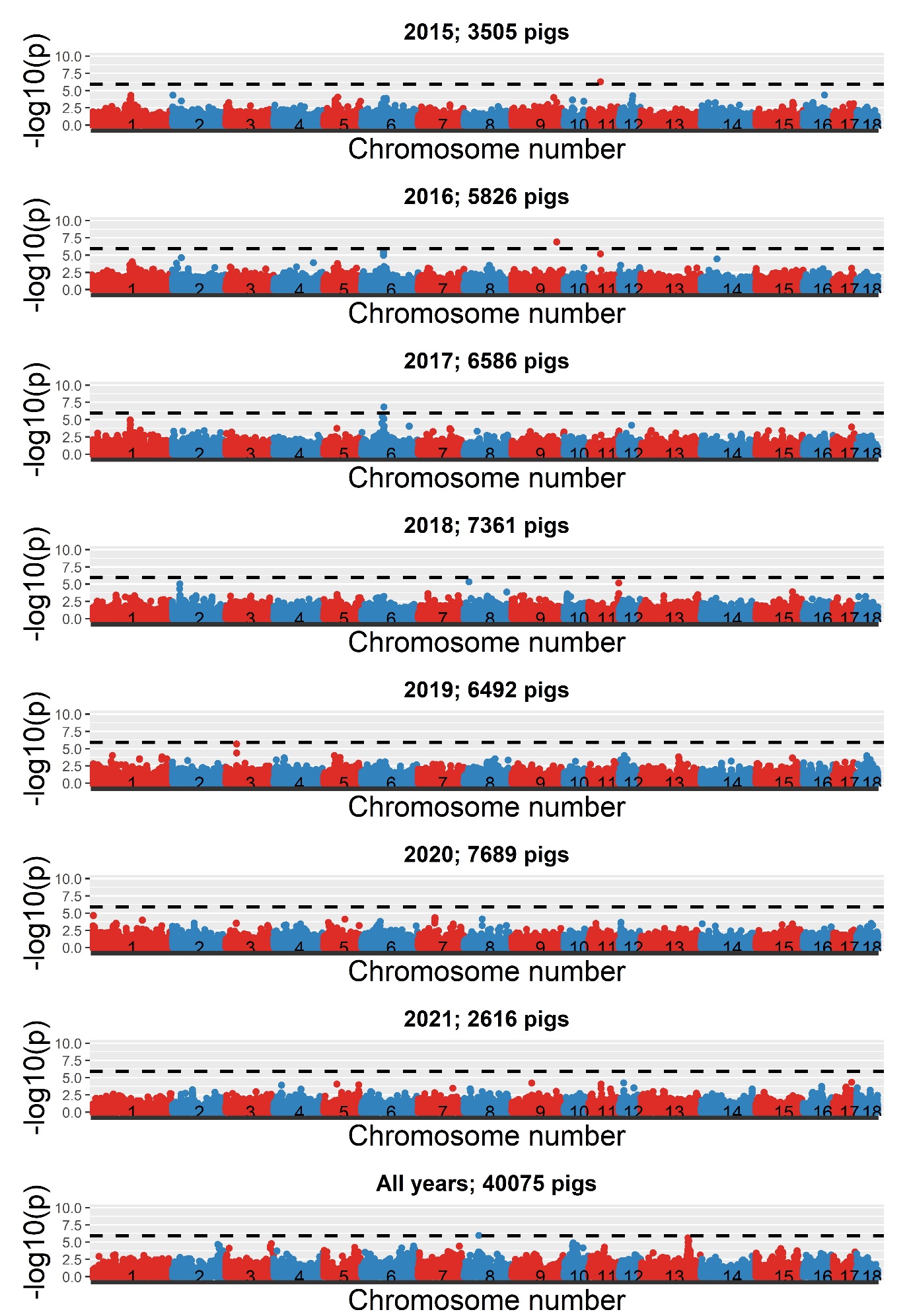


**Figure S5.8** Manhattan plots the index in line A for the different years.


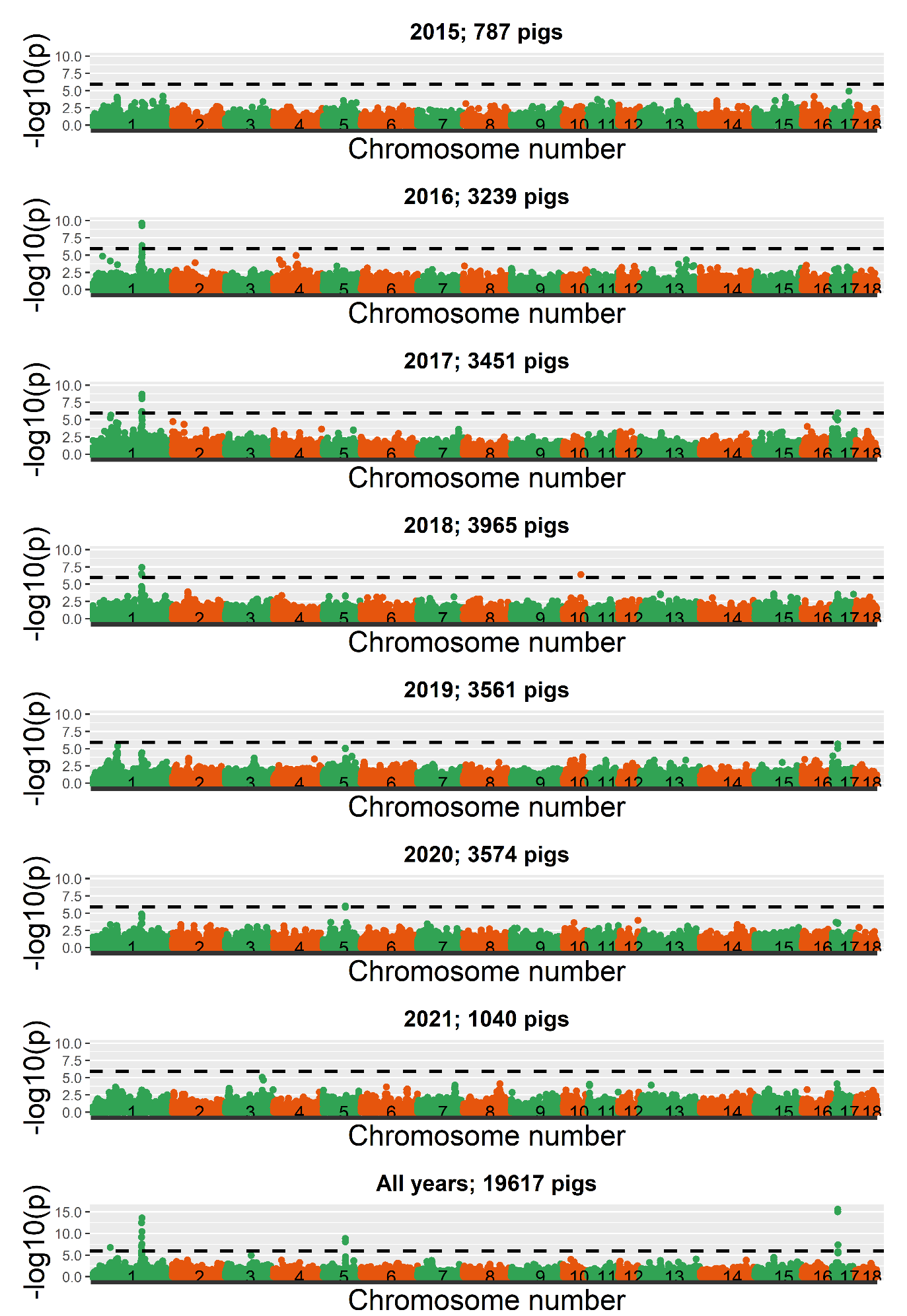


**Figure S5.9** Manhattan plots daily gain in line B for the different years.


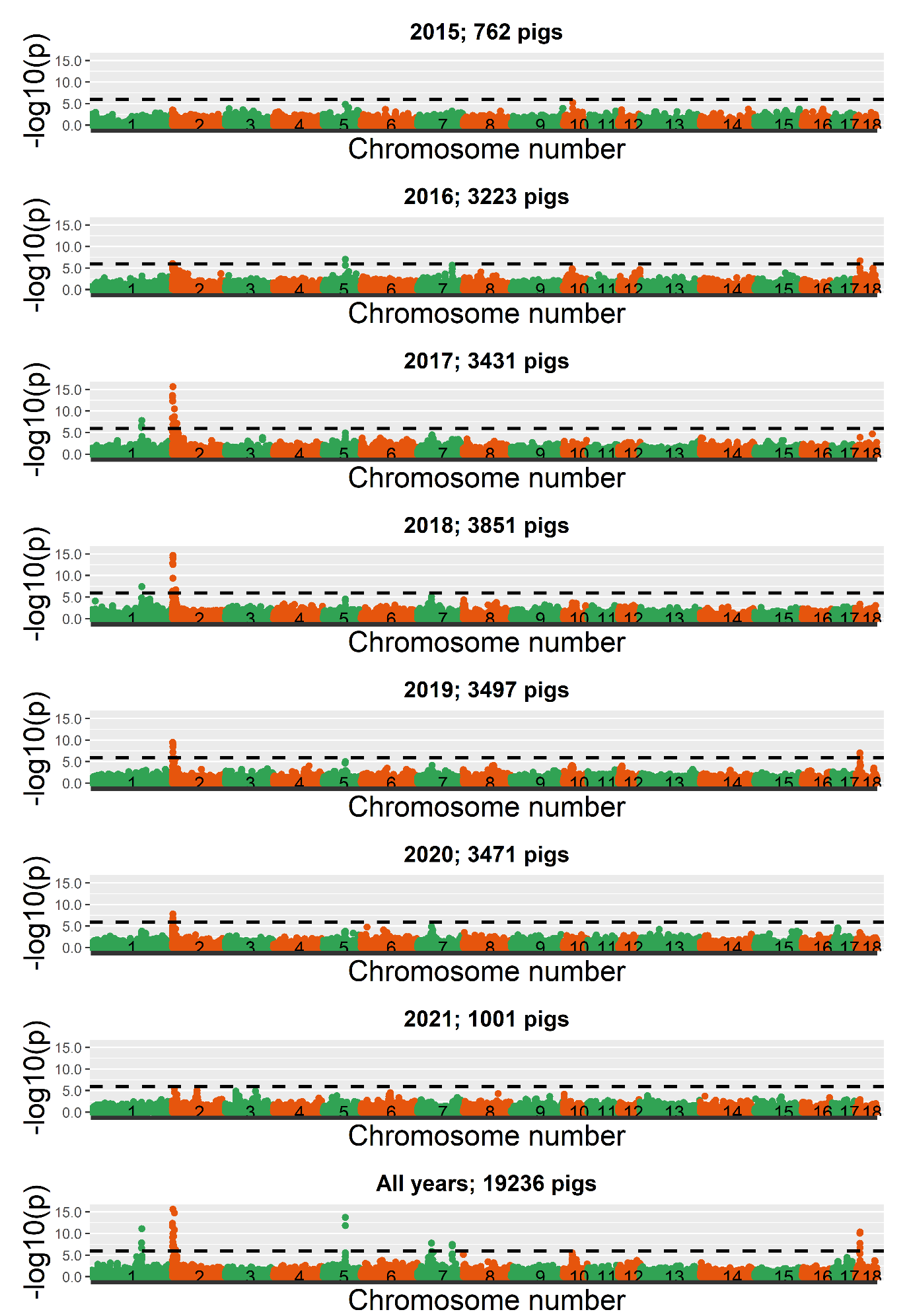


**Figure S5.10** Manhattan plots fat depth in line B for the different years.


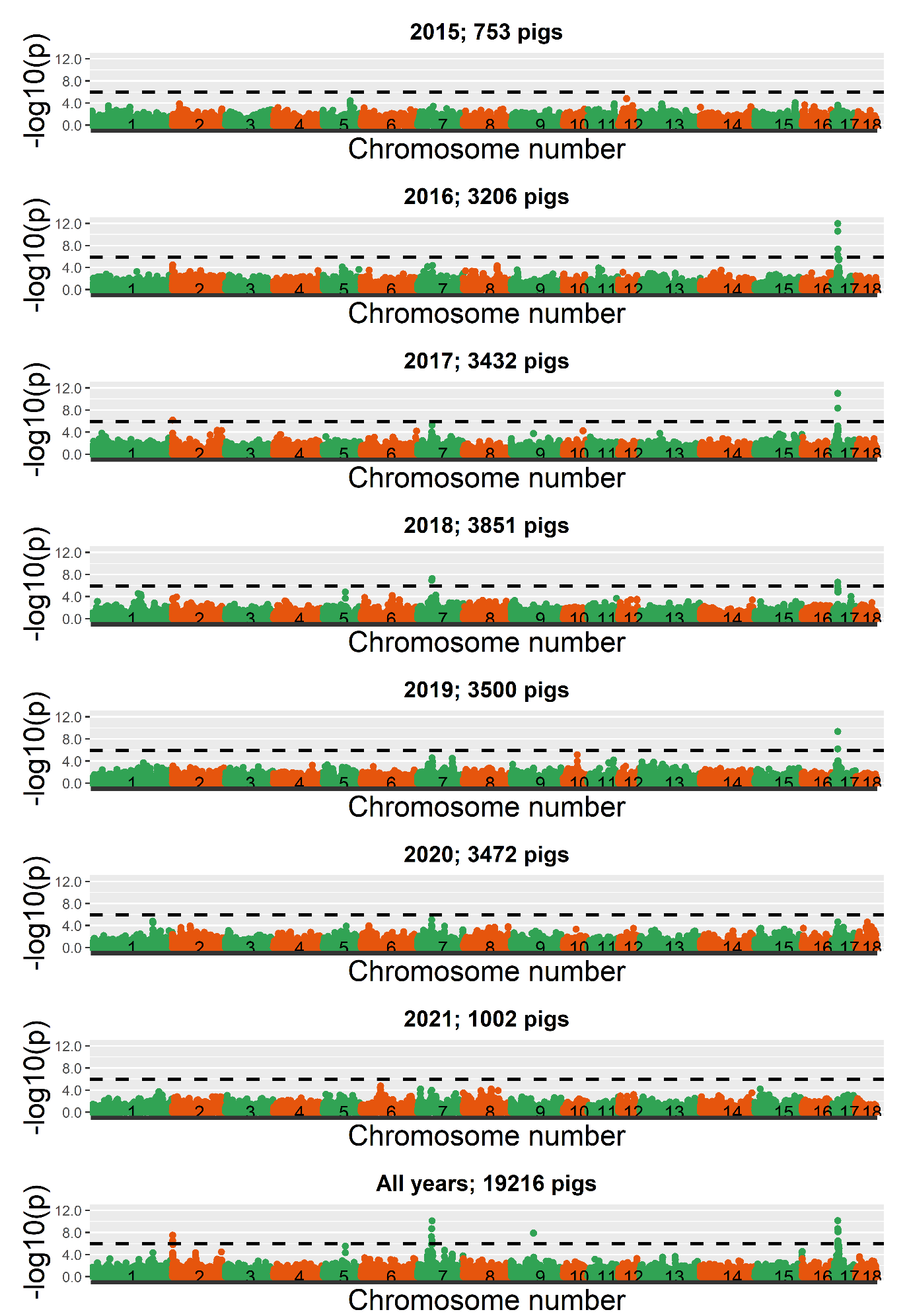


**Figure S5.11** Manhattan plots muscle depth in line B for the different years.


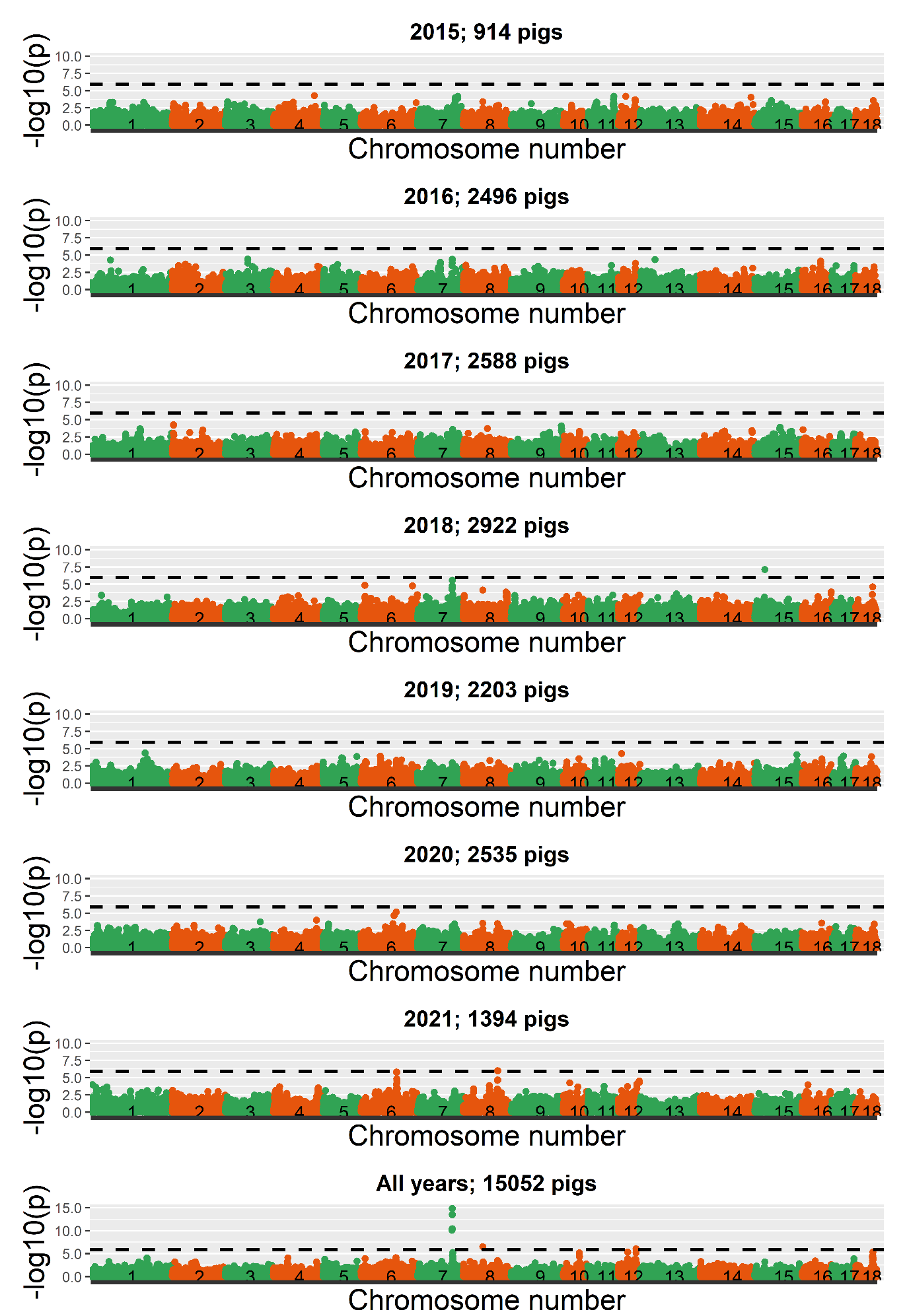


**Figure S5.12** Manhattan plots number of teats in line B for the different years.


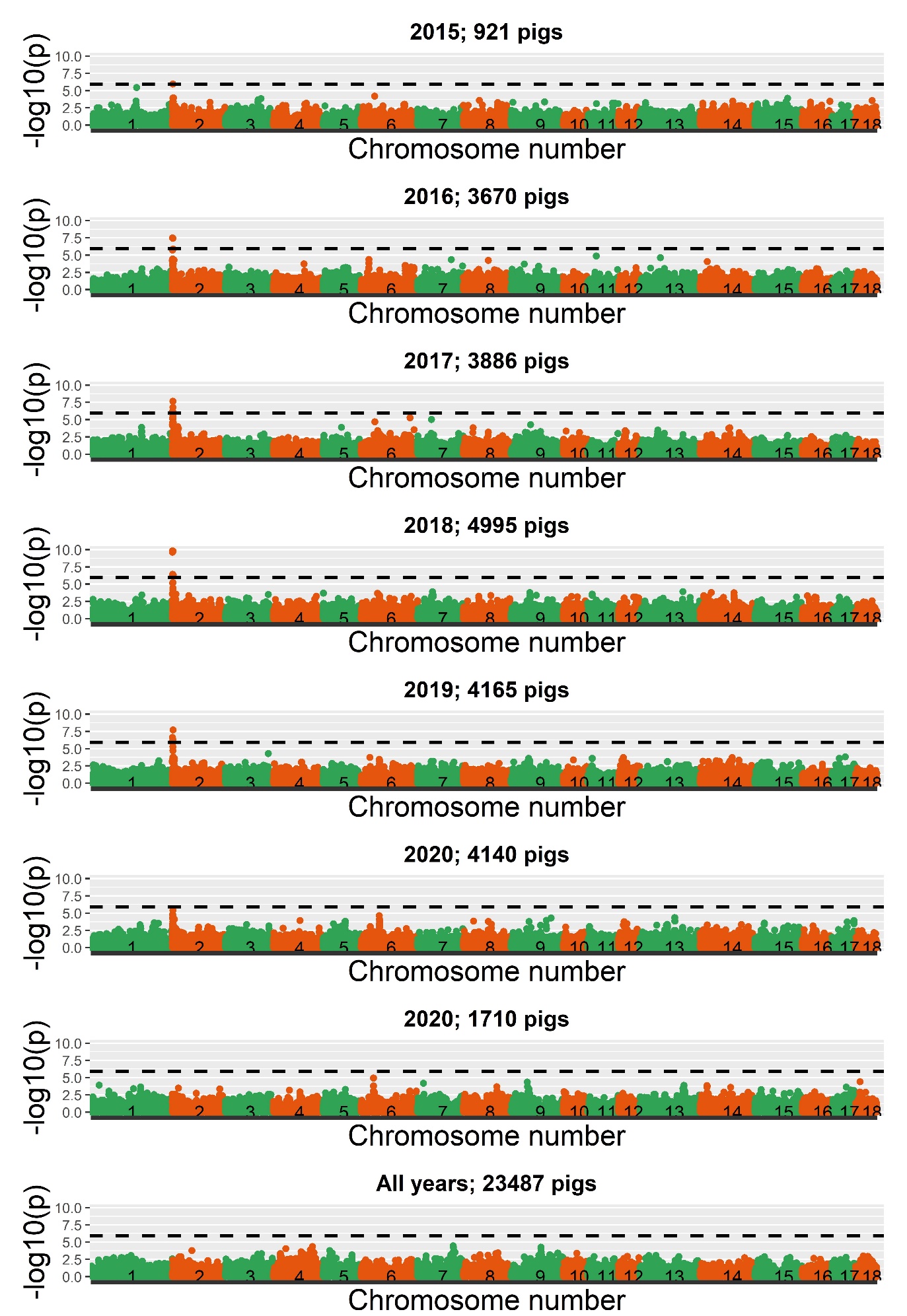


**Figure S5.13** Manhattan plots the index in line B for the different years.
